# Transcriptomic analysis of spinal V1 interneurons informs their multifunctional role in motor output

**DOI:** 10.1038/s41467-026-76522-3

**Published:** 2026-08-10

**Authors:** Alexandra J. Trevisan, Katie Han, Phillip D. Chapman, Anand S. Kulkarni, Jennifer M. Hinton, Ines Klein, Cody Ramirez, Alfonso Lavado, Graziana Gatto, Mariano I. Gabitto, Vilas Menon, Jay B. Bikoff

**Affiliations:** 1 Department of Developmental Neurobiology, St. Jude Children’s Research Hospital, Memphis, TN USA; 2 Department of Neurology, University Hospital of Cologne, Cologne, Germany; 3 Center for Pediatric Neurological Disease Research, St. Jude Children’s Research Hospital, Memphis, TN USA; 4 Allen Institute for Brain Science, Seattle, WA USA; 5 Department of Statistics, University of Washington, Seattle, WA USA; 6 Department of Neurology, Center for Translational and Computational Neuroimmunology, Columbia University, New York, NY USA

**Keywords:** Spinal cord, Neural circuits, Cell type diversity

## Abstract

Neural circuits in the spinal cord are composed of diverse populations of interneurons that play crucial roles in shaping motor output. However, the extent of interneuron heterogeneity and how this diversity relates to functional aspects of movement remain unclear. Here, through a focus on mouse spinal V1 interneurons, we show that loss of the V1 transcription factor En1 selectively disrupts the frequency of rhythmic locomotor output but does not disrupt flexion/extension limb movement, thereby decoupling two key functional roles ascribed to this neuronal population. To investigate the cellular basis of these deficits, we generated a single-nucleus transcriptomic atlas of V1 interneurons across postnatal development. Our analysis reveals age-dependent transcriptional changes while also demonstrating that their core molecular taxonomy perdures into adulthood. Notably, En1 deficiency selectively perturbed a single subset of V1^Pou6f2^ interneurons, thereby identifying a possible cellular substrate for influencing locomotor speed. Beyond serving as a molecular resource, our study highlights how deep neuronal profiling provides an entry point for understanding the multifunctional nature of heterogeneous interneuron populations.

## Abstract (web summary)

This study leverages transcriptomic profiling of spinal V1 interneurons to understand how they contribute to movement. By genetically targeting and manipulating these neurons, the authors decouple locomotor speed and limb flexion phenotypes, suggesting these key elements of motor control may be governed by different subsets of V1 interneurons.

## Introduction

Neural circuits in the spinal cord are tasked with generating precise patterns of muscle contraction that underlie behavior. To achieve coordinated motor output, the spinal cord relies on networks of interneurons that integrate supraspinal commands and sensory information to shape the temporal dynamics of motor neuron activity^1,2^. In this way, the motor system sets the tone for distinct components of movement, such as the speed of rhythmic locomotor activity, flexion and extension across joints, and inter-limb coordination that enables different locomotor gaits. Accordingly, substantial effort has been devoted to understanding the logic through which different types of spinal interneurons regulate movement. Early studies relied primarily on anatomical and electrophysiological properties to define several canonical interneuron types, including Group Ia interneurons that mediate reciprocal inhibition of antagonistic motor neurons, Renshaw cells responsible for the recurrent inhibition of motor neurons, and presynaptic inhibitory interneurons that gate incoming sensory input^3–7^. More recently, molecular and genetic approaches to neural circuit dissection have defined a core cellular taxonomy in which cardinal classes of ventral interneurons, termed V0 to V3, can be differentiated based on developmental provenance and gene expression^1,8,9^. This knowledge, in turn, has provided insight into the functions of major neuronal populations in limb movement, most prominently in the context of locomotion^1,2,10–15^. Yet it has become increasingly clear that each neuronal class is itself highly heterogeneous^16,17^, raising the challenge of understanding how the spinal motor system takes advantage of diverse sets of interneurons to shape motor output.

Among ventral neuronal classes, V1 interneurons constitute a heterogeneous population of inhibitory neurons that form a core part of the spinal motor infrastructure^18,19^. Notably, ablation or inhibition of V1 interneurons severely perturbs motor function, resulting in multiple behavioral phenotypes including slowed rhythmic locomotor output and limb hyperflexion^11,15,20,21^. Despite their important role in motor control, whether and how different V1 subsets contribute to specific elements of motor output is unclear. This challenge is compounded by our lack of understanding regarding the core molecular distinctions between V1 subsets and the underlying mechanisms through which V1 heterogeneity arises. We reasoned that a transcriptomic dissection of V1 interneuron diversity, combined with an exploration of the behavioral consequences of perturbing V1 interneuron identity, would inform our understanding of the multifunctional roles of spinal interneurons in motor output.

Here, taking advantage of mouse genetics, physiology, and behavioral analysis, we show that the two functional roles assigned to the V1 population - the control of limb flexion/extension and the speed of rhythmic locomotor output - can be separated from one another upon deletion of the transcription factor En1, which marks the V1 population^22^. To gain insight into the cellular basis underlying the different functional roles of these neurons, we used single-nucleus RNA-sequencing (snRNA-seq) to perform a deep transcriptomic analysis of V1 interneurons across postnatal development and into adulthood. Targeted enrichment of V1 interneurons enabled snRNA-seq of more than 89,000 nuclei, providing unparalleled single-cell resolution of an individual spinal interneuron class and its molecular subgroups, which can be explored through an interactive website (https://v1interneurons.stjude.org/shinyApp/). This analysis revealed a previously unknown V1 subset, identified the repertoire of transcription factors (TFs) and ion channels that characterize distinct V1 subsets, and demonstrated that V1 cell type identity is conserved from the initial establishment of neural circuit formation to the mature nervous system. Importantly, snRNA-seq analysis of V1 interneurons isolated from *En1* null mice uncovered a key role for En1 in specifying a highly restricted subset of V1^Pou6f2^ interneurons, providing insight into the underlying mechanisms that may contribute to altered rhythmic locomotor speed. Beyond serving as a molecular resource for V1 interneurons, including for comparative studies focused on evolutionary conservation and species-specific innovations in spinal interneuron identity^23^, our work highlights the use of deep transcriptomic profiling to better understand the link between spinal interneuron heterogeneity and distinct aspects of motor output.

## Results

### Loss of spinal En1 expression perturbs specific elements of motor output

**Fig. 1:**
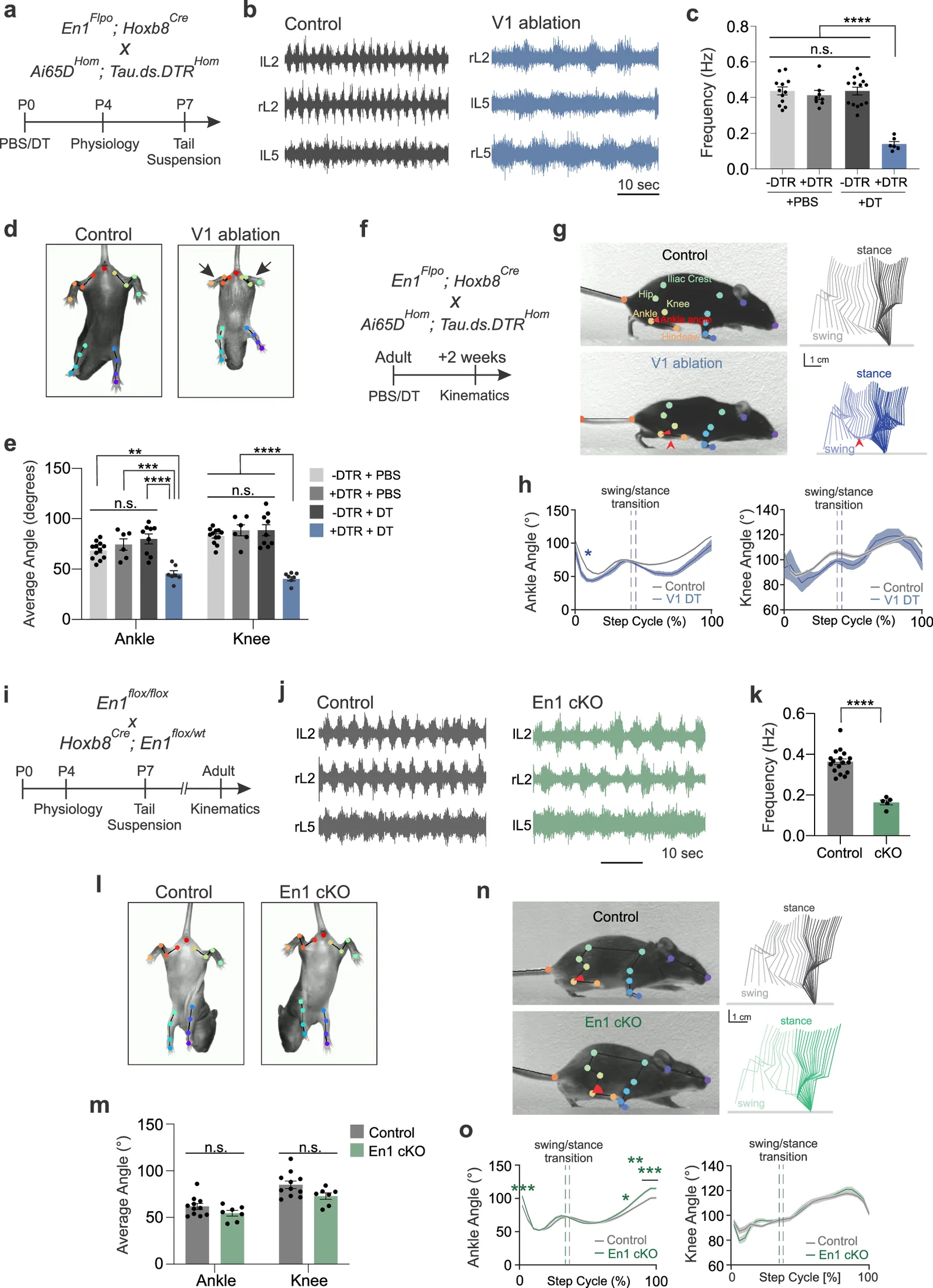
Loss of spinal En1 expression reduces the frequency of fictive locomotion but does not result in limb hyperflexion. **a** Schematic of experimental paradigm used for physiological and behavioral analyses of V1 ablated mice in panels (**b**–**e**). **b** Ventral root recordings during fictive locomotion performed on P4 control (-DTR + DT, gray) or V1 ablated (+DTR + DT, blue) spinal cords. **c** Reduced frequency of fictive locomotor activity upon DT-mediated ablation of V1 interneurons (*n *= 12, 8, 14, and 6 mice, respectively; mean ± SEM, n.s. = not significant, ****p < 0.0001, two-way ANOVA followed by Holm-Sidak test for multiple comparisons). **d** Tail suspension assay performed in P7 control (-DTR + DT, left) or V1 ablated (+DTR + DT, right) mice. Hindlimb hyperflexion is indicated by arrows. **e** Average angles of the ankle and knee joints are significantly reduced upon ablation of V1 interneurons, indicating a hyperflexed state (*n *= 12, 6, 9, and 7 mice, respectively; mean ± SEM, **p < 0.01, ***p < 0.001, ****p < 0.0001, two-way ANOVA followed by Holm-Sidak test for multiple comparisons). **f** Schematic of experimental paradigm for assessing locomotor kinematics in vivo. **g** Images of control (top left) and V1 ablated (bottom left) mice crossing a catwalk, with landmarks tracked using DLC. Right, stick diagrams of hindlimb landmarks during a step of control (top right) or V1 ablated (bottom right) mice. Hyperflexion of the ankle during swing is indicated by the red arrowhead. **h** Line graphs showing the average ankle (left) and knee (right) angle changes during a normalized step cycle in control (gray, *n *= 21) and V1 ablated (blue, *n* = 4) mice. Means were compared using a two-way ANOVA followed by the Holm-Sidak post-hoc test. SEMs are represented as shaded areas. *p < 0.05. **i** Schematic of the experimental paradigm for physiological and behavioral analyses of *En1* conditional knockout (*En1 cKO*) mice. Littermates of all other genotypes were pooled as controls. **j** Ventral root recordings in control (*En1*^*flox/+*^, gray) or *En1 cKO* (*Hoxb8*^*Cre*^*; En1*^*flox/flox*^, green) mice. **k** *En1 cKO* mice show reduced locomotor frequency (*n* = 17 and 5 mice, respectively; mean ± SEM, ****p < 0.0001, unpaired two-tailed *t*-test). **l** Hindlimbs of control (*En1*^*flox/+*^) or *En1 cKO* mice observed during tail suspension assay, with normal flexion/extension. **m** Average angles of ankle and knee joints are not significantly different (*p* = 0.1360 and *p* = 0.0502, respectively, unpaired two-tailed *t*-test) upon loss of En1 (*n* = 11 and 7 mice, respectively; mean ± SEM). **n** Images of control (top left) and *En1 cKO* (bottom left) mice crossing a catwalk, with landmarks tracked using DLC. Right, stick diagrams of hindlimb landmarks during a step of control (top right) or V1 *En1 cKO* (bottom right) mice. **o** Line graphs showing the average ankle and knee angle changes during a normalized step cycle in control (gray, *n* = 22) and *En1 cKO* (green, *n* = 13) mice, as in (**h**). Asterisks indicate a hypoflexed state (two-way ANOVA followed by the Holm-Sidak post-hoc test). *p < 0.05, **p < 0.01, ***p < 0.001). See Supplementary Table 2 for statistics of all comparisons. Source data are provided as a Source Data file.

Given the multifunctional role of V1 interneurons in shaping motor output, we considered whether the effects of V1 interneurons on limb flexion and the frequency of rhythmic locomotor output are linked, or whether these two phenotypes can be dissociated from one another. Toward this end, we first confirmed that acute loss of V1 interneurons perturbs both locomotor frequency and limb flexion, as reported previously^20^. To ablate spinal V1 interneurons while avoiding supraspinal *En1* expressing structures, we took advantage of an intersectional strategy in which *En1*^*Flpo*^ is combined with *Hoxb8*^*Cre*^ to limit expression of the diphtheria toxin receptor (DTR) in *Tau.ds.DTR* mice to the spinal cord, as has been successfully implemented previously (Fig. 1a, Supplementary Fig. 1a, b)^20^. Ablation of V1 interneurons via administration of diphtheria toxin (DT) to *En1*^*Flpo*^*; Hoxb8*^*Cre*^*; Ai65D; Tau.ds.DTR* mice significantly slowed locomotor rhythm, as measured with ventral root recordings during fictive locomotion (Fig. 1b, c). V1 ablated animals also showed severe hyperflexion of their hindlimbs, as assessed using DeepLabCut (DLC) and AutoGaitA to quantify ankle and knee angle during the tail suspension assay (Fig. 1d, e and Supplementary Video 1)^24,25^. In contrast, the forelimbs in V1 ablated animals did not exhibit hyperflexion (Supplementary Fig. 1c), consistent with the incomplete recombination efficiency of *Hoxb8*^*Cre*^ in more rostral (cervical) spinal segments^26^. Kinematic analysis performed during locomotion in adult V1 ablated mice likewise revealed ankle hyperflexion during the swing phase of the step cycle (Fig. 1f, g and Supplementary Video 2), exemplified by the reduced angle of the ankle and the increased height of the hindpaw resulting from the hyperflexed state (Fig. 1h and Supplementary Fig. 1d).

Notably, homozygous *En1*^*taulacZ*^ mice lacking En1 protein also exhibit slowed rhythmic locomotor-like output^11^, raising the question of whether loss of the En1 transcription factor itself fully phenocopies ablation of V1 interneurons by also affecting limb flexion. To confirm that the loss of En1 affects the speed of rhythmic locomotor activity, we took advantage of the fact that the *En1*^*Cre*^ allele disrupts the endogenous *En1* locus^27^, thereby creating a null allele and generating *En1* knockout (*En1*^*KO*^) animals in the homozygous state. Upon recording ventral root activity during fictive locomotion in P0 spinal cords, we observed a dramatic reduction in frequency in *En1*^*KO*^ mice relative to *En1*^*Het*^ animals (Supplementary Fig. 1f). Due to neonatal lethality observed in *En1*^*KO*^ mice, which lack En1 expression globally, we were unable to properly assess limb flexion in these animals. To bypass this neonatal lethality, we generated *Hoxb8*^*Cre*^*; En1*^*flox/flox*^ conditional knockout mice (hereafter called *En1 cKO* mice) in which deletion of En1 was restricted to the spinal cord (Fig. 1i). Immunohistochemical analysis confirmed that *En1 cKO* animals showed a loss of En1 protein in lumbar spinal segments (Supplementary Fig. 1 g, h). Like V1 ablated and *En1*^*KO*^ animals, *En1 cKO* animals showed a reduced frequency of locomotor-like activity during fictive locomotion (Fig. 1j, k). Importantly, however, *En1 cKO* animals exhibited normal hindlimb position during the tail suspension assay, showing none of the severe hyperflexion characteristics of V1 ablated animals (Fig. 1l, m and Supplementary Video 1). Consistent with this, kinematic analysis performed during locomotion in *En1 cKO* animals showed an absence of a hyperflexed state, with limb position characterized instead by a slight hypoflexion (Fig. 1n, o; see also Supplementary Fig. 1e and Supplementary Video 2). Finally, to determine whether slowed locomotor-like activity is a general consequence of disrupting the V1 population, we assessed rhythmic activity following ablation of V1^Foxp2^ interneurons, a large subset that represents over half of V1 interneurons in V1^Foxp2^ lineage-traced mice^28^. Unlike ablation of the entire V1 population (Fig. 1b, c and Supplementary Fig. 1i–k), the speed of rhythmic activity in the isolated spinal cord was normal in V1^Foxp2^-ablated animals (Supplementary Fig. 1l–n), indicating a non-V1^Foxp2^ subset likely controls locomotor speed. Taken together, these data demonstrate that loss of En1 causes a subset of the phenotypes observed upon ablation of V1 interneurons, thereby dissociating deficits in locomotor speed from perturbations in flexion/extension movement.

### Generation and validation of a V1 transcriptomic atlas

**Fig. 2:**
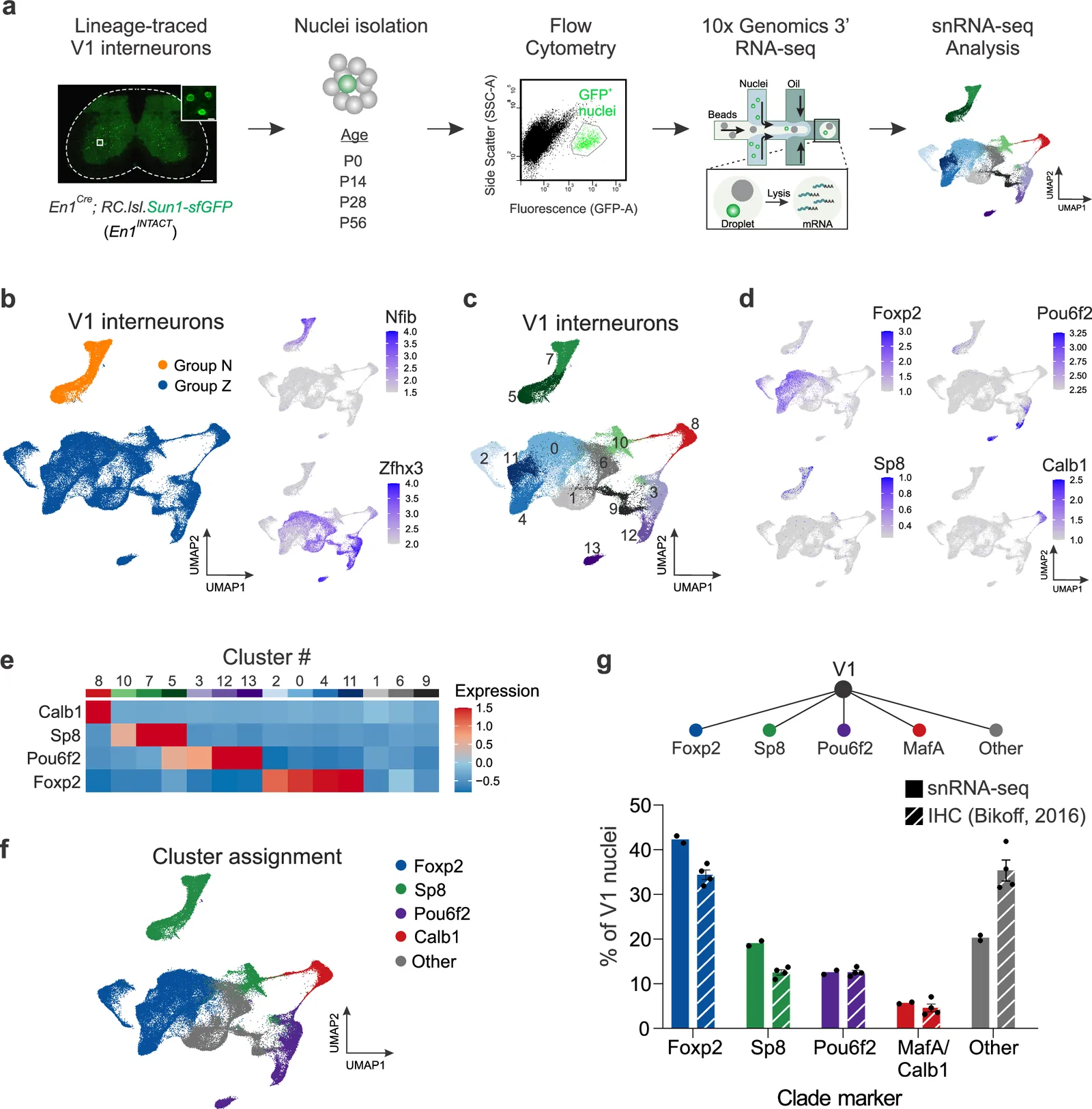
Single-nucleus transcriptomic profiling identifies V1 clades as molecularly distinct subsets. **a** Schematic of the experimental design for single-nucleus transcriptomic analysis of V1 interneurons from *En1*^*Cre*^*; RC.lsl.Sun1-sfGFP* double heterozygous mice. **b** Unsupervised clustering of V1 interneuron nuclei at low resolution reveals the first division corresponds to V1 interneurons expressing either Nfib (Group N) or Zfhx3 (Group Z). All UMAP plots depicting gene expression display log normalized values. **c** Unsupervised clustering of V1 interneuron nuclei at higher resolution identifying 14 clusters. **d** UMAP showing expression of the V1 clade markers Foxp2, Pou6f2, Sp8, and Calb1 (a proxy for MafA), which largely segregate in UMAP space. **e** Heatmap showing the scaled average expression per cluster of V1 clade markers. **f** Assignment of clade identity based on expression of Foxp2, Sp8, Pou6f2, and Calb1. **g** Comparison of the proportion of V1 interneurons contained within the clusters assigned to a given clade versus the proportion previously measured by immunohistochemical analysis of P0 lumbar spinal cord, where V1^Foxp2^ = 42.3% vs 34.4% ± 1.1%, V1^Sp8^ = 19.1% vs 12.5% ± 0.6%, V1^Pou6f2^ = 12.6% vs 12.6% ± 0.4%, and V1^MafA/Calbindin1^ = 5.7% vs 4.6% ± 0.9%; mean (transcriptomics) or mean ± SEM (IHC); *n* = 2 replicates (transcriptomics) or 4 mice (IHC). All immunohistochemical data is from Bikoff et al.^18^ Source data are provided as a Source Data file.

To begin to understand the basis of these behavioral effects, we undertook a deep transcriptomic analysis of V1 interneurons across postnatal development, reasoning that an unbiased assessment of V1 diversity via snRNA-seq would provide a foundation from which to consider the cellular substrates underlying these alterations in motor output. Analogous single-cell transcriptomics approaches have proven crucial to resolving molecular differences in cell type identity across the nervous system, including in the spinal cord^29–35^. Given that V1 interneurons constitute only a small fraction of the overall cellular ecosystem in the spinal cord^29^, we devised a genetic strategy to selectively enrich for lineage-traced V1 interneurons, opting to perform single-nucleus rather than single-cell analysis due to several advantages: (1) Single-nucleus analysis can be performed on postnatal and adult spinal cord tissue, from which viable cells are difficult to isolate^33^; (2) it avoids experimental artifacts from stress-induced transcriptional changes that occur within intact cells during dissociation^36^; and (3) it is an effective means of accurately resolving cell-type identity^37^. We used the INTACT (isolation of nuclei tagged in specific cell types) method, in which GFP^+^ V1 nuclei were isolated by fluorescence-activated nuclei sorting (FANS) from *En1*^*Cre*^*; RC.lsl.Sun1-sfGFP* double heterozygous mice (hereafter called *En1*^*INTACT*^ mice) at four postnatal stages (P0, P14, P28, and P56) (Fig. 2a)^38^. This resulted in an ~60-fold enrichment in GFP^+^ V1 nuclei relative to the pre-sorted population (Supplementary Fig. 2a–c).

We next performed droplet-based snRNA-seq using the 10x Genomics platform (v3.1, see Supplementary Data 1 for CellRanger metrics). Although our FANS approach yielded strong enrichment of V1 nuclei, we still needed to ensure that only V1 interneurons were included in the subsequent analysis; thus, we developed a rigorous pipeline in which V1 nuclei were identified by multiple iterations of both negative and positive selection criteria (summarized in Supplementary Fig. 2d; Methods). After selecting nuclei that passed quality control metrics, we employed iterative negative selection criteria to remove contaminating nuclei corresponding to non-V1 populations, such as oligodendrocytes, astrocytes, vascular cells, and non-V1 neuronal populations (Supplementary Fig. 2e–h; see Supplementary Data 2 for a list of marker genes). In parallel, we used a previously published embryonic whole spinal cord reference dataset to annotate postnatal nuclei. Toward this end, we collected and profiled V1 nuclei from embryonic day (E) 13.5 spinal cords of *En1*^*INTACT*^ mice to obtain an age-matched sample for comparison to the reference scRNA-seq dataset containing clearly annotated V1 interneurons with *En1* expression^29^. Using the Seurat label-transfer algorithm, we calculated a V1 prediction score and identified embryonic nuclei that were assigned a putative V1 identity (Supplementary Fig. 2i–n). Comparison of the putative V1 nuclei in our postnatal dataset with the reference V1 nuclei in the embryonic data set enabled us to positively identify postnatal V1 nuclei with high confidence.

In total, we isolated 89,494 V1 nuclei that passed selection criteria across two independent replicates of four postnatal ages (Fig. 2 and Supplementary Fig. 3a; see Supplementary Data 3 for unique barcodes for all cells included in the final figures). These nuclei contained a mean of 6356 unique molecular identifiers (UMIs) and 2622 unique genes per nucleus (Supplementary Fig. 3b–d). To overcome batch effects arising from experimental sources of variability, we integrated the data using the Seurat anchor-based workflow, followed by dimensionality reduction and clustering to identify communities of cells^39^. Unsupervised clustering at a low resolution and visualization in uniform manifold approximation and projection (UMAP) space revealed that the first division corresponds to V1 interneurons expressing either Nfib (Group N) or Zfhx3 (Group Z), consistent with prior observations that a basic axis of organization for ventral interneurons centers on medial, late-born, local-projecting (Group N) neurons versus lateral, earlier-born, long-distance projecting (Group Z) neurons (Fig. 2b)^40^. To identify cell-type diversity at a more refined level, we subjected our V1 dataset to higher resolution clustering and identified 14 distinct clusters that ranged in size from 22.9% (Cluster #0) to 2.2% (Cluster #13) of the larger V1 population (Fig. 2c and Supplementary Fig. 3f).

We previously found that V1 interneurons segregate into at least four mutually exclusive subsets (clades) based on immunohistochemical analysis of the TFs Foxp2, MafA, Pou6f2, and Sp8^18^. Accordingly, we next explored whether clades delineated on the basis of single TFs indeed represent distinct clusters when assessed by their overall transcriptomic profiles. *Foxp2*, *Pou6f2*, and *Sp8* were detected at sufficient levels to assign cluster identity based on the z-score of the average expression per cluster (Fig. 2d–f), with *Foxp2* (clusters #0, #2, #4, and #11) and *Pou6f2* (clusters #3, #12, #13) robustly detected in postnatal V1 interneurons, and *Sp8* (clusters #5, #7, and #10) more weakly detected. Due to low detection of *MafA*, *Calbindin1* (*Calb1*) was used as a proxy marker for the *MafA* clade (cluster #8), which likely corresponds to Renshaw cells. Notably, these clades represented almost completely mutually exclusive clusters, with only cluster #5 showing some degree of *Pou6f2* and *Sp8* co-expression (Fig. 2e), indicating that a small fraction of V1^Sp8^ and V1^Pou6f2^ neurons may be similar at the molecular level. Consistent with our prior findings, the proportion of each V1 clade in our snRNA-seq data (assessed based on cluster assignment rather than per nucleus to account for the high dropout rate of snRNA-seq) was comparable to those assessed via immunohistochemical analysis (Fig. 2g). Importantly, independent validation of our single-nucleus data at the transcriptomic level using multiplexed RNAScope to detect *Calb1*, *Foxp2*, *Pou6f2*, and *Sp8* confirmed that these key markers are expressed within V1 interneurons in a mutually exclusive manner (Supplementary Fig. 4a–c)^18^.

In prior work that relied on TF co-expression data and spatial information, we used a sparse Bayesian framework to infer the existence of multiple cell types within each V1 clade^19^. Therefore, we asked whether this general logic was observed in our snRNA-seq data, and used the V1^Sp8^ clade as a test case, focusing on the expression of the Sp8-associated TFs Prox1, Prdm8, and Oc2, and St18, a TF implicated in specifying projection neuron identity in the globus pallidus^41^ (Supplementary Fig. 5). This analysis revealed two clusters (#5 and #7) with various combinations of *Prox1*, *Prdm8*, and *St18* expression, and a single cluster (#10) expressing *Oc2* but lacking *Prox1*, *Prdm8*, and *St18*, demonstrating general alignment with predictions from the Bayesian model, albeit with fewer identified cell types (Supplementary Fig. 5b–f). Given the discrete nature of the V1^Sp8/Oc2^ cluster, we sought to validate its presence via immunohistochemical analysis. Although we observed few V1^Sp8^ neurons expressing Oc2 (2.3% ± 0.4%) in mid-lumbar (L3/L4) spinal segments of P0 mice, approximately 40% of all V1^Sp8^ neurons in L5 segments were positive for Oc2, confirming the co-expression patterns observed in this V1^Sp8^ cluster and highlighting rostrocaudal distinctions in interneuron identity (Supplementary Fig. 5g)^42,43^. Together, these data show that V1 clades distinguished by *Foxp2*, *Pou6f2*, *Sp8*, and *MafA/Calbindin* expression represent molecularly distinct V1 cell types.

### Identification of an additional V1 subset

**Fig. 3:**
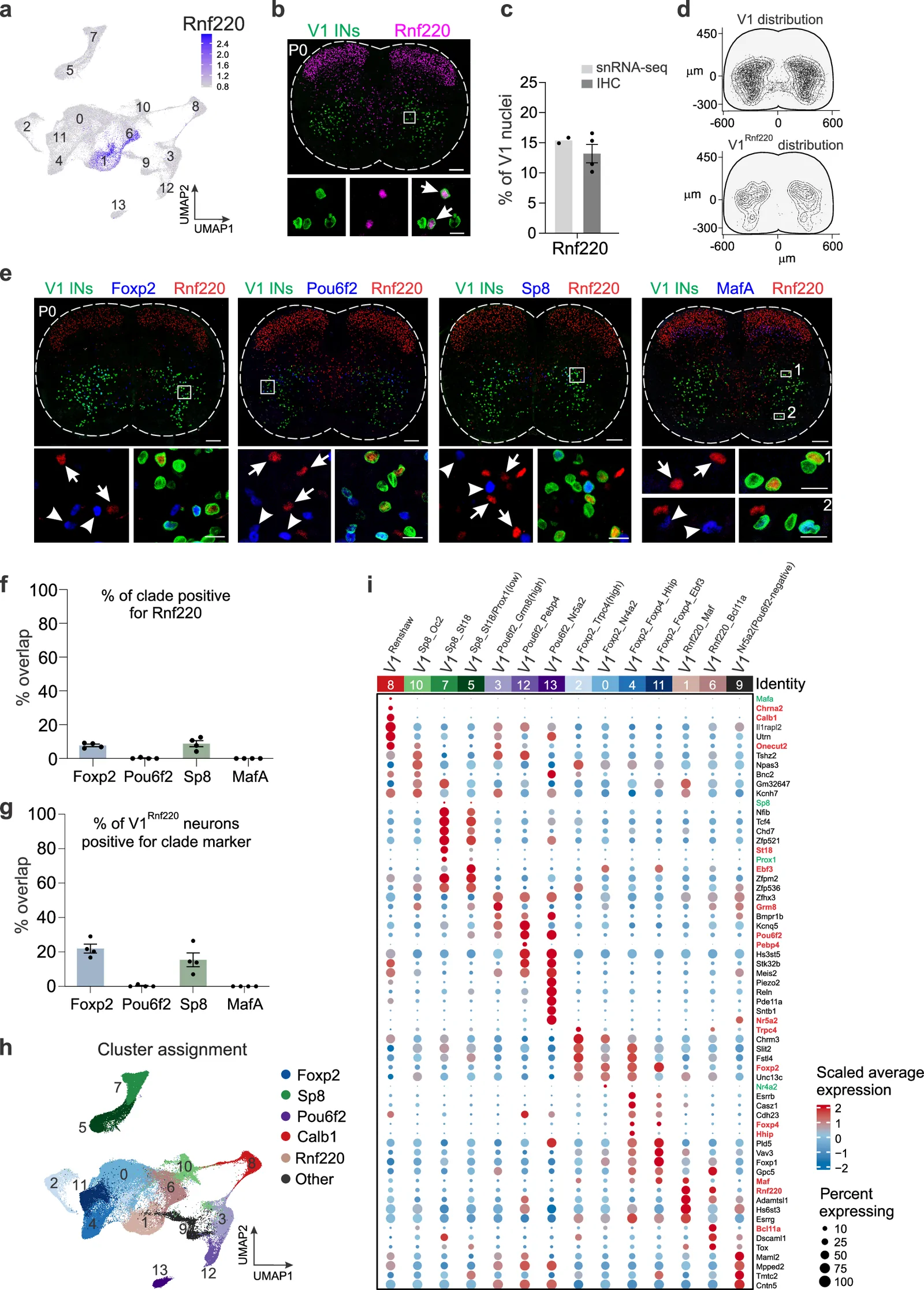
Identification of an additional V1 interneuron subset. **a** Expression of *Rnf220* enriched in clusters #1 and #6, which are not defined by the other four clade markers. **b** Immunohistochemical analysis of Rnf220 expression in lumbar spinal cord of P0 *En1*^*INTACT*^ mice, demonstrating expression in V1 interneurons (arrows). Scale bar = 100 μm (top) or 20 μm (bottom). **c** Fraction of V1^Rnf220^ interneurons in P0 lumbar spinal cord assessed by the proportion of V1 interneurons in Rnf220^+^ clusters #1 and #6 (14.9%) or via immunohistochemistry (13.2% ± 1.5%); mean (transcriptomics) or mean ± SEM (IHC); *n* = 2 snRNA-seq replicates or 4 mice, respectively. **d** Spatial distribution of V1^Rnf220^ interneurons largely recapitulates the distribution of the overall V1 population. **e** Immunohistochemical analysis and (**f**, **g**) quantification demonstrating Rnf220-expressing V1 interneurons represent a largely non-overlapping population with the four major clades, completely distinct from V1^Pou6f2^ and V1^MafA^ clades and exhibiting modest overlap with V1^Foxp2^ and V1^Sp8^ clades (*n* = 4 mice, mean ± SEM). Arrows indicate Rnf220 expression in V1 interneurons, and arrowheads indicate expression of other clade markers. Scale bars = 100 μm (top) or 20 μm (bottom) (**h**) Assignment of V1^Rnf220^ clusters relative to V1^Foxp2^, V1^Sp8^, V1^Pou6f2^, and V1^Calb1^ clusters, which together constitute 95% of all V1 nuclei. **i** Dot plot showing the top 5 DEGs for each cluster, with corresponding nomenclature of each cluster at top. Names of key genes used to delineate different clusters are highlighted in red, and additional DEGs exhibiting specific expression but detected in a low proportion of nuclei (<10%) are highlighted in green. See also Supplementary Table 1. Source data are provided as a Source Data file.

Although the four primary V1 clades accounted for nearly 80% of all V1 interneurons in the snRNA-seq dataset, a substantial portion (clusters #1, #6, and #9) falls outside of those groups. Therefore, we sought to identify unique markers that would provide genetic access to the remaining V1 interneurons. Screening of differentially expressed genes identified *Rnf220*, an E3 ubiquitin ligase implicated in the specification of spinal progenitor domains and modulation of AMPA receptor-mediated synaptic transmission, as highly selective for clusters #1 and #6 (Fig. 3a)^44,45^. Multiplexed RNAScope against Rnf220 and the clade markers *Calb1, Foxp2, Pou6f2*, and *Sp8* showed each of these markers was expressed in V1 interneurons in a mutually exclusive manner, providing independent validation of our single nucleus transcriptomic analysis (Supplementary Fig. 4d–i). Moreover, immunohistochemical analysis confirmed the expression of Rnf220 in V1 interneurons (Fig. 3b), with V1^Rnf220^ neurons constituting ~13% of the overall V1 population and occupying a broad spatial distribution within the ventral spinal cord (Fig. 3c, d). Pairwise immunohistochemical analysis of Rnf220 with Foxp2, Pou6f2, Sp8, or MafA in lineage-traced V1 interneurons similarly revealed that V1^Rnf220^ neurons represent a largely non-overlapping population, entirely distinct from V1^Pou6f2^ and V1^MafA^ clades and with only slight overlap with V1^Foxp2^ and V1^Sp8^ clades (Fig. 3e–g). No single marker was expressed exclusively in the sole remaining cluster (#9); however, those neurons were most specifically characterized by expression of *Nr5a2* (Supplementary Fig. 5a and Supplementary Data 4). Based on the most differentially expressed genes (DEGs), we assigned a unique molecular nomenclature to each cluster to facilitate their identification across transcriptomic datasets (Fig. 3i; see also Supplementary Table 1.) Taken together, this data leads us to posit the existence of a fifth V1 clade, marked by the expression of *Rnf220*, and indicates that more than 95% of all V1 interneurons can be assigned to one of these five clades (Fig. 3h).

### Molecular profiles of V1 interneuron clades

Having established that five V1 clades can be distinguished by their transcriptomic profiles, we next sought to explore their overall taxonomy and molecular differences. Our prior classification schema based on limited TF expression implied that cell types within a clade are more similar than cell types across clades. Yet the opposite may be true, particularly when cells are assessed by their overall transcriptomic profile. Hierarchical clustering analysis revealed that certain clusters within a clade are more transcriptomically similar to each other than to clusters from other clades (e.g. clusters #5 and #7 within the V1^Sp8^ clade, or clusters #4 and #11 within the V1^Foxp2^ clade) (Supplementary Fig. 6a). This rule, however, was not absolute. For example, cluster #0 in the V1^Foxp2^ clade was most similar transcriptomically to cluster #10 in the V1^Sp8^ clade. Moreover, we identified highly distinct molecular profiles within a single clade, best exemplified in the V1^Pou6f2^ clade, where cluster #13 was far removed from clusters #12 and #3. The number of differentially expressed genes (DEGs) per cluster (Supplementary Fig. 6b and Supplementary Data 4), as well as Pearson correlation and distance in principal component space (Supplementary Fig. 6c, d), confirmed that cluster #13 is not only different from the other V1^Pou6f2^ clusters, but is the most unique amongst all V1 clusters. Thus, V1 subsets within an individual clade are often but not always closely aligned on the molecular level.

**Fig. 4:**
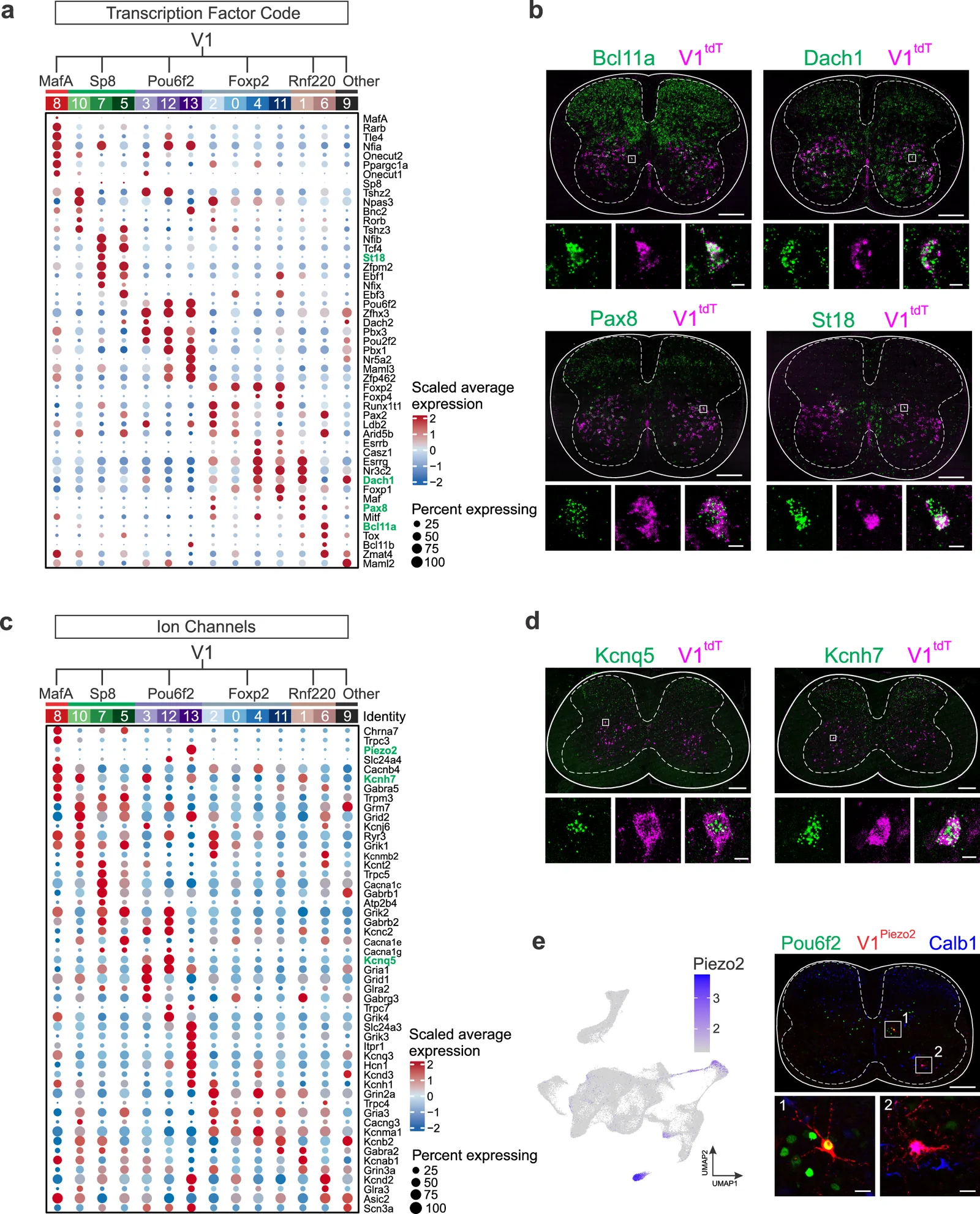
Molecular profiles of V1 interneuron subsets. **a** Average scaled expression of transcription factors (TFs) enriched in each of the 14 V1 clusters. TFs validated by in situ hybridization (ISH) appear in green text. **b** RNAScope ISH validating expression of select TFs (green) in V1 interneurons (magenta) in P0 lumbar spinal cords of *En1*^*Cre*^*; Ai14* mice. Scale bars = 200 μm (top) or 10 μm (bottom). **c** Average scaled expression of ion channels or transporters enriched in each of the 14 V1 clusters. Validated genes appear in green text. **d** RNAScope ISH validating expression of select ion channels in V1 interneurons in P28 lumbar spinal cord of *En1*^*Cre*^*; Ai14* mice. Scale bars = 200 μm (top) or 10 μm (bottom). **e** Left, UMAP showing *Piezo2* expression in V1 interneurons. Right, P0 cervical spinal cord from *Piezo2*^*Cre*^*; En1*^*Flpo*^*; Ai65D* mouse demonstrating Pou6f2 expression (green) or Calb1 expression (blue) in V1^Piezo2^ interneurons (red). Of the V1^Piezo2^ neurons in cervical segments, 41.1% ± 11% were Pou6f2^+^, 36.1% ± 4.5% were Calb1^+^, and 22.8% ± 6.6% were not positive for either marker, whereas at lumbar segments 2.8% ± 1.9% were Pou6f2^+^, 58.0% ± 2.1% were Calb1^+^, and 39.2% ± 2.0% were not positive for either marker (mean ± SEM, *n* = 3 animals). Scale bars = 200 μm (top) or 20 μm (bottom).

To better understand the unique molecular signatures of V1 clusters, we next focused on two broad categories of genes, TFs and ion channels, due to their important roles in shaping cellular identity and physiological properties. Analysis of the most differentially expressed TFs showed robust cluster-specific cohorts of TF genes, including many of the 19 TFs previously identified and validated as subdividing the larger V1 population^18^ (Fig. 4A and Supplementary Fig. 5a). For example, cluster #8, which corresponds to Renshaw cells, was defined not only by coincident expression of *Oc1*, *Oc2*, *MafA*, and *MafB* (Supplementary Fig. 5a), which is consistent with previous evidence^46^, but also by expression of *Tle4*, *Nfia*, *Rarb*, and *Ppargc1a*, all of which have been implicated in aspects of nervous system development^47–50^. In addition to *Prox1*, *Prdm8*, *Oc2*, and *St18*, the V1^Sp8^ clade was characterized by enriched expression of several TF families: Nfi-family members *Nfib* and *Nfix*, which control cellular differentiation in neurons and glia^51,52^; Ebf-family members *Ebf1* and *Ebf3*, which couple neuronal differentiation to cell-cycle exit in the spinal cord^53^; and Tshz-family members *Tshz2* and *Tshz3*, the latter of which is implicated in cortico-striatal synaptic transmission and autism-like behavior^54^. The V1^Pou6f2^ clade, previously shown to express Nr5a2, Lmo3, MafB, Oc1, and Oc2, was characterized by several additional TFs involved in neuronal specification: Pbx-family members *Pbx1* and *Pbx3*, which function as Hox cofactors and mediate segregation and clustering of spinal motor neurons within motor columns^55^; *Zfp462*, a Pbx1-interacting TF that safeguards neuronal lineage specification via epigenetic regulation of heterochromatin^56,57^; *Pou2f2*, which regulates the spatial organization of spinal V2a interneurons^58^; *Maml3*, a regulator of Notch signaling^59^, and *Zfhx3*, which is associated with long-range projection neurons in the spinal cord^40^. The largest V1 clade, V1^Foxp2^ interneurons, previously shown to express Foxp1/2/4, Otp, Nr4a2, Esrrb (Nr3b2), Esrrg (Nr3b3), and Lmo3 in varying combinations, was also enriched for *Pax2*^60^, *Casz1*, *Nr3c2*, and *Dach1*. Finally, V1^Rnf220^ interneurons were characterized by enriched expression of *Pax8*, previously suggested to participate in dorsal inhibitory interneuron specification^61^, Bcl11-family members *Bcl11a* and *Bcl11b* (cluster #6), and *Esrrg*, *Nr3c2*, *Maf*, and *Dach1* (cluster #1). RNAScope analysis of select TFs, including *Pax8*, *Bcl11a*, *Dach1*, and *St18*, independently validated their expression in V1 interneurons (Fig. 4b). Together with earlier immunohistochemical validation of many of these differentially expressed V1 TFs^18^, this analysis provides insight into the transcriptional programs differentiating subsets of V1 interneurons.

We next examined the differential expression of ion channels and transporters amongst subgroups of V1 interneurons, which have distinct electrophysiological signatures^18^. Members of the voltage-gated K^+^ channel family were particularly enriched, with 13 of the top DEGs belonging to this family (Fig. 4c). Ionotropic glutamate receptors (10 of the top DEGs), Trp channels (5 of the top DEGs) and voltage-gated Ca^2+^ channels (5 of the top DEGs) also constituted a substantial portion of identified ion channels. RNAScope in situ hybridization (ISH) of select differentially expressed KCN-family members that regulate neuronal excitability (e.g. *Kcnq5*^62^ and *Kcnh7*^63^) confirmed the expression of these genes in V1 interneurons (Fig. 4d). Interestingly, we found that *Piezo2*, the principal mechanoreceptor mediating light touch and proprioception^64,65^, was among the most highly selectively expressed ion channels, with particularly strong expression in cluster #13, a subset of the V1^Pou6f2^ clade, and weak expression in cluster #8, representing the MafA/Calb1 clade (Fig. 4c, e). To determine if *Piezo2* is indeed expressed in subsets of V1 interneurons, we generated *Piezo2*^*Cre*^*; En1*^*Flpo*^*; Ai65D* mice to lineage-trace V1^Piezo2^ interneurons. We identified tdTomato-expressing neurons that were positive for *Pou6f2* expression and *Calb1* expression in cervical spinal cords of P0 mice, thus validating our transcriptomic data and suggesting these neurons may be mechanosensitive (Fig. 4e). Collectively, our snRNA-seq data reveals that in addition to their unique TF profiles, V1 clades express distinct cohorts of ion channels that may in part reflect differences in their electrophysiological properties.

### Changes in gene expression across postnatal development do not alter the core identity of V1 interneuron clades

Following their birth, spinal neurons undergo a protracted period of maturation that extends from embryogenesis well into adulthood^66^. Embryonic V1 interneurons segregate into distinct cell types shortly after neurogenesis^29^, consistent with observations that V1 clades are born in sequential but partly overlapping waves from E9.5 to E13.5^28,46,67^. By P0, they exhibit even more extensive molecular diversity^19^. Yet it remains unclear how this developmental diversity relates to mature neural circuits in the spinal cord. Neonatal V1 diversity may represent a transient stage in the development of motor circuits, analogous to how some *Drosophila* olfactory projection neurons exhibit peak diversity during circuit assembly but then converge to largely indistinguishable cell types in adults^68^. This possibility is suggested by the observation that V1 interneurons (and ventral interneurons more generally) in the adult spinal cord exhibit less pronounced distinctions in cell type identity than do their dorsal interneuron counterparts^32,33,69^. Alternately, such diversity may be maintained in mature circuits, analogous to interneurons in the adult cortex^70^.

**Fig. 5:**
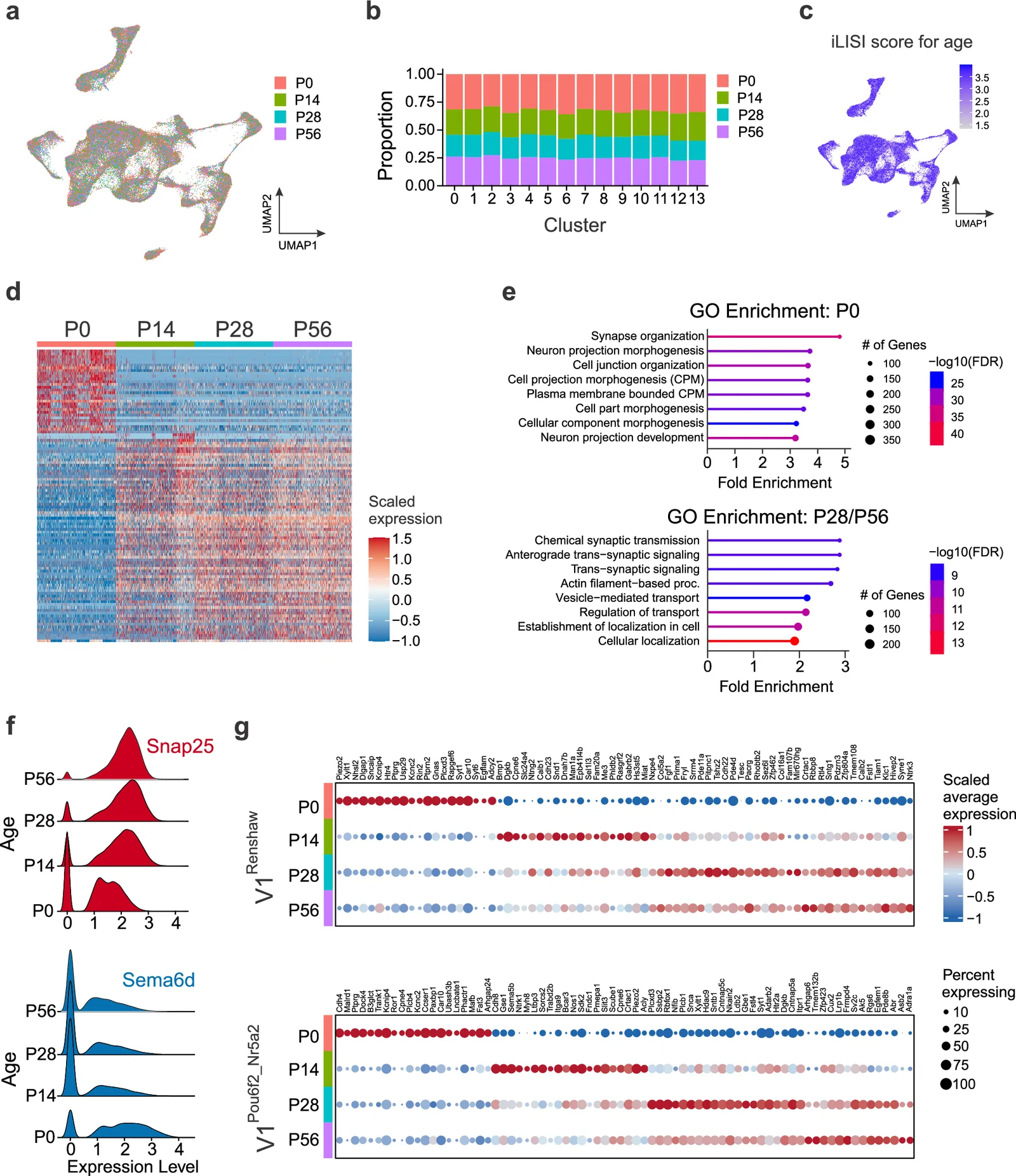
Changes in gene expression across postnatal development do not change core neuronal identity. **a** UMAP of V1 nuclei color-coded by age (P0, P14, P28, or P56) at the time of sample collection. **b** The relative contribution of each sample to the 14 V1 clusters. **c** Integrated local inverse Simpson’s index (iLISI) calculated for age. iLISI scores ranged from 1 to 4 (the number of groups), with higher scores indicating better intermixing between groups. **d** Heatmap of the top 30 most differentially expressed genes at each time point. **e** Gene ontology (GO) analysis showing the top 8 biological processes enriched in the gene expression profiles of V1 nuclei at P0 (top) or P28 and P56 combined (bottom). **f** Ridge plot highlighting genes with increasing expression (*Snap25*, red) and decreasing expression (*Sema6d*, blue) from P0 to P56. Log normalized values are shown. **g** Identification of genes dynamically regulated across postnatal development that are specific to V1^Renshaw^ (cluster #8, top) or V1^Pou6f2_Nr5a2^ (Cluster #13, bottom).

To explore whether V1 interneurons retain their transcriptomic identity, we assessed the extent to which V1 interneurons from different postnatal ages contribute to each of the clusters. V1 interneurons from P0, P14, P28, and P56 mice were found in each cluster, in roughly equal proportions (Fig. 5a, b), indicating that new V1 subtypes do not appear with age, nor does the initial diversity present at birth decrease with age. Consistent with this finding, quantification of the contribution of V1 nuclei to each cluster, as a function of age by using integrated local inverse Simpson’s index (iLISI)^71^, showed a high degree of intermixing (Fig. 5c). Together, these data suggest that the core identity of V1 interneurons in neonatal mice is preserved through adulthood.

Despite the overall consistency in V1 cell-type identity, we observed substantial age-related changes in gene expression (Fig. 5d and Supplementary Data 5). The largest changes occurred during the first 2 postnatal weeks, a period characterized by rapid maturation of synaptic connectivity, myelination, and the development of intrinsic cell properties^72^. In contrast, changes were less pronounced from P14 onward. Gene ontology (GO) analysis of the most differentially expressed genes at P0 identified enrichment of those involved in synapse organization and neuron projection morphogenesis/development, whereas at P28 and P56 DEGs were associated with synaptic transmission and transsynaptic signaling (Fig. 5e). Consistent with these cellular processes, we identified decreasing developmental expression of *Sema6d*, which is involved in guiding axons to their appropriate targets^73^, and increasing expression of *Snap25*, a key component of synaptic vesicle exocytosis machinery (Fig. 5f)^74^. Beyond global changes in V1 interneuron transcriptomic profiles across development, we identified cluster-specific changes in gene expression, as exemplified in V1^Renshaw^ neurons (cluster #8), where *Piezo2* expression was highest at P0 and downregulated by P14 onward, whereas in V1^Pou6f2_Nr5a2^ neurons (cluster #13), *Piezo2* expression had the opposite dynamic, showing increased expression from P0 to P56 (Fig. 5g). Thus, V1 interneurons exhibit clear developmental changes in gene expression during postnatal maturation yet maintain stability of their general cell-type identity.

### Loss of a specific subset of V1 interneurons in *En1* KO mice

**Fig. 6:**
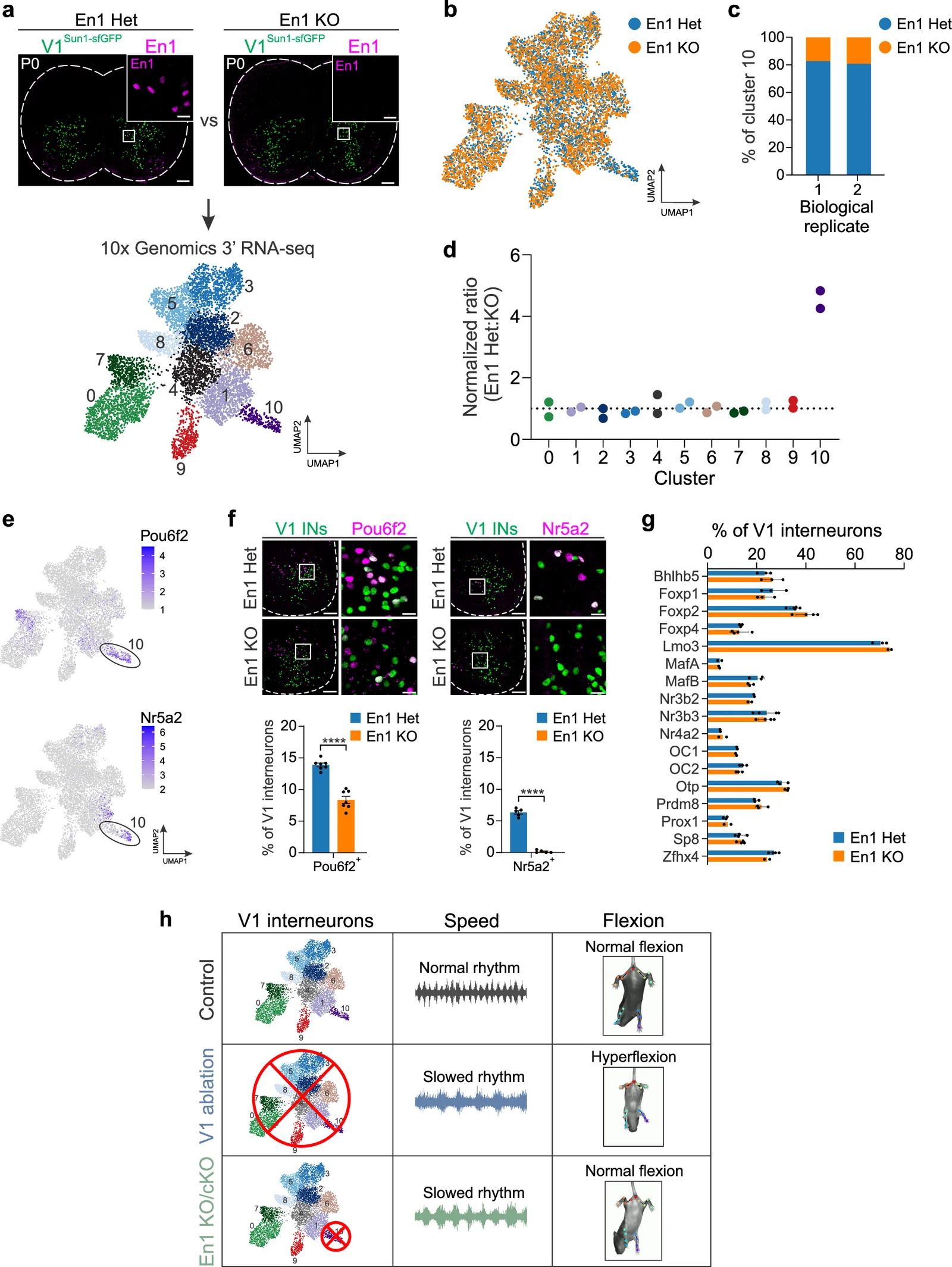
En1 KO mice lack a specific subset of V1 interneurons within the V1^Pou6f2^ clade. **a** Top, representative images of P0 lumbar spinal cords from *En1*^*Cre*^ heterozygous; *RC.lsl.Sun1-sfGFP* (En1 Het) or *En1*^*Cre*^ homozygous; *RC.lsl.Sun1-sfGFP (*En1 KO) mice immunostained for En1 (magenta) and lineage-traced V1 interneurons (green). Bottom, UMAP visualization of unsupervised clustering of snRNA-seq data generated for these genotypes, identifying 11 distinct clusters. **b** UMAP of *En1*^*Het*^ (blue) and *En1*^*KO*^ (orange) nuclei. **c** Normalized percentage of nuclei in cluster 10 originating from either *En1*^*Het*^ or *En1*^*KO*^ animals across two biological replicates. **d** Normalized ratio of Het:KO nuclei in each cluster across two biological replicates, where 1 corresponds to equal representation of both genotypes. With cluster #2 as a reference, only cluster #10 was found to have a credible change in abundance, as determined by scCODA (log2FC = −1.61, FDR < 0.05). **e** UMAP showing expression of *Pou6f2* and *Nr5a2* enriched in Cluster #10. **f** IHC analysis of V1^Pou6f2^ (left) and V1^Nr5a2^ (right) interneurons in P0 lumbar spinal cords of *En1*^*Het*^ or *En1*^*KO*^ mice demonstrating a significant reduction in each subset. V1 interneurons were lineage-traced using *Tau.lsl.nLacZ* mice (green). V1^Pou6f2^: 13.9% ± 0.3% vs 8.4% ± 0.6% in Het vs KO animals, respectively, mean ± SEM, *n* = 7 animals; V1^Nr5a2^: 6.3% ± 0.3% vs 0.1% ± 0.1% in Het vs KO animals, mean ± SEM, *n* = 5 animals, **** *p* < 0.0001, unpaired two-tailed *T*-test). **g** Percentage of V1 subsets defined by 17 other TFs in *En1*^*Het*^ or *En1*^*KO*^ mice assessed by IHC. No statistical difference between genotypes was observed (mean ± SEM, *p* > 0.5 for all comparisons containing *n* = 3, 4, or 5 mice, unpaired two-tailed *t*-test with Holm-Sidak correction for multiple comparisons). For conditions where *n* = 2, only mean is reported. **h** Summary illustrating the transcriptomic heterogeneity and functional effects of V1 interneurons on rhythmic locomotor output and limb flexion. See Supplementary Table 2 for statistics of all comparisons. Source data are provided as a Source Data file.

Having generated a detailed transcriptomic atlas of V1 diversity, we sought to leverage this knowledge to explore how such diversity might underlie the multifunctional role of V1 interneurons in motor output. Given that only a subset of motor deficits was observed in En1 knockout mice (i.e. slowed locomotor rhythm but not limb hyperflexion), we hypothesized that En1 may be required for the development of specific types of V1 interneurons, presumably those involved in regulating rhythmic locomotor speed. Consistent with prior observations that mice lacking En1 protein do not show obvious deficits in V1 interneuron fate specification^75,76^, we found that the overall number of V1 interneurons was comparable in *En1*^*Het*^ and *En1*^*KO*^ animals (268 ± 9 neurons and 265 ± 7 neurons per 20 μm section, respectively; mean ± SEM, *n* = 5 mice, *p* = 0.815, unpaired two-tailed t-test) (Supplementary Fig. 7a). We therefore turned to a transcriptomic analysis of V1 interneurons lacking En1. Following isolation of V1 nuclei from the spinal cords of P0 *En1*^*Het*^ and *En1*^*KO*^ mice, we performed snRNA-seq and isolated 7553 nuclei (replicate 1) or 7418 nuclei (replicate 2) that passed V1 selection criteria, revealing 11 distinct clusters (Fig. 6a). The molecular identity of these clusters aligned well with our reference postnatal V1 dataset (Supplementary Data 6; see also Supplementary Table 1 for molecular nomenclature), though we identified fewer clusters, likely reflecting limitations in resolving more subtle gene expression differences given the ~10-fold lower sampling of nuclei. Segregation of the data by genotype revealed one cluster (#10) with a paucity of *En1*^*KO*^ nuclei (Fig. 6b). Quantification revealed a striking reduction (~2.5-fold decrease) of *En1*^*KO*^ nuclei in cluster #10 in two independent biological replicates (Fig. 6c). In contrast, the proportion of *En1*^Het^:*En1*^*KO*^ nuclei within each of the other 10 clusters was nearly equivalent, indicating the selective paucity of neurons in cluster #10 (Fig. 6d). Moreover, our analysis of TF expression in *En1*^*Het*^ and *En1*^*KO*^ mice showed no substantial differences in gene expression, indicating cell-type identity of the remaining V1 interneurons in *En1*^*KO*^ mice is not significantly perturbed (Supplementary Fig. 7b).

An analysis of V1 clade markers revealed that cluster #10 corresponded to a subset of the V1^Pou6f2^ clade that also expresses Nr5a2, suggesting there may be a substantially smaller proportion of these neurons in *En1*^*KO*^ mice (Fig. 6e). Independent immunohistochemical analysis of P0 lumbar spinal cord confirmed a significant reduction in the percentage of Pou6f2^+^ and Nr5a2^+^ V1 interneurons in *En1*^*KO*^ mice (Fig. 6f). A similar reduction in V1^Pou6f2^ interneuron number was also observed in *En1 cKO* animals (Supplementary Fig. 7c, d). Moreover, pseudo-bulk analysis of gene expression comparing *En1*^*Het*^ and *En1*^*KO*^ nuclei identified *Nr5a2* and *Pou6f2* as the two most significantly down-regulated genes in *En1*^*KO*^ nuclei (Supplementary Fig. 7e). In comparison, immunohistochemical analysis of the proportion of V1 subsets defined by expression of 17 other TFs showed no significant difference between *En1*^*Het*^ and *En1*^*KO*^ animals (Fig. 6g). Consistent with these observations, analysis of the spatial location of V1 interneurons in lumbar spinal segments of control and *En1*^*KO*^ mice revealed that the position of V1^Pou6f2^ neurons was selectively perturbed in the absence of *En1*, whereas the distribution of other V1 populations was unaffected (Supplementary Fig. 7f). Thus, En1 plays a critical role in the development of a subset of V1^Pou6f2^ interneurons, revealing a highly specific disruption in V1 interneuron identity associated with slowed rhythmic locomotor activity.

## Discussion

How diverse sets of interneurons in the spinal motor system contribute to discrete elements of movement remains a major unresolved question. Here, we show that two functional roles attributed to the cardinal class of V1 interneurons – the control of rhythmic locomotor speed and flexion of limbs - can be segregated from one another. Through a deep transcriptomic analysis of the V1 population, we define the landscape of V1 diversity and find that *En1*^*KO*^ mice exhibiting slowed locomotor rhythm have a highly selective loss of a single type of V1 interneuron (Fig. 6h). These studies provide insight into the multifunctional role of cardinal interneuron populations in movement and illustrate how deep neuronal profiling can begin to bridge the gap between neuronal diversity and motor function.

### V1 transcriptomics as a bridge to understanding motor function

A hallmark of motor output is its highly adaptable nature, enabling animals to tailor their limb movements to different behavioral demands. In the context of locomotion, variations in speed and gait play a critical role in activities such as exploration, which involves slower rhythmic output accompanying walking and trotting, and escape behaviors like galloping that necessitate faster speeds^77–79^. The spinal cord relies on neural circuits comprised of multiple interneuron populations, including V0, V1, V2, V3, and dI6 classes, to facilitate specific elements of movement such as left/right alternation (V0, V2a, and dI6)^10,12,13,80,81^, the speed of rhythmic locomotor activity (V1 and V2b)^11,82^, robustness of locomotor rhythm (V3)^14^, and flexion/extension (V1 and V2b)^15,20^. Emerging evidence indicates that such circuits are organized in a modular nature, with different subsets of interneurons and motor neurons implementing distinct components of movement^77,83–85^. Because each interneuron population is highly heterogeneous^17^, unraveling the contributions of specific types of interneurons to motor output requires a careful delineation of neuronal diversity.

Single-cell transcriptomic approaches have greatly facilitated the systematic characterization of cell types across the nervous system^34,86^. In the mouse spinal cord, numerous transcriptomic studies have characterized the larger cellular ecosystem of neuronal and non-neuronal populations in the developing and mature spinal cord^29–34,40^. These global surveys have been complemented by targeted studies of spinocerebellar neurons^87^, V2a interneurons^42^, and perhaps most prominently, motor neurons^88–90^. Yet the diversity within individual classes of spinal interneurons has remained obscure, limiting our ability to dissect the synaptic and circuit architecture of spinal circuits and their contributions to behavior. Our finding that the absence of *En1* results in a pronounced slowing of rhythmic locomotor output while sparing flexion/extension, in contrast to the disruption of both aspects of movement upon ablation or silencing of V1 interneurons^11,15,20^, highlights the need for investigating the diversity of spinal interneuron classes to understand motor output. In this case, transcriptomic analysis of the V1 population revealed that the V1^Pou6f2_Nr5a2^ subset is the sole cluster substantially impacted in *En1*^*KO*^ mice, implicating it in the control of locomotor speed. In this scenario, the control of limb flexion would primarily be mediated by a different V1 subset, possibly the V1^Foxp2^ clade, which contains neurons with the classic anatomical features of Ia reciprocal inhibitory interneurons^28^ and appears unaffected by loss of *En1*. Indeed, we observed no effect on the speed of rhythmic locomotor-like output upon ablation of V1^Foxp2^ interneurons, consistent with a primary role of V1^Foxp2^ interneurons in other aspects of motor output. We were unable to determine whether V1^Foxp2^ neurons are directly responsible for controlling limb flexion, due to our inability to restrict En1^+^/Foxp2^+^ neuronal ablation to the spinal cord and associated neonatal lethality following DT-administration to *En1*^*Cre*^*; Foxp2*^*Flp*^*; Tau.ds.DTR* mice. Future studies will therefore be required to determine whether V1^Foxp2^ neurons are directly responsible for controlling limb flexion. Interestingly, monosynaptic rabies virus tracing from cervical V1^Pou6f2^ and V1^Foxp2^ interneurons revealed biased inputs from the medullary gigantocellular reticular nucleus to V1^Pou6f2^ interneurons and vestibular nuclei to V1^Foxp2^ interneurons, providing a potential means through which analogous V1 subsets in the lumbar spinal cord may be differentially recruited by descending motor commands involved in locomotion and balance^91–95^.

While the mechanisms through which the absence of En1 and the accompanying loss of V1^Pou6f2_Nr5a2^ interneurons might control the speed of locomotor rhythm are unclear, V1 interneurons are known to provide in-phase inhibition to other neurons during locomotion^96^. In this manner, they may help terminate an ongoing cycle, thus shortening the cycle period to facilitate faster speeds. Alternatively, V1 interneurons have been proposed to be part of a disinhibitory circuit in which their loss leads to increased tonic inhibition and slowing of flexor-associated rhythmic activity^97^. Defining the circuit architecture of V1^Pou6f2^ interneurons, including their postsynaptic targets, will be critical to elucidate their role in locomotor output. Additionally, developmental deficits in axon fasciculation and synaptic connectivity have been observed in *En1*^KO^ mice and may contribute to the altered rhythmic locomotor speed observed in these animals^76,98^. We note that we have not yet been able to target V1^Pou6f2_Nr5a2^ interneurons to directly demonstrate their involvement in the control of locomotor speed, in part due to inefficiency of recombination in our *Pou6f2*^*Flpo*^ line, which prevents us from effectively targeting V1^Pou6f2^ neurons for functional perturbation experiments. Future studies will be required to define the relative contributions of V1^Pou6f2/Nr5a2^ neurons and disruptions in V1 connectivity to shaping locomotor speed, as well as to clarify the role of other V1 subsets described in our transcriptomic dataset in controlling movement.

### Involvement of class-defining transcription factors in cell-type specification and behavior

During development, spatially and transcriptionally distinct progenitor domains oriented along the dorsoventral spinal axis give rise to cardinal classes of interneurons whose postmitotic expression of key TFs defines their identity^18,99^. Many of these TFs play a crucial role in cell-type specification of their respective interneuron classes. Thus, *Evx1* mutant mice exhibit abnormal V0_V_ interneuron development in which V0_V_ interneurons adopt a V1-like fate^100^, and *Chx10* mutants show substantially reduced numbers of V2a interneurons^101^. Yet such disruptions do not inevitably lead to obvious perturbations in locomotor output, exemplified by *Evx1* mutant mice that show normal left/right and flexor/extensor-associated motor activity^12^. In other cases, cell fate specification itself appears largely preserved. For example, *Sim1* mutant mice produce normal numbers of V3 interneurons, though remaining neurons exhibit abnormal patterns of migration and axon pathfinding^102^.

Prior studies on *En1* mutant mice suggested that overall V1 cell fate specification is preserved, with neurons exhibiting axon pathfinding deficits and reduced Renshaw cell innervation of motor neurons^76,98^. Our finding that *En1* is required for the normal development of V1^Pou6f2_Nr5a2^ interneurons illustrates the power of unbiased transcriptomic approaches to reveal highly specific deficits in interneuron specification that may shape motor output. The observation that affected neurons constitute only a tiny fraction (~3%) of the overall V1 population likely explains why prior studies did not identify changes in V1 cell fate. This is different from the role *En1* plays in midbrain dopaminergic neurons, where *En1* and the related family member *En2* are essential for dopaminergic neuron survival^103^. We also did not observe significant increases in other V1 clusters in *En1*^*KO*^ animals, though this might be expected even if V1^Pou6f2/Nr5a2^ neurons adopted an altered cell fate, given their sparsity within the V1 population. Thus, it remains unclear whether affected V1^Pou6f2_Nr5a2^ interneurons are never born, are born but prematurely die, or ultimately adopt an alternate V1 cell fate. Analysis of the gene regulatory networks controlled by *En1* should help clarify the underlying mechanisms through which V1^Pou6f2_Nr5a2^ identity is specified.

### V1 interneuron diversity in context

Given the fundamental role of spinal circuits in sensory perception and motor output, considerable effort has gone into finding an overarching logic to the classification of spinal neurons. From a developmental perspective, neuronal diversity in the spinal cord emerges through the sequential generation of neurons from distinct embryonic progenitor domains arrayed along the dorsoventral and anteroposterior spinal axes^104^. These three principles - genetic provenance, spatial organization, and temporal specification - have provided a complementary framework to classical physiological studies to gain a better understanding of the diversity of neurons in the spinal cord. Layered onto these classification schemes, recent transcriptomic studies have revealed that dorsal versus ventral identity contributes the most variability to gene expression^40^, and these two general categories fundamentally differ - dorsal interneurons exhibit clear transcriptomic distinctions, and ventral interneurons show more overlapping profiles^32,69,105^.

Our atlas of more than 89,000 V1 interneuron nuclei, spanning development from birth to adulthood, enables an exploration of the organizing principles of V1 diversity and its relation to the larger cellular ecosystem in the spinal cord. V1 interneurons comprise a highly diverse set organized into at least four mutually exclusive clades, V1^Foxp2^, V1^MafA^, V1^Pou6f2^, and V1^Sp8^, that are collectively composed of dozens of putative cell types that can be distinguished based on TF expression, cell body position, and intrinsic physiological properties^18,19^. Strikingly, this clade organization appears to be largely conserved across 300 million years of evolution, where V1 interneurons in *Xenopus* express Foxp2, MafA, Pou6f2, and Sp8 TFs in a manner that mirrors the mutually exclusive expression observed in mice^23^. Here we show that these four established clades and the newly identified V1^Rnf220^ clade represent distinct cell types based on unbiased transcriptomic clustering. The fractionation of V1 interneurons into 14 distinct clusters represents a resolution that identifies robust differences in gene expression and shows excellent alignment with our previous analysis of V1 clade diversity, albeit with substantially fewer total numbers of predicted cell types^19^. Interestingly, while some clusters within a given V1 clade were quite similar, others within that same clade were highly transcriptomically divergent (e.g. V1^Pou6f2^ cluster #13 vs. clusters #3 and #12). Given that transcriptomic distinctions in cell type identity correspond with other phenotypic modalities, including anatomical and physiological properties^106,107^, it is reasonable to expect that individual V1 clades contain interneurons with disparate connectivity profiles and roles in motor output.

Beyond this, several core insights into the likely phenotypic characteristics of V1 interneurons emerge from our transcriptomic analysis. Previous studies identified a major axis of gene expression in ventral interneurons that was predictive of mediolateral position, birthdate, and projection patterns, where Group-N (NeuroD2 and Nfib-expressing) neurons are medial, late-born, and locally projecting, and Group-Z (Zfhx3, Zfhx4, and Foxp2-expressing) neurons are lateral, early-born, and long-distance projecting^40^. We find that at a low level of clustering, V1 interneurons divide into Group-N neurons represented by a subset of the V1^Sp8^ clade, and Group-Z neurons comprising all other clades. This general logic aligns well with prior positional analysis of V1 interneurons^18,19,40^ and birth-dating studies showing that V1 clades emerge sequentially in overlapping waves between E10 and E12.5, with V1^MafA/Renshaw^ and V1^Pou6f2^ neurons born early, followed by V1^Foxp2^ neurons, and ending with V1^Sp8^ neurons^28^. It is somewhat less clear how the logic of Group-N and Group-Z projection patterns applies to V1 interneurons, as both V1^Foxp2^ and V1^MafA/Renshaw^ neurons provide dense, local innervation of motor neurons, despite their Group-Z (long-distance projecting) designation^28^.

Our transcriptomic analysis of V1 interneurons from P0 to P56 also provides insight into how cell-type identity changes during postnatal maturation. The largest transcriptional changes occur during the first 2 postnatal weeks, which correspond to a rapid maturation in motor behavior that culminates in adult-like locomotion by the third week^108^. Thereafter, even as neural circuit refinement continues, the molecular profiles of V1 interneurons stabilize. This is reminiscent of motor neuron gene expression programs that are highly dynamic from embryogenesis through P21, at which point transcriptional and chromatin-accessibility landscapes stabilize^66^. Importantly, our integrated dataset revealed a near equal contribution of all sample ages to each cluster, indicating that the extensive cellular diversity documented within V1 interneurons in the neonatal spinal cord is largely preserved through adulthood; V1 interneuron clusters retained their core identity, while undergoing temporal changes in gene expression. Our ability to resolve differences in adult V1 interneurons may in part reflect the large number of V1 nuclei sampled in our dataset, analogous to findings that defined extensive diversity within adult motor neurons following targeted enrichment of that neuronal population^89^. Notably, several transcriptomic studies have found that cardinal classes of ventral interneuron populations are not easily distinguishable, such that V1, V2b, and other inhibitory cardinal classes merge together^32,69^. It is possible that even as V1 interneurons retain substantial transcriptional diversity, there is a convergence of gene expression patterns for cell types in different interneuron classes that exhibit similar anatomical or functional characteristics. Indeed, functionally and anatomically defined types of spinal interneurons may span multiple cardinal populations, exemplified by Group Ia reciprocal interneurons that are found in both the V1 and V2b populations, or propriospinal neurons that originate from dI3, V1, V2, and V3 populations^15,109^. The continued use of transcriptomics and circuit-level dissection of spinal interneurons promises to provide substantial insight into the development and function of discrete cell types within the larger cellular ecosystem of the spinal cord.

## Methods

### Animals

All experiments and procedures were performed in accordance with National Institutes of Health (NIH) guidelines and approved by the Institutional Animal Care and Use Committee (IACUC) of St. Jude Children’s Research Hospital. Mice were housed on ventilated racks in a temperature and humidity-controlled room on a standard 12-hour light/dark cycle with *ad libitum* food and water. Mice of both sexes were used for experiments. The following previously published mouse lines were used in this study: C57BL/6 J (Jackson Laboratory #000664), *RC.lsl.tdT* (*Ai14*) (Jackson Laboratory #007914), *RC.fsf.tdT* (*Ai65F*) (Jackson Laboratory #032864), *RC.dual.tdT* (*Ai65D*) (Jackson Laboratory #021875), *RC.lsl.ntdT* (*Ai75*) (Jackson Laboratory #025106), *En1*^*Cre*^ (Jackson Laboratory #007916), *En1*^*Flpo*^^110^*, En1*^*flox*^ (Jackson Laboratory #007918), *Foxp2*^*Flpo*^^18^*, Hoxb8*^*Cre*^^26^*, Piezo2*^*Cre*^ (Jackson Laboratory #027719), *RC.lsl.Sun1-sfGFP* (INTACT) (Jackson Laboratory #021039), *Tau.lsl.nLacZ* (Jackson Laboratory #021162), and *Tau.ds.DTR*^20^.

### Tissue collection and nuclei preparation

Spinal cord tissue was isolated from cervical, thoracic, and lumbar regions of *En1*^*Cre*^*; RC.lsl.Sun1-sfGFP* double-heterozygous mice at P0, P14, P28, or P56 (for the developmental snRNA-seq atlas) or from the whole spinal cord (for P0 *En1*^*Het*^ and *En1*^*KO*^ experiments and the E13.5 reference sample), pooling 4–12 animals per experiment. Two independent biological replicates were performed per condition. Animals were anesthetized either on ice (P0 pups) or via intraperitoneal injection of Avertin (Tribromoethanol, 240 mg/kg body weight for P14, P28, and P56 animals). Transcardial perfusion was performed with ice cold, oxygenated artificial cerebral spinal fluid (ACSF) consisting of 125 mM NaCl (Sigma #S9888), 2.5 mM KCl (Sigma #9333), 1.25 mM NaH_2_PO_4_∙H_2_O (Sigma #S71507), 26 mM NaHCO_3_ (Sigma #S5761), 25 mM glucose (Sigma #G7021), 5 mM ethyl pyruvate (Sigma #E47808), and 0.4 mM sodium ascorbate (Sigma #11140), supplemented after oxygenation with 1 mM MgCl_2_ (Sigma #M2392), 2 mM CaCl_2_ (Sigma #C5670), and blockers of neuronal activity and transcription (20 μM AP5 (Tocris #1060), 20 μM DNQX (Tocris #018910), 0.1 μM TTX (Tocris #1069), 5 μM Actinomycin D (Sigma #A1410)). Spinal cords were quickly extruded, separated into cervical, thoracic, and lumbar regions, then flash frozen and stored in liquid nitrogen until dissociation.

Nuclei dissociation was performed as described in Matson et al.^111^ Briefly, frozen tissue was mechanically dissociated with a Dounce homogenizer (Kontes Dounce Tissue Grinder) in 500 μL sucrose buffer (0.32 M sucrose (Sigma #84097), 10 mM HEPES pH 8.0 (Gibco #15630), 5 mM CaCl_2_ (Sigma #C5670), 3 mM magnesium acetate (Sigma #63052), 0.1 mM EDTA (Invitrogen #AM9261), 1 mM DTT (Sigma #10197777001) with 0.1% Triton-X100 (Sigma #T8787), using five strokes of the A pestle followed by 15 strokes with the B pestle or until the tissue was dissociated. The resulting homogenate was filtered through a 40 μm strainer and washed with 3 mL of sucrose buffer. Nuclei were then centrifuged at 3200 × *g* for 10 min at 4 °C. The supernatant was decanted, and nuclei were resuspended in 3 mL of sucrose buffer and transferred to an Oak Ridge centrifuge tube. A high-density sucrose solution containing 1 M sucrose, 10 mM HEPES pH 8.0, 3 mM magnesium acetate, and 1 mM DTT was carefully layered underneath the remaining supernatant and then spun at 3200 x *g* for 20 min at 4 °C. The supernatant was discarded, and the remaining nuclei were resuspended in 500 μL of phosphate-buffered saline (PBS) with 0.4 mg/mL BSA (NEB #30281) and 0.2 U/μL RNAse inhibitor (Lucigen #30281-1) for subsequent processing by flow cytometry.

### Flow cytometry

Fluorescence-activated nuclei sorting (FANS) of sfGFP^+^ nuclei was performed using a FACSAria Fusion Cell Sorter (BD Biosciences, San Jose, CA). The FACSAria Fusion is equipped with a 50 mW 488 nm laser and a 530/30 bandpass filter and uses BD FACSDiva software v8.0.1. GFP^+^ nuclei were selected based on log side scatter (SSC-A) versus log GFP fluorescence (GFP-A) and sorted in purity mode (sheath pressure 21 PSI, 100 um nozzle) into 1.5 mL Protein LoBind Eppendorf tubes coated overnight with PBS + 2% BSA. Samples were visually inspected and counted on a hemocytometer after processing by flow cytometry.

### snRNA-seq library preparation and sequencing

Sorted GFP^+^ nuclei were loaded onto the 10x Genomics Chromium Controller (#1000171) to generate single-nucleus gel beads-in-emulsions (GEMs) using the Chromium Next GEM Single Cell 3’ GEM, Library, and Gel Bead Kit v3.1 (#1000121) and Chromium Single Cell B Chip Kit (#1000120), according to the manufacturer’s instructions. Approximately 8,000 nuclei were loaded for capture per sample. Following capture and lysis, cDNA synthesis, amplification, and library construction were performed on an ABI ProFlex PCR System (Applied Biosystems). The libraries were quantified using the Quant-iT Pico Green dsDNA assay (Thermo Fisher) and through low-pass sequencing with a Miseq nano kit (Illumina). Quality assessments were conducted using the 4200 TapeStation D1000 and D5000 ScreenTape assay (Agilent). Paired-end 100-cycle sequencing (Run configuration: 101-8-8-101) was carried out on a NovaSeq 6000 (Illumina) to a depth of at least 30,000 reads per nucleus in the St. Jude Hartwell Center for Biotechnology.

### snRNA-seq analysis

#### Data preprocessing and quality control

Sequencing data were aligned to the mouse genome (mm10, 10x Genomics version 2020 A) using CellRanger (v7.0.0) with default parameters except for the --force-cells option, which was set to 8000 for all datasets based on the estimated number of nuclei loaded by manual counting after sorting. The E13.5 dataset used for positive selection was mapped using CellRanger v3.0.0. Ambient RNA was removed from the samples using SoupX^112^ (version 1.6.2). Of the 24 samples used for the atlas, one (P56LR1) was removed at this stage due to high ambient RNA identified by SoupX. The gene expression matrix was then analyzed in R (v4.1.2) using the Seurat package (v4.3.0). See https://github.com/Bikoff-Lab for the complete list of libraries and version numbers used for downstream data processing. Each dataset underwent individual quality control (QC) to remove low-quality data and doublets. Briefly, nuclei were filtered out if they contained <1000 unique molecular identifiers (UMIs), >2.5% of transcripts derived from mitochondrial genes, or were above or below ±3 median absolute deviations (MADs) from the median number of UMIs and genes per sample. Any gene that appeared in <10 cells was removed.

#### Identification of V1 nuclei

To generate the V1 interneuron atlas, nuclei that passed QC underwent an iterative process of negative and positive selection. This enabled us to overcome two key challenges: (1) excluding any contaminating non-V1 nuclei that remained in the post-FANS sample, and (2) ensuring that we could identify V1 nuclei, given that levels of mRNA expression of GFP and En1 (a marker of V1 identity) were sufficiently low that they could not be used as a reliable indicator of the V1 lineage. Initial rounds of negative selection were performed first on a merged Seurat object, followed by negative selection on an integrated dataset with batch correction. We used a combination of QC metrics and known marker genes to remove non-V1 nuclei. Positive selection was performed using a previously annotated dataset generated by Delile et al.^29^ and the label transfer method in Seurat. A brief summary of this process is outlined below.

*Negative selection*: Initial rounds of negative selection on a simple merge of all samples first underwent standard log normalization per 10,000 reads followed by scaling of all genes. The top 2000 genes with the most variable expression across the entire merged dataset using the vst method and then linear dimensionality reduction with PCA was performed. To determine how many principal components to use in downstream analysis, two criteria were used: (1) the cumulative percent variation explained was >90% and the individual percent variation explained was <5%; (2) the change in percent variation was >0.1% (custom function called find_PC). Further dimensionality reduction and clustering were performed in accordance with the standard Seurat workflow (FindNeighbors, FindClusters, and RunUMAP) using default parameters. To annotate the resulting clusters and eliminate non-V1 nuclei, unhealthy/dying cells and empty GEMs were identified by using a combination of the total number of counts, total number of unique genes detected, percent mitochondrial reads, and the number of DEGs. A series of marker genes was used to identify non-neuronal clusters and defined types of neurons, based on previous studies^29,32,33,40,105^. (See Supplementary Data 2 for a list of marker genes used to identify these cell types). After removing non-V1 nuclei, the negative selection process was repeated three times until no further contamination was identified. The resulting data were subjected to further negative selection by using a similar approach but with batch correction using Seurat’s anchor-based Canonical Correlation Analysis (CCA) integration method. Standard normalization and scaling of the counts was followed by identification of the top 2000 most variable genes for each dataset, which were then cross-referenced across all samples to find common variable features across datasets. This information was used to perform Seurat’s FindIntegrationAnchors and IntegrateData to create a batch-correct dataset that then underwent standard dimensionality reduction and clustering as described above. Annotation of any remaining non-V1 nuclei was performed using the same marker genes outlined above. These nuclei were removed from the dataset, and this process was repeated until no further contamination was identified.

*Positive selection:* The remaining nuclei that passed QC and multiple rounds of negative selection then underwent positive selection using Delile et al.^29^ as a reference. Starting with fastq files, this reference dataset was re-analyzed using the above workflow, with cell types manually identified using the marker genes described in the original publication. A single embryonic snRNA-seq experiment was performed to obtain an age-matched E13.5 dataset enriched for V1 nuclei. After QC of this dataset, the Seurat workflow was used to annotate the query dataset with labels from the reference dataset. The E13.5 snRNA-seq dataset was then used for positive selection of the postnatal V1 interneuron nuclei already filtered by QC metrics and negative selection markers using the FindTransferAnchors method in Seurat. Nearly all nuclei (89,494 of 89,545) that passed negative selection were called as V1 neurons by this label-transfer method.

Barcodes of the identified V1 nuclei were subset from the original count matrix and re-analyzed via SCT normalization and integrated batch correction. These barcodes can be found in Supplementary Data 3. Briefly, the raw counts were log normalized and scaled per usual. SCTransform was performed for normalization and variance stabilization on each dataset individually by using the “glmGamPoi” method. The SCT-normalized object was then integrated using the standard Seurat workflow, using the P0 dataset as a reference to reduce computation time. The integrated object then underwent standard dimensionality reduction and clustering, as described above, using one of two resolutions: 0.003 to produce two clusters (Fig. 2B) or 0.24 to produce 14 clusters (Figs. 2–5).

For P0 whole spinal cord samples collected from *En1*^*Het*^ and *En1*^*KO*^ animals that formed the basis of Fig. 6, two independent biological replicates were independently processed and analyzed in Python using Scanpy (v1.9.8). Briefly, after filtering low-quality nuclei, doublets, and rarely detected genes using the QC cutoffs described above, the data was log-normalized and scaled with batch differences regressed out. No further integration was needed. Non-V1 clusters of nuclei were identified and removed using the marker genes listed in Supplementary Data 2. This dataset did not undergo positive selection.

#### Assignment of clade identity

To assign a particular cluster to a V1 clade, the expression of four clade markers (*Foxp2, Sp8, Pou6f2*, or *Chrna2* as a proxy for *MafA*) was assessed on a per-cluster basis. The average gene expression was then calculated across all nuclei within each cluster, the average gene expression was scaled, and z-scores were used to assign cluster identity. Positive z-scores were typically sufficient to assign a cluster to a single clade. Only cluster #5 had two positive z-scores, for which the higher z-score (corresponding to Sp8) was used to determine clade identity.

#### Differential gene expression to find cluster-specific genes

Differential expression was performed in Seurat with either ‘FindAllMarkers’ or ‘FindMarkers’ using the Wilcoxon rank-sum test and default parameters. *P* values were adjusted for multiple comparisons using the Bonferroni correction based on the number of genes in the dataset. Top markers were ranked by their log fold change (FC), with log_2_FCs > ±0.25 considered significant.

#### Analysis of cluster relationships

The relationships between V1 nuclei in different clusters were assessed using several methods. To generate dendrograms, an agglomerative approach was used to examine hierarchical relationships among clusters via the BuildClusterTree algorithm in Seurat on the integrated, scaled data. To evaluate the relationships among clusters based on overall gene expression, Pearson correlation coefficients were calculated on the average expression of all log-normalized genes within each cluster using the cor function in R. Relationships among clusters in PC space were assessed by aggregating counts for all clusters and running them through the DEseq2 bulk RNAseq pipeline^113^ Local inverse Simpson’s Index (LISI) scores were used to assess intermixing of nuclei across developmental age within clusters, calculated with the compute_Lisi function using two-dimensional UMAP coordinates for input^71^.

#### Gene ontology analysis

Age-related changes in gene expression were identified using the default Wilcoxon Rank Sum test in Seurat for nuclei isolated from P0 animals versus all other ages, and for P28 and P56-derived nuclei versus all other time points due to their similarity in gene expression. Genes that were positively enriched with a corrected *p* value < 0.05 were imported into ShinyGO (v0.80) for identification of the most significant gene ontology (GO) terms for Biological Processes^114^.

#### Compositional analysis of nuclei in clusters

Single-cell compositional data analysis (scCODA) was used to determine significant changes in nuclei composition between *En1*^*Het*^ and *En1*^*KO*^ snRNA-seq samples^115^. scCODA uses Bayesian modeling based on a hierarchical Dirichlet-Multinomial distribution to accommodate the limitations of snRNA-seq experiments on compositional analysis, including limited sample size, biases in cell-type correlation estimates, and the sparsity of cell-type specific manipulations. Cluster #2 was selected as a reference because it was relatively abundant in the dataset, it exhibited the smallest absolute change in composition across all four samples, and it had the least dispersion of relative abundance over all samples. Using a FDR of 0.05, only cluster #10 had a credible effect on cell type composition with a log_2_FC of -1.61 and an inclusion probability parameter of 0.97.

#### Pseudo-bulk analysis

Pseudobulk analysis was performed via summation of raw counts from all nuclei within a sample of interest. Aggregated counts were then analyzed similar to bulk RNAseq data using the DEseq2 pipeline. Briefly, a DESeq2 object was created, QC was assessed by PCA and hierarchical clustering, and then count normalization was performed and differential gene expression was analyzed using the Wald test.

### Immunohistochemistry, RNAscope in situ hybridization, and confocal imaging

Mice were deeply anesthetized via intraperitoneal injection of Avertin (Tribromoethanol, 240 mg/kg body weight) or on ice (for early postnatal animals) and transcardially perfused with PBS followed by ice-cold 4% paraformaldehyde (EMS #15714, diluted in PBS). Spinal cords were removed and post-fixed for 2 h (neonatal spinal cords) or overnight (P14, P28, and P56 spinal cords) at 4 °C, followed by cryoprotection in 30% sucrose (w/v) in 0.1 M PB for at least 24 hours at 4 °C. Tissue was embedded in OCT (Fisher #23730571), frozen, cryosectioned in the transverse plane at 20 μm, and mounted on Superfrost Plus slides (Fisher #1367811E). IHC was performed through sequential exposure to primary antibodies overnight at 4 °C, and fluorophore-conjugated secondary antibodies (Alexa Fluor Plus 488 or DyLight 488, Alexa Fluor Plus 555 or Cy3, and Alexa Fluor Plus 647 or Cy5, 1:500) for 1 h at room temperature. Sections were mounted using Fluormount-G (SouthernBiotech) or ProLong Diamond (Invitrogen P36970 without DAPI or P36971 with DAPI) and coverslipped for imaging. Confocal images were acquired on a Lecia SP8 AOBS (Leica Microsystems) confocal microscope using a 20×/0.8 NA objective, or a LSM 710 Meta Confocal microscope (Carl Zeiss) using a Plan-Apochromat 20×/0.8 M27 objective.

The following primary antibodies were used: chicken anti-β-Galactosidase (1:5000, Abcam #ab9361); rabbit anti-Calbindin D28K (1:2000, Swant #CB38); guinea pig anti-dsRed (1:16,000, Jessell Lab, RRID# AB_2923156); guinea pig anti-En1 (1:16,000, Jessell Lab, RRID# AB_3313271); guinea pig anti-FoxP1 (1:20,000, Jessell Lab, RRID# AB_2631296); goat anti-Foxp2 (1:30,000, Abcam #ab1307); goat anti-FoxP2 (1:500, Santa Cruz #sc-21069); rabbit anti-Foxp4 (1:64,000, Jessell Lab, RRID# AB_2665415); chicken anti-GFP (1:30,000, Aves #GFP-1020); rabbit anti-GFP (1:1000, Invitrogen #A-11122); guinea pig anti-Lmo3 (1:8000, Jessell Lab, RRID# AB_2665416); rabbit anti-MafA (1:2000, Novus #NB400-137); rabbit anti-MafB (1:2000, Sigma #HPA005653); mouse anti-Nr3b3 (1:2000, R&D Systems #PP-H6812-00); rabbit anti-Nr4a2 (1:500, Santa Cruz #sc-5568); goat anti-Nr5a2 (1:100, Santa Cruz #sc-21132); rabbit anti-Oc1 (1:200, Sigma #HPA003457); sheep anti-Oc2 (1:2000, R&D Systems #AF6294); guinea pig anti-Otp (1:16,000, Jessell Lab, RRID# AB_2665423); rat anti-Pou6f2 (1:16,000, Jessell Lab, RRID# AB_2665427); guinea pig anti-Prdm8 (1:10,000, Jessell Lab, RRID# AB_2665441); rabbit anti-Prox1 (1:2000, Millipore #AB5475); rabbit anti-Rnf220 (1:5000, Novus #NBP1-88487); goat anti-Sp8 (1:2000, Santa Cruz #sc-104661); goat anti-tdTomato (1:250, Sicgen #AB8181), and rabbit anti-Zfhx4 (1:1000, Sigma #HPA023837). Antibodies generously provided as gifts include rat anti-Bhlhb5 (1:5000, from Sarah Ross, RRID# AB_2665452); rabbit anti-Nr3b2 (1:2000, from Jeremy Nathans) and rat anti-St18^41^ (1:8000, from Guoqiang Gu).

Standard RNAscope ISH was performed according to the manufacturer’s instructions (Advanced Cell Diagnostics, CA) with the following modifications: drying time, 1 h; antigen retrieval, 5 min; protease treatment, 20 min. The following probes were used: Bcl11a (#563701), Dach1 (#412071), Kcnq5 (#511131), Kcnh7 (#1007281), Pax8 (#574431), St18 (#443271), and tdTomato (#317041). Signal was developed using Opal fluorophores (Akoya Biosciences) at a 1:1000 dilution, and slides were mounted in ProLong Gold (Invitrogen #P36930). For multiplexed RNAScope ISH experiments combined with immunohistochemistry, slides were stained according to the manufacturer’s instructions (Reagent Kit v2 document 323100 RevC) with a 10 min antigen retrieval and the protease-free protocol variant. The following probes were used: Calb1 (#428431), Foxp2 (#428791), Pou6f2 (#839401), Rnf220 (#839411), and Sp8 (#547521). RNAScope signal was developed with Opal 520, Opal 570, Opal 620, Opal 650, or Opal 690 (Akoya Biosciences), followed by IHC for tdT (Alexa Fluor 750). Fluorescent images were acquired on a Leica Stellaris or Leica TCS SP8 STED confocal microscope in resonant mode using a 63 × 1.4 oil objective with a 2× zoom. For quantification of multiplexed RNAScope images, a maximum intensity projection of the tdT signal was used to obtain a mask for the nuclei of V1 interneurons (ROIs) with Cellpose2. ROIs were transferred to Fiji and filtered with area and intensity thresholds. Doublets were manually removed. Intensity values were measured for each ROI for all the channels using the “Multi Measure” option. Thresholds to distinguish positive vs. negative cells for a marker were established manually. False positives were removed manually.

### Analysis of interneuron spatial distributions

The position of V1 interneurons in P0 lumbar spinal segments of *En1*^*Cre*^ heterozygous; *Tau.lsl.nLacZ* (*En1*^*Het*^) or *En1*^*Cre*^ homozygous; *Tau.lsl.nLacZ (En1*^*KO*^) mice was analyzed as previously described, using the “Spots” function in Imaris (Bitplane, Oxford Instruments Andor)^18^. Briefly, Cartesian coordinates for each interneuron were determined in the transverse spinal cord plane, with respect to the midpoint of the central canal, defined as position (0,0). All sections were normalized to a standardized spinal cord hemisection to account for variations in spinal cord dimensions along the rostrocaudal axis (distance from the central canal to the lateral boundary: 650 μm; distance from central canal to bottom-most boundary: 400 μm). Coordinates were plotted using custom Python scripts, with contours constructed using the kde2d function. Differences in spatial distributions were assessed using the two-dimensional Kolmogorov-Smirnov test implemented with the Python function ndtest (https://github.com/syrte/ndtest).

### Neuronal ablation and fictive locomotion

To ablate V1 interneurons, mice double heterozygous for Cre and Flp drivers were crossed to double homozygous *Ai65D; Tau.ds.DTR* mice. Specifically, *En1*^*Flpo*^*; Hoxb8*^*Cre*^ mice were used to target all spinal V1 interneurons below cervical level C4, and *En1*^*Cre*^*; Foxp2*^*Flpo*^ mice were used to target V1^Foxp2^ spinal interneurons. On the day of birth (P0), Diphtheria toxin (DT, List Labs, catalog # 150) or PBS (as a control) was delivered to pups via intradermal injection in the nape of the neck, at a dose of 100 ng DT/gram body weight. Animals lacking either one or both recombinases were used as controls, as these mice do not express DTR. Fictive locomotion was performed at 2–4 days post-DT or PBS administration (V1 ablation experiments) as indicated in the figure. Animals of both sexes were used. For fictive locomotion, following decapitation mice were eviscerated and then transferred to a dissecting chamber containing cold aCSF consisting of 4 mM KCl, 128 mM NaCl, 21 mM NaHCO_3_, 0.5 mM NaH_2_PO_4_ ● H_2_O, 30 mM glucose, 1 mM CaCl_2_, and 1 mM MgSO_4_ and oxygenated with 95% O_2_/5% CO_2_. After a dorsal laminectomy, the spinal cord and attached roots were continuously perfused with room temperature oxygenated aCSF containing 4 mM KCl, 128 mM NaCl, 21 mM NaHCO_3_, 0.5 mM NaH_2_PO_4_ ● H_2_O, 30 mM glucose, 2 mM CaCl_2_, 1 mM MgSO_4_, 10 µM 5-HT (Sigma #H7752), and 5 µM NMDA (Sigma #M3262). The spinal cords were allowed to recover for 30 min at room temperature before recording. Extracellular recordings from ventral roots were obtained using tight-fitting glass suction electrodes (for smaller roots Drummond #3-000-203-G/X, for larger roots A-M Systems #592800) from either the flexor-dominated left or right L2 root and/or the extensor-dominated left or right L5 root. Signals were amplified 10,000 times, bandpass filtered between 300 Hz and 500 Hz using an A-M Systems Differential AC Amplifier Model 1700, digitized at 10 kHz (Digidata 1550B; Molecular Devices), and collected using pClamp software. To calculate fictive locomotor frequency, traces were low-pass filtered using a moving mean of 10,000 points (equal to 1 s of data) and the power spectral density was calculated using the periodogram function in Scipy. The frequency corresponding to the maximum power was used to determine locomotor speed.

### Behavioral experiments (tail suspension and gait analysis)

For tail suspension experiments, P7 mice were hung by their tails at an ambient temperature of 30 °C and recorded facing their ventral side on a Basler acA720 camera at a rate of 200 frames/second for 6 min. For kinematic gait analysis, 8–10 week old mice were habituated to the testing room for at least 30 min before walking across a platform (60 cm long, 5 cm wide) toward their home cage. Three to five trials were performed for each individual mouse with at least 1 min rest between trials. Videos of tail-suspended markerless pups or locomoting adult mice were tracked using DeepLabCut (DLC)^25^. A DLC network was trained to track nine landmarks on pups (forepaw, wrist, elbow, armpit, hindpaw, ankle, knee, groin, and anus) or thirteen landmarks in adults (nose, ear, forepaw, wrist, elbow, shoulder, scapula, iliac crest, hip, knee, ankle, hind paw, and tail base). For pups, videos were then manually annotated to select frames in which all the landmarks were visible and animals were not executing turning or flipping body movements. All frames meeting these conditions (minimum of 2000 frames per animal per video) were included in our analysis. For adult animals, step cycles were manually annotated to indicate the beginning of the swing, end of swing/beginning of stance, and the end of the stance. The DLC-tracked coordinates in these frames were then analyzed using AutoGaitA^24^ to assess the following joint angles across experimental groups in pups: wrist angle (forepaw and elbow coordinates), elbow angle (wrist and armpit coordinates), ankle angle (hindpaw and knee coordinates), and knee angle (ankle and groin coordinates). In adults, AutoGaitA was used to assess joint height, ankle angle, and knee angle across experimental conditions. All statistical analyses were performed using GraphPad Prism.

### Statistics and reproducibility

No statistical methods were used to predetermine sample size. Two-tailed *t*-tests (unpaired) were used to assess significance between means, with a Holm-Sidak correction for multiple comparisons where appropriate, as indicated in the figure legends. Two-way ANOVA followed by a Holm-Sidak test for multiple comparisons was used to assess significance between mean frequency of rhythmic activity and joint angle measurements upon V1 ablation, grouped by genotype and treatment condition. Differences among groups were considered significant if *p* < 0.05. *p* values are denoted by asterisks: *p < 0.05; **p < 0.01; ***p < 0.001; ****p < 0.0001; ns – not significant. Statistical analyses were performed using GraphPad Prism software and R, and all data are represented as mean ± SEM unless otherwise indicated.

### Reporting summary

Further information on research design is available in the Nature Portfolio Reporting Summary linked to this article.

## Supplementary information

Supplementary Information

Description of Additional Supplementary Files

Supplementary Video 1

Supplementary Video 2

Supplementary Data 1

Supplementary Data 2

Supplementary Data 3

Supplementary Data 4

Supplementary Data 5

Supplementary Data 6

Reporting Summary

Transparent Peer Review file

## Source data

Source Data

## Data Availability

Raw sequencing data, counts tables, and associated metadata generated in this study have been deposited in the Gene Expression Omnibus (GEO) under accession number GEO: GSE275595. In addition, an interactive, searchable web resource for exploration and visualization of these data is available at https://v1interneurons.stjude.org/shinyApp/. Source data are provided with this paper.

## Code availability

The code used for analysis of sequencing data is available at Github (https://github.com/Bikoff-Lab/V1_interneuron_snRNAseq_Age_Atlas and https://github.com/Bikoff-Lab/V1_KO_analysis).

## References

[1] Goulding, M. Circuits controlling vertebrate locomotion: moving in a new direction. *Nat. Rev. Neurosci.* **10**, 507–518 (2009). 19543221 10.1038/nrn2608 PMC2847453

[2] Kiehn, O. Decoding the organization of spinal circuits that control locomotion. *Nat. Rev. Neurosci.* **17**, 224–238 (2016). 26935168 10.1038/nrn.2016.9 PMC4844028

[3] Eccles, J. C., Fatt, P. & Landgren, S. Central pathway for direct inhibitory action of impulses in largest afferent nerve fibres to muscle. *J. Neurophysiol.* **19**, 75–98 (1956). 13286723 10.1152/jn.1956.19.1.75

[4] Eccles, J. C., Fatt, P. & Koketsu, K. Cholinergic and inhibitory synapses in a pathway from motor-axon collaterals to motoneurones. *J. Physiol.* **126**, 524–562 (1954). 13222354 10.1113/jphysiol.1954.sp005226 PMC1365877

[5] Jankowska, E. Interneuronal relay in spinal pathways from proprioceptors. *Prog. Neurobiol.* **38**, 335–378 (1992). 1315446 10.1016/0301-0082(92)90024-9

[6] Rudomin, P. In search of lost presynaptic inhibition. *Exp. Brain Res* **196**, 139–151 (2009). 19322562 10.1007/s00221-009-1758-9

[7] Cote, M. P., Murray, L. M. & Knikou, M. Spinal Control of Locomotion: Individual Neurons, Their Circuits and Functions. *Front Physiol.* **9**, 784 (2018). 29988534 10.3389/fphys.2018.00784 PMC6026662

[8] Jessell, T. M. Neuronal specification in the spinal cord: inductive signals and transcriptional codes. *Nat. Rev. Genet* **1**, 20–29 (2000). 11262869 10.1038/35049541

[9] Sengupta, M. & Bagnall, M. W. Spinal Interneurons: Diversity and Connectivity in Motor Control. *Annu Rev. Neurosci.* **46**, 79–99 (2023). 36854318 10.1146/annurev-neuro-083122-025325 PMC11580138

[10] Crone, S. A., Zhong, G., Harris-Warrick, R. & Sharma, K. In mice lacking V2a interneurons, gait depends on speed of locomotion. *J. Neurosci.* **29**, 7098–7109 (2009). 19474336 10.1523/JNEUROSCI.1206-09.2009 PMC2731420

[11] Gosgnach, S. et al. V1 spinal neurons regulate the speed of vertebrate locomotor outputs. *Nature* **440**, 215–219 (2006). 16525473 10.1038/nature04545

[12] Lanuza, G. M., Gosgnach, S., Pierani, A., Jessell, T. M. & Goulding, M. Genetic identification of spinal interneurons that coordinate left-right locomotor activity necessary for walking movements. *Neuron* **42**, 375–386 (2004). 15134635 10.1016/s0896-6273(04)00249-1

[13] Talpalar, A. E. et al. Dual-mode operation of neuronal networks involved in left-right alternation. *Nature* **500**, 85–88 (2013). 23812590 10.1038/nature12286

[14] Zhang, Y. et al. V3 spinal neurons establish a robust and balanced locomotor rhythm during walking. *Neuron* **60**, 84–96 (2008). 18940590 10.1016/j.neuron.2008.09.027 PMC2753604

[15] Zhang, J. et al. V1 and v2b interneurons secure the alternating flexor-extensor motor activity mice require for limbed locomotion. *Neuron* **82**, 138–150 (2014). 24698273 10.1016/j.neuron.2014.02.013 PMC4096991

[16] Lu, D. C., Niu, T. & Alaynick, W. A. Molecular and cellular development of spinal cord locomotor circuitry. *Front Mol. Neurosci.* **8**, 25 (2015). 26136656 10.3389/fnmol.2015.00025 PMC4468382

[17] Osseward, P. J. 2nd & Pfaff, S. L. Cell type and circuit modules in the spinal cord. *Curr. Opin. Neurobiol.* **56**, 175–184 (2019). 30954861 10.1016/j.conb.2019.03.003 PMC8559966

[18] Bikoff, J. B. et al. Spinal Inhibitory Interneuron Diversity Delineates Variant Motor Microcircuits. *Cell* **165**, 207–219 (2016). 26949184 10.1016/j.cell.2016.01.027 PMC4808435

[19] Gabitto, M. I. et al. Bayesian Sparse Regression Analysis Documents the Diversity of Spinal Inhibitory Interneurons. *Cell* **165**, 220–233 (2016). 26949187 10.1016/j.cell.2016.01.026 PMC4831714

[20] Britz, O. et al. A genetically defined asymmetry underlies the inhibitory control of flexor-extensor locomotor movements. *Elife* **4**, e04718 (2015). 10.7554/eLife.04718 PMC4604447 26465208

[21] Allodi, I., Montanana-Rosell, R., Selvan, R., Low, P. & Kiehn, O. Locomotor deficits in a mouse model of ALS are paralleled by loss of V1-interneuron connections onto fast motor neurons. *Nat. Commun.* **12**, 3251 (2021). 34059686 10.1038/s41467-021-23224-7 PMC8166981

[22] Ericson, J. et al. Pax6 controls progenitor cell identity and neuronal fate in response to graded Shh signaling. *Cell* **90**, 169–180 (1997). 9230312 10.1016/s0092-8674(00)80323-2

[23] Vijatovic, D. et al. Multifold increase in spinal inhibitory cell types with emergence of limb movement. *Cell Rep.* **45**, 117227 (2026). 41964955 10.1016/j.celrep.2026.117227

[24] Hosseini, M. et al. AutoGaitA – Automated Gait Analysis in Python. *bioRxiv*, 2024.2004.2014.589409 (2024).

[25] Mathis, A. et al. DeepLabCut: markerless pose estimation of user-defined body parts with deep learning. *Nat. Neurosci.* **21**, 1281–1289 (2018). 30127430 10.1038/s41593-018-0209-y

[26] Witschi, R. et al. Hoxb8-Cre mice: A tool for brain-sparing conditional gene deletion. *Genesis* **48**, 596–602 (2010). 20658520 10.1002/dvg.20656 PMC3566526

[27] Kimmel, R. A. et al. Two lineage boundaries coordinate vertebrate apical ectodermal ridge formation. *Genes Dev.* **14**, 1377–1389 (2000). 10837030 PMC316660

[28] Worthy, A. E. et al. Spinal V1 inhibitory interneuron clades differ in birthdate, projections to motoneurons, and heterogeneity. *Elife* **13**, RP95172 (2024). 10.7554/eLife.95172 PMC11604222 39607843

[29] Delile, J. et al. Single cell transcriptomics reveals spatial and temporal dynamics of gene expression in the developing mouse spinal cord. *Development* **146** (2019). 10.1242/dev.173807 PMC6602353 30846445

[30] Haring, M. et al. Neuronal atlas of the dorsal horn defines its architecture and links sensory input to transcriptional cell types. *Nat. Neurosci.* **21**, 869–880 (2018). 29686262 10.1038/s41593-018-0141-1

[31] Rosenberg, A. B. et al. Single-cell profiling of the developing mouse brain and spinal cord with split-pool barcoding. *Science* **360**, 176–182 (2018). 29545511 10.1126/science.aam8999 PMC7643870

[32] Russ, D. E. et al. A harmonized atlas of mouse spinal cord cell types and their spatial organization. *Nat. Commun.* **12**, 5722 (2021). 34588430 10.1038/s41467-021-25125-1 PMC8481483

[33] Sathyamurthy, A. et al. Massively Parallel Single Nucleus Transcriptional Profiling Defines Spinal Cord Neurons and Their Activity during Behavior. *Cell Rep.* **22**, 2216–2225 (2018). 29466745 10.1016/j.celrep.2018.02.003 PMC5849084

[34] Zeisel, A. et al. Molecular Architecture of the Mouse Nervous System. *Cell* **174**, 999–1014.e1022 (2018). 30096314 10.1016/j.cell.2018.06.021 PMC6086934

[35] Saunders, A. et al. Molecular Diversity and Specializations among the Cells of the Adult Mouse Brain. *Cell* **174**, 1015–1030.e1016 (2018). 30096299 10.1016/j.cell.2018.07.028 PMC6447408

[36] Wu, Y. E., Pan, L., Zuo, Y., Li, X. & Hong, W. Detecting Activated Cell Populations Using Single-Cell RNA-Seq. *Neuron* **96**, 313–329.e316 (2017). 29024657 10.1016/j.neuron.2017.09.026

[37] Bakken, T. E. et al. Single-nucleus and single-cell transcriptomes compared in matched cortical cell types. *PLoS One* **13**, e0209648 (2018). 30586455 10.1371/journal.pone.0209648 PMC6306246

[38] Mo, A. et al. Epigenomic Signatures of Neuronal Diversity in the Mammalian Brain. *Neuron* **86**, 1369–1384 (2015). 26087164 10.1016/j.neuron.2015.05.018 PMC4499463

[39] Stuart, T. et al. Comprehensive Integration of Single-Cell Data. *Cell* **177**, 1888–1902.e1821 (2019). 31178118 10.1016/j.cell.2019.05.031 PMC6687398

[40] Osseward, P. J. et al. Conserved genetic signatures parcellate cardinal spinal neuron classes into local and projection subsets. *Science* **372**, 385–393 (2021). 33888637 10.1126/science.abe0690 PMC8612134

[41] Nunnelly, L. F. et al. St18 specifies globus pallidus projection neuron identity in MGE lineage. *Nat. Commun.* **13**, 7735 (2022). 36517477 10.1038/s41467-022-35518-5 PMC9751150

[42] Hayashi, M. et al. Graded Arrays of Spinal and Supraspinal V2a Interneuron Subtypes Underlie Forelimb and Hindlimb Motor Control. *Neuron* **97**, 869–884.e865 (2018). 29398364 10.1016/j.neuron.2018.01.023 PMC8601153

[43] Sweeney, L. B. et al. Origin and Segmental Diversity of Spinal Inhibitory Interneurons. *Neuron* **97**, 341–355.e343 (2018). 29307712 10.1016/j.neuron.2017.12.029 PMC5880537

[44] Kim, J. et al. Rnf220 cooperates with Zc4h2 to specify spinal progenitor domains. *Development* **145**, dev165340 (2018). 10.1242/dev.165340 30177510

[45] Ma, P. et al. RNF220 is an E3 ubiquitin ligase for AMPA receptors to regulate synaptic transmission. *Sci. Adv.* **8**, eabq4736 (2022). 36179027 10.1126/sciadv.abq4736 PMC9524831

[46] Stam, F. J. et al. Renshaw cell interneuron specialization is controlled by a temporally restricted transcription factor program. *Development* **139**, 179–190 (2012). 22115757 10.1242/dev.071134 PMC3231776

[47] Bayer, H. et al. ALS-causing mutations differentially affect PGC-1alpha expression and function in the brain vs. peripheral tissues. *Neurobiol. Dis.* **97**, 36–45 (2017). 27818323 10.1016/j.nbd.2016.11.001

[48] Galazo, M. J., Sweetser, D. A. & Macklis, J. D. Tle4 controls both developmental acquisition and early post-natal maturation of corticothalamic projection neuron identity. *Cell Rep.* **42**, 112957 (2023). 37561632 10.1016/j.celrep.2023.112957 PMC10542749

[49] Rataj-Baniowska, M. et al. Retinoic Acid Receptor beta Controls Development of Striatonigral Projection Neurons through FGF-Dependent and Meis1-Dependent Mechanisms. *J. Neurosci.* **35**, 14467–14475 (2015). 26511239 10.1523/JNEUROSCI.1278-15.2015 PMC6605468

[50] Gauberg, J. et al. Spinal motor neuron development and metabolism are transcriptionally regulated by Nuclear Factor IA. *Sci. Adv.* **11**, eadu3346 (2025). 10.1126/sciadv.adu3346 PMC12315974 40749054

[51] Deneen, B. et al. The transcription factor NFIA controls the onset of gliogenesis in the developing spinal cord. *Neuron* **52**, 953–968 (2006). 17178400 10.1016/j.neuron.2006.11.019

[52] Fraser, J. et al. Common Regulatory Targets of NFIA, NFIX and NFIB during Postnatal Cerebellar Development. *Cerebellum* **19**, 89–101 (2020). 31838646 10.1007/s12311-019-01089-3 PMC7815246

[53] Garcia-Dominguez, M., Poquet, C., Garel, S. & Charnay, P. Ebf gene function is required for coupling neuronal differentiation and cell cycle exit. *Development* **130**, 6013–6025 (2003). 14573522 10.1242/dev.00840

[54] Caubit, X. et al. TSHZ3 deletion causes an autism syndrome and defects in cortical projection neurons. *Nat. Genet* **48**, 1359–1369 (2016). 27668656 10.1038/ng.3681 PMC5083212

[55] Hanley, O. et al. Parallel Pbx-Dependent Pathways Govern the Coalescence and Fate of Motor Columns. *Neuron* **91**, 1005–1020 (2016). 27568519 10.1016/j.neuron.2016.07.043 PMC5017921

[56] Wang, B. et al. Zfp462 deficiency causes anxiety-like behaviors with excessive self-grooming in mice. *Genes Brain Behav.* **16**, 296–307 (2017). 27621227 10.1111/gbb.12339

[57] Yelagandula, R. et al. ZFP462 safeguards neural lineage specification by targeting G9A/GLP-mediated heterochromatin to silence enhancers. *Nat. Cell Biol.* **25**, 42–55 (2023). 36604593 10.1038/s41556-022-01051-2 PMC10038669

[58] Harris, A. et al. Onecut Factors and Pou2f2 Regulate the Distribution of V2 Interneurons in the Mouse Developing Spinal Cord. *Front Cell Neurosci.* **13**, 184 (2019). 31231191 10.3389/fncel.2019.00184 PMC6561314

[59] Oyama, T. et al. Mastermind-like 1 (MamL1) and mastermind-like 3 (MamL3) are essential for Notch signaling in vivo. *Development* **138**, 5235–5246 (2011). 22069191 10.1242/dev.062802

[60] Burrill, J. D., Moran, L., Goulding, M. D. & Saueressig, H. PAX2 is expressed in multiple spinal cord interneurons, including a population of EN1+ interneurons that require PAX6 for their development. *Development* **124**, 4493–4503 (1997). 9409667 10.1242/dev.124.22.4493

[61] Pillai, A., Mansouri, A., Behringer, R., Westphal, H. & Goulding, M. Lhx1 and Lhx5 maintain the inhibitory-neurotransmitter status of interneurons in the dorsal spinal cord. *Development* **134**, 357–366 (2007). 17166926 10.1242/dev.02717

[62] Huang, H. & Trussell, L. O. KCNQ5 channels control resting properties and release probability of a synapse. *Nat. Neurosci.* **14**, 840–847 (2011). 21666672 10.1038/nn.2830 PMC3133966

[63] Xiao, K. et al. ERG3 potassium channel-mediated suppression of neuronal intrinsic excitability and prevention of seizure generation in mice. *J. Physiol.* **596**, 4729–4752 (2018). 30016551 10.1113/JP275970 PMC6166062

[64] Ranade, S. S. et al. Piezo2 is the major transducer of mechanical forces for touch sensation in mice. *Nature* **516**, 121–125 (2014). 25471886 10.1038/nature13980 PMC4380172

[65] Woo, S. H. et al. Piezo2 is the principal mechanotransduction channel for proprioception. *Nat. Neurosci.* **18**, 1756–1762 (2015). 26551544 10.1038/nn.4162 PMC4661126

[66] Patel, T. et al. Transcriptional dynamics of murine motor neuron maturation in vivo and in vitro. *Nat. Commun.* **13**, 5427 (2022). 36109497 10.1038/s41467-022-33022-4 PMC9477853

[67] Benito-Gonzalez, A. & Alvarez, F. J. Renshaw cells and Ia inhibitory interneurons are generated at different times from p1 progenitors and differentiate shortly after exiting the cell cycle. *J. Neurosci.* **32**, 1156–1170 (2012). 22279202 10.1523/JNEUROSCI.3630-12.2012 PMC3276112

[68] Li, H. et al. Classifying Drosophila Olfactory Projection Neuron Subtypes by Single-Cell RNA Sequencing. *Cell* **171**, 1206–1220.e1222 (2017). 29149607 10.1016/j.cell.2017.10.019 PMC6095479

[69] Roome, R. B. & Levine, A. J. The organization of spinal neurons: Insights from single cell sequencing. *Curr. Opin. Neurobiol.* **82**, 102762 (2023). 37657185 10.1016/j.conb.2023.102762 PMC10727478

[70] Tasic, B. et al. Shared and distinct transcriptomic cell types across neocortical areas. *Nature* **563**, 72–78 (2018). 30382198 10.1038/s41586-018-0654-5 PMC6456269

[71] Korsunsky, I. et al. Fast, sensitive and accurate integration of single-cell data with Harmony. *Nat. Methods* **16**, 1289–1296 (2019). 31740819 10.1038/s41592-019-0619-0 PMC6884693

[72] Okaty, B. W., Miller, M. N., Sugino, K., Hempel, C. M. & Nelson, S. B. Transcriptional and electrophysiological maturation of neocortical fast-spiking GABAergic interneurons. *J. Neurosci.* **29**, 7040–7052 (2009). 19474331 10.1523/JNEUROSCI.0105-09.2009 PMC2749660

[73] Prieur, D. S., Francius, C., Gaspar, P., Mason, C. A. & Rebsam, A. Semaphorin-6D and Plexin-A1 Act in a Non-Cell-Autonomous Manner to Position and Target Retinal Ganglion Cell Axons. *J. Neurosci.* **43**, 5769–5778 (2023). 37344233 10.1523/JNEUROSCI.0072-22.2023 PMC10423046

[74] Washbourne, P. et al. Genetic ablation of the t-SNARE SNAP-25 distinguishes mechanisms of neuroexocytosis. *Nat. Neurosci.* **5**, 19–26 (2002). 11753414 10.1038/nn783

[75] Matise, M. P. & Joyner, A. L. Expression patterns of developmental control genes in normal and Engrailed-1 mutant mouse spinal cord reveal early diversity in developing interneurons. *J. Neurosci.* **17**, 7805–7816 (1997). 9315901 10.1523/JNEUROSCI.17-20-07805.1997 PMC6793918

[76] Saueressig, H., Burrill, J. & Goulding, M. Engrailed-1 and netrin-1 regulate axon pathfinding by association interneurons that project to motor neurons. *Development* **126**, 4201–4212 (1999). 10477289 10.1242/dev.126.19.4201

[77] Bellardita, C. & Kiehn, O. Phenotypic characterization of speed-associated gait changes in mice reveals modular organization of locomotor networks. *Curr. Biol.* **25**, 1426–1436 (2015). 25959968 10.1016/j.cub.2015.04.005 PMC4469368

[78] Caggiano, V. et al. Midbrain circuits that set locomotor speed and gait selection. *Nature* **553**, 455–460 (2018). 29342142 10.1038/nature25448 PMC5937258

[79] Capelli, P., Pivetta, C., Soledad Esposito, M. & Arber, S. Locomotor speed control circuits in the caudal brainstem. *Nature* **551**, 373–377 (2017). 29059682 10.1038/nature24064

[80] Crone, S. A. et al. Genetic ablation of V2a ipsilateral interneurons disrupts left-right locomotor coordination in mammalian spinal cord. *Neuron* **60**, 70–83 (2008). 18940589 10.1016/j.neuron.2008.08.009

[81] Haque, F. et al. WT1-Expressing Interneurons Regulate Left-Right Alternation during Mammalian Locomotor Activity. *J. Neurosci.* **38**, 5666–5676 (2018). 29789381 10.1523/JNEUROSCI.0328-18.2018 PMC6595980

[82] Callahan, R. A. et al. Spinal V2b neurons reveal a role for ipsilateral inhibition in speed control. *Elife* **8**, e47837 (2019). 10.7554/eLife.47837 PMC6701946 31355747

[83] Ampatzis, K., Song, J., Ausborn, J. & El Manira, A. Separate microcircuit modules of distinct v2a interneurons and motoneurons control the speed of locomotion. *Neuron* **83**, 934–943 (2014). 25123308 10.1016/j.neuron.2014.07.018

[84] Bizzi, E., Cheung, V. C., d’Avella, A., Saltiel, P. & Tresch, M. Combining modules for movement. *Brain Res Rev.* **57**, 125–133 (2008). 18029291 10.1016/j.brainresrev.2007.08.004 PMC4295773

[85] Pallucchi, I. et al. Molecular blueprints for spinal circuit modules controlling locomotor speed in zebrafish. *Nat. Neurosci.* **27**, 78–89 (2024). 37919423 10.1038/s41593-023-01479-1 PMC10774144

[86] Yao, Z. et al. A high-resolution transcriptomic and spatial atlas of cell types in the whole mouse brain. *Nature* **624**, 317–332 (2023). 38092916 10.1038/s41586-023-06812-z PMC10719114

[87] Baek, M., Menon, V., Jessell, T. M., Hantman, A. W. & Dasen, J. S. Molecular Logic of Spinocerebellar Tract Neuron Diversity and Connectivity. *Cell Rep.* **27**, 2620–2635.e2624 (2019). 31141687 10.1016/j.celrep.2019.04.113 PMC6555431

[88] Alkaslasi, M. R. et al. Single nucleus RNA-sequencing defines unexpected diversity of cholinergic neuron types in the adult mouse spinal cord. *Nat. Commun.* **12**, 2471 (2021). 33931636 10.1038/s41467-021-22691-2 PMC8087807

[89] Blum, J. A. et al. Single-cell transcriptomic analysis of the adult mouse spinal cord reveals molecular diversity of autonomic and skeletal motor neurons. *Nat. Neurosci.* **24**, 572–583 (2021). 33589834 10.1038/s41593-020-00795-0 PMC8016743

[90] Liau, E. S. et al. Single-cell transcriptomic analysis reveals diversity within mammalian spinal motor neurons. *Nat. Commun.* **14**, 46 (2023). 36596814 10.1038/s41467-022-35574-x PMC9810664

[91] Chapman, P. D. et al. A brain-wide map of descending inputs onto spinal V1 interneurons. *Neuron* **113**, 524–538 (2025). 10.1016/j.neuron.2024.11.019 PMC11842218 39719703

[92] Bouvier, J. et al. Descending Command Neurons in the Brainstem that Halt Locomotion. *Cell* **163**, 1191–1203 (2015). 26590422 10.1016/j.cell.2015.10.074 PMC4899047

[93] Murray, A. J., Croce, K., Belton, T., Akay, T. & Jessell, T. M. Balance Control Mediated by Vestibular Circuits Directing Limb Extension or Antagonist Muscle Co-activation. *Cell Rep.* **22**, 1325–1338 (2018). 29386118 10.1016/j.celrep.2018.01.009

[94] Cregg, J. M. et al. Brainstem neurons that command mammalian locomotor asymmetries. *Nat. Neurosci.* **23**, 730–740 (2020). 32393896 10.1038/s41593-020-0633-7 PMC7610510

[95] Usseglio, G., Gatier, E., Heuze, A., Herent, C. & Bouvier, J. Control of Orienting Movements and Locomotion by Projection-Defined Subsets of Brainstem V2a Neurons. *Curr. Biol.* **30**, 4665–4681.e4666 (2020). 33007251 10.1016/j.cub.2020.09.014

[96] Kimura, Y. & Higashijima, S. I. Regulation of locomotor speed and selection of active sets of neurons by V1 neurons. *Nat. Commun.* **10**, 2268 (2019). 31118414 10.1038/s41467-019-09871-x PMC6531463

[97] Shevtsova, N. A., Li, E. Z., Singh, S., Dougherty, K. J. & Rybak, I. A. Ipsilateral and Contralateral Interactions in Spinal Locomotor Circuits Mediated by V1 Neurons: Insights from Computational Modeling. *Int. J. Mol. Sci.* **23**, 5541 (2022). 10.3390/ijms23105541 PMC9146873 35628347

[98] Sapir, T. et al. Pax6 and engrailed 1 regulate two distinct aspects of renshaw cell development. *J. Neurosci.* **24**, 1255–1264 (2004). 14762144 10.1523/JNEUROSCI.3187-03.2004 PMC2997484

[99] Gosgnach, S. et al. Delineating the Diversity of Spinal Interneurons in Locomotor Circuits. *J. Neurosci.* **37**, 10835–10841 (2017). 29118212 10.1523/JNEUROSCI.1829-17.2017 PMC6596484

[100] Moran-Rivard, L. et al. Evx1 is a postmitotic determinant of v0 interneuron identity in the spinal cord. *Neuron* **29**, 385–399 (2001). 11239430 10.1016/s0896-6273(01)00213-6

[101] Clovis, Y. M. et al. Chx10 Consolidates V2a Interneuron Identity through Two Distinct Gene Repression Modes. *Cell Rep.* **16**, 1642–1652 (2016). 27477290 10.1016/j.celrep.2016.06.100 PMC8109239

[102] Blacklaws, J. et al. Sim1 is required for the migration and axonal projections of V3 interneurons in the developing mouse spinal cord. *Dev. Neurobiol.* **75**, 1003–1017 (2015). 25652362 10.1002/dneu.22266

[103] Simon, H. H., Saueressig, H., Wurst, W., Goulding, M. D. & O’Leary, D. D. Fate of midbrain dopaminergic neurons controlled by the engrailed genes. *J. Neurosci.* **21**, 3126–3134 (2001). 11312297 10.1523/JNEUROSCI.21-09-03126.2001 PMC6762576

[104] Sagner, A. & Briscoe, J. Establishing neuronal diversity in the spinal cord: a time and a place. *Development* **146**, dev182154 (2019). 10.1242/dev.182154 31767567

[105] Yadav, A. et al. A cellular taxonomy of the adult human spinal cord. *Neuron* **111**, 328–344.e327 (2023). 36731429 10.1016/j.neuron.2023.01.007 PMC10044516

[106] Network, B. I. C. C. A multimodal cell census and atlas of the mammalian primary motor cortex. *Nature* **598**, 86–102 (2021). 34616075 10.1038/s41586-021-03950-0 PMC8494634

[107] Zeng, H. What is a cell type and how to define it?. *Cell* **185**, 2739–2755 (2022). 35868277 10.1016/j.cell.2022.06.031 PMC9342916

[108] Clarac, F., Brocard, F. & Vinay, L. The maturation of locomotor networks. *Prog. Brain Res* **143**, 57–66 (2004). 14653151 10.1016/S0079-6123(03)43006-9

[109] Pivetta, C., Esposito, M. S., Sigrist, M. & Arber, S. Motor-circuit communication matrix from spinal cord to brainstem neurons revealed by developmental origin. *Cell* **156**, 537–548 (2014). 24485459 10.1016/j.cell.2013.12.014

[110] Lane, A. R., Cogdell, I. C., Jessell, T. M., Bikoff, J. B. & Alvarez, F. J. Genetic targeting of adult Renshaw cells using a Calbindin 1 destabilized Cre allele for intersection with Parvalbumin or Engrailed1. *Sci. Rep.* **11**, 19861 (2021). 34615947 10.1038/s41598-021-99333-6 PMC8494874

[111] Matson, K. J. E. et al. Isolation of Adult Spinal Cord Nuclei for Massively Parallel Single-nucleus RNA Sequencing. *J. Vis. Exp.* **140**, e58413 (2018). 10.3791/58413 PMC6235529 30371670

[112] Young, M. D. & Behjati, S. SoupX removes ambient RNA contamination from droplet-based single-cell RNA sequencing data. *Gigascience* **9**, giaa151 (2020). 10.1093/gigascience/giaa151 PMC7763177 33367645

[113] Love, M. I., Huber, W. & Anders, S. Moderated estimation of fold change and dispersion for RNA-seq data with DESeq2. *Genome Biol.* **15**, 550 (2014). 25516281 10.1186/s13059-014-0550-8 PMC4302049

[114] Ge, S. X., Jung, D. & Yao, R. ShinyGO: a graphical gene-set enrichment tool for animals and plants. *Bioinformatics* **36**, 2628–2629 (2020). 31882993 10.1093/bioinformatics/btz931 PMC7178415

[115] Buttner, M., Ostner, J., Muller, C. L., Theis, F. J. & Schubert, B. scCODA is a Bayesian model for compositional single-cell data analysis. *Nat. Commun.* **12**, 6876 (2021). 34824236 10.1038/s41467-021-27150-6 PMC8616929

