# Supplementary material for: Transcriptomic analysis of spinal V1 interneurons informs their multifunctional role in motor output
Source: Nat Commun. 2026 Aug 10;17:9614. doi: 10.1038/s41467-026-76522-3 (PMC13554063; doi:10.1038/s41467-026-76522-3)
Supplement: Supplementary file 1 [file 41467_2026_76522_MOESM11_ESM.pdf]

nature portfolio

Corresponding author(s): Jay B. Bikoff

Last updated by author(s): Jul 13, 2026

Reporting Summary

Nature Portfolio wishes to improve the reproducibility of the work that we publish. This form provides structure for consistency and transparency in reporting. For further information on Nature Portfolio policies, see our Editorial Policies and the Editorial Policy Checklist.

Statistics

For all statistical analyses, confirm that the following items are present in the figure legend, table legend, main text, or Methods section.

n/a	Confirmed	
☐	☒	The exact sample size (*n*) for each experimental group/condition, given as a discrete number and unit of measurement
☐	☒	A statement on whether measurements were taken from distinct samples or whether the same sample was measured repeatedly
☐	☒	The statistical test(s) used AND whether they are one- or two-sided<br>*Only common tests should be described solely by name; describe more complex techniques in the Methods section.*
☐	☒	A description of all covariates tested
☐	☒	A description of any assumptions or corrections, such as tests of normality and adjustment for multiple comparisons
☐	☒	A full description of the statistical parameters including central tendency (e.g. means) or other basic estimates (e.g. regression coefficient) AND variation (e.g. standard deviation) or associated estimates of uncertainty (e.g. confidence intervals)
☐	☒	For null hypothesis testing, the test statistic (e.g. *F*, *t*, *r*) with confidence intervals, effect sizes, degrees of freedom and *P* value noted<br>*Give P values as exact values whenever suitable.*
☒	☐	For Bayesian analysis, information on the choice of priors and Markov chain Monte Carlo settings
☒	☐	For hierarchical and complex designs, identification of the appropriate level for tests and full reporting of outcomes
☐	☒	Estimates of effect sizes (e.g. Cohen's *d*, Pearson's *r*), indicating how they were calculated

Our web collection on statistics for biologists contains articles on many of the points above.

Software and code

Policy information about availability of computer code

Data collection	The 10x Genomics Platform was used to generate single-nucleus gel beads-in-emulsions (GEMs) from mouse spinal cord tissue, using the Chromium Next GEM Single Cell 3’ GEM, Library, and Gel Bead Kit v3.1 (#1000121) and Chromium Single Cell B Chip Kit (#1000120),
Data analysis	Single nucleus RNA-seq data processing and downstream analyses of mouse V1 interneurons is available at Github (https://github.com/Bikoff-Lab/V1_interneuron_snRNAseq_Age_Atlas and https://github.com/Bikoff-Lab/V1_KO_analysis). The following software was used:<br>CellRanger (v7.0.0 or v3.0.0)<br>Seurat (v4.3.0)<br>GraphPad Prism (v9)<br>Fiji/ImageJ<br>SoupX<br>Python (v3.12.1)<br>Scanpy(v1.9.8)<br>DEseq2(v1.38.3)<br>R (v4.3.3)

For manuscripts utilizing custom algorithms or software that are central to the research but not yet described in published literature, software must be made available to editors and reviewers. We strongly encourage code deposition in a community repository (e.g. GitHub). See the Nature Portfolio guidelines for submitting code & software for further information.

Data

Policy information about availability of data

All manuscripts must include a data availability statement. This statement should provide the following information, where applicable:

- Accession codes, unique identifiers, or web links for publicly available datasets
- A description of any restrictions on data availability
- For clinical datasets or third party data, please ensure that the statement adheres to our policy

The raw sequencing data, counts tables, and associated metadata are deposited in the Gene Expression Omnibus (GEO) with accession number GEO: GSE275595. In addition, the publicly available data from Delile et al. used in this study can be found in ArrayExpress under accession number E-MTAB-7320.

An interactive, searchable web resource for exploration and visualization of these data is available at https://v1interneurons.stjude.org/shinyApp/.

Source data are provided with this paper.

Research involving human participants, their data, or biological material

Policy information about studies with human participants or human data. See also policy information about sex, gender (identity/presentation), and sexual orientation and race, ethnicity and racism.

Reporting on sex and gender	N/A
Reporting on race, ethnicity, or other socially relevant groupings	N/A
Population characteristics	N/A
Recruitment	N/A
Ethics oversight	N/A

Note that full information on the approval of the study protocol must also be provided in the manuscript.

Field-specific reporting

Please select the one below that is the best fit for your research. If you are not sure, read the appropriate sections before making your selection.

☒ Life sciences ☐ Behavioural & social sciences ☐ Ecological, evolutionary & environmental sciences

For a reference copy of the document with all sections, see nature.com/documents/nr-reporting-summary-flat.pdf

Life sciences study design

All studies must disclose on these points even when the disclosure is negative.

Sample size	For all animal studies, mice of both sexes were used. Single-nucleus transcriptomic experiments were were performed by loading 8,000 nuclei per sample, resulting in a total of 89,494 nuclei (V1 transcriptomic atlas) or 14,971 (En1 Het vs KO experiments) that passed QC metrics. No formal statistical sample size calculation was performed to determine sample size for other experiments. The biological N for these experiments is equal to or greater than commonly accepted standards in the field.
Data exclusions	Of the 24 samples used for the V1 interneuron transcriptomic atlas, one (P56LR1) was removed due to high ambient RNA identified by SoupX. Each dataset underwent individual quality control (QC) to remove low quality data and doublets. Nuclei were filtered out if they contained <1000 unique molecular identifiers (UMIs), >2.5% of transcripts derived from mitochondrial genes, or were above or below ±3 median absolute deviations (MADs) from the median number of UMIs and genes per sample. Any gene that appeared in <10 cells was removed.
Replication	All transcriptomic experiments involved two biological replicates. All other experiments, including physiology, behavioral analyses, and immunohistochemical analyses were performed on at least three independent animals, with the specific number of replicates reported in the text and/or figure legends.
Randomization	Randomization was not performed, though mice of both sexes were included in all analyses.
Blinding	The experimenter was blinded during data collection and analysis when comparing genotypes for microscopy or functional studies including behavior and electrophysiology,

Reporting for specific materials, systems and methods

We require information from authors about some types of materials, experimental systems and methods used in many studies. Here, indicate whether each material, system or method listed is relevant to your study. If you are not sure if a list item applies to your research, read the appropriate section before selecting a response.

Materials & experimental systems

n/a	Involved in the study
☐	☒ Antibodies
☒	☐ Eukaryotic cell lines
☒	☐ Palaeontology and archaeology
☐	☒ Animals and other organisms
☒	☐ Clinical data
☒	☐ Dual use research of concern
☒	☐ Plants

Methods

n/a	Involved in the study
☒	☐ ChIP-seq
☐	☒ Flow cytometry
☒	☐ MRI-based neuroimaging

Antibodies

Antibodies used	Chick anti-beta-Galactosidase (Abcam ab9361), 1:5000<br>Rat anti-Bhlhb5 (Sara Ross, RRID# AB_2665452), 1:5000<br>Rabbit anti-Calbindin1 (Swant CB38), 1:2000<br>Guinea pig anti-dsRed (Jessell Lab/HHMI CU, RRID# AB_2923156), 1:16000<br>Guinea pig anti-En1 (Jessell Lab/HHMI CU, RRID# AB_3313271), 1:16000<br>Guinea pig anti-Foxp1 (Jessell Lab/HHMI CU, RRID# AB_2631296), 1:20000<br>Goat anti-Foxp2 (Santa Cruz 21069), 1:500<br>Goat anti-FoxP2 (Abcam 1307), 1:30000<br>Rabbit anti-FoxP4 (Jessell/HHMI CU, RRID# AB_2665415), 1:64000<br>Rabbit anti-GFP (Invitrogen A11122), 1:1000<br>Chicken anti-GFP (Aves 1020), 1:30000<br>Guinea pig anti-Lmo3 (Jessell/HHMI CU, RRID# AB_2665416), 1:8000<br>Rabbit anti-MafA (Novus NB400-137), 1:2000<br>Rabbit anti-MafB (Sigma HPA005653), 1:2000<br>Rabbit anti-Nr3b2 (Jeremy Nathans), 1:2000<br>Mouse anti-Nr3b3 (R&D H6812-00), 1:2000<br>Rabbit anti-Nr4a2 (Santa Cruz 5568), 1:500<br>Goat anti-Nr5a2 (Santa Cruz 21132), 1:100<br>Rabbit anti-Oc1 (Sigma HPA003457), 1:200<br>Sheep anti-Oc2 (R&D AF6294), 1:2000<br>Guinea pig anti-Otp (Jessell/HHMI CU, RRID# AB_2665423), 1:16000<br>Rat anti-Pou6f2 (Jessell Lab /HHMI CU, RRID# AB_2665427), 1:16000<br>Guinea pig anti-Prdm8 (Jessell Lab / HHMI CU, RRID# AB_2665441), 1:10000<br>Rabbit anti-Prox1 (Millipore AB5475), 1:2000<br>Rabbit anti-Rnf220 (Novus 103137), 1:5000<br>Goat anti-Sp8 (Santa Cruz 104661), 1:2000<br>Rat anti-St18 (Guogiang Gu), 1:8000<br>Goat anti-tdTomato (Sicgen AB8181), 1:250<br>Rabbit anti-Zfhx4 (Sigma HPA023837), 1:1000
Validation	When possible, antibodies were validated for specificity against knockout tissue. References for previously characterized antibodies are provided in the text and methods sections.

Animals and other research organisms

Policy information about studies involving animals; ARRIVE guidelines recommended for reporting animal research, and Sex and Gender in Research

Laboratory animals	The following previously published mouse lines were used in this study: C57BL/6J (Jackson Laboratory #000664), RC.lsl.tdT (Ai14) (Jackson Laboratory #007914), RC.fsf.tdT (Ai65F) (Jackson Laboratory #032864), RC.dual.tdT (Ai65D) (Jackson Laboratory #021875), RC.lsl.ntdT (Ai75) (Jackson Laboratory #025106), En1-Cre (Jackson Laboratory #007916), En1-Flpo, En1flox (Jackson Laboratory #007918), Foxp2-Flpo, Hoxb8-Cre, Piezo2-Cre (Jackson Laboratory #027719), RC.lsl.Sun1-sfGFP (INTACT) (Jackson Laboratory #021039), Tau.lsl.nLacZ (Jackson Laboratory #021162), and Tau.ds.DTR.
Wild animals	N/A
Reporting on sex	Both male and female mice were used in this study. Sex was not considered in the study design and was not expected to affect experimental outcomes.
Field-collected samples	N/A

Ethics oversight	All experiments and procedures were performed in accordance with National Institutes of Health (NIH) guidelines and approved by the Institutional Animal Care and Use Committee (IACUC) of St. Jude Children's Research Hospital.

Note that full information on the approval of the study protocol must also be provided in the manuscript.

Plants

Seed stocks	N/A
Novel plant genotypes	N/A
Authentication	N/A

Flow Cytometry

Plots

Confirm that:

- [x] The axis labels state the marker and fluorochrome used (e.g. CD4-FITC).
- [x] The axis scales are clearly visible. Include numbers along axes only for bottom left plot of group (a 'group' is an analysis of identical markers).
- [x] All plots are contour plots with outliers or pseudocolor plots.
- [ ] A numerical value for number of cells or percentage (with statistics) is provided.

Methodology

Sample preparation	Single nuclei were isolated from tissue as described in the methods.
Instrument	FACSAria Fusion Cell Sorter (BD Biosciences, San Jose, CA)
Software	BD FACSDiva software v8.0.1
Cell population abundance	GFP+ nuclei constituted about 1% of the input.
Gating strategy	GFP+ nuclei were selected based on log side scatter (SSC-A) versus log GFP fluorescence (GFP-A) and sorted in purity mode (sheath pressure 21 PSI, 100 um nozzle).

- [x] Tick this box to confirm that a figure exemplifying the gating strategy is provided in the Supplementary Information.
