# Supplementary material for: Transcriptomic analysis of spinal V1 interneurons informs their multifunctional role in motor output
Source: Nat Commun. 2026 Aug 10;17:9614. doi: 10.1038/s41467-026-76522-3 (PMC13554063; doi:10.1038/s41467-026-76522-3)
Supplement: Supplementary file 2 [file 41467_2026_76522_MOESM12_ESM.pdf]

nature portfolio

Peer Review File

Transcriptomic analysis of spinal V1 interneurons informs their multifunctional role in motor output

Corresponding Author: Dr Jay Bikoff

This file contains all reviewer reports in order by version, followed by all author rebuttals in order by version.

Attachments originally included by the reviewers as part of their assessment can be found at the end of this file.

Version 0:

Reviewer comments:

Reviewer #1

(Remarks to the Author)
In this manuscript, Trevisan and colleagues characterize the functional and cellular heterogeneity of the developmentally defined V1 population of spinal cord interneurons. The authors show that perturbing the En1 transcription factor which labels the V1 lineage in embryonic development alters spinal cord dependent rhythmic behaviors but not V1 dependent limb flexion phenotypes. The authors generate several high-quality single-cell transcriptomic datasets characterizing the neuron class diversity within V1 interneurons and show that En1 loss-of-function selectively reduces the abundance of one of identified V1 transcriptomic clusters. The latter suggests a potential cellular mechanism for how V1 population of spinal interneurons controls disparate motor functions. Although it remains unclear whether the loss of cluster 10 or molecular changes in other neuron types are responsible for the En1 loss-of-function derived selective motor deficit, the paper is a valuable advance for the field of spinal cord based motor control. Furthermore, this manuscript establishes clear avenues for future functional dissection of the role of V1 interneurons in motor output control. The experiments are sound and I only have minor comments on the analysis of the data:

• Figure 1: please increase the number of animals in En1 lesioned and En1 cKO groups (3-4 animals per experimental group is a significant under-sampling)
• Figure 2 - molecular diversity of V1 interneurons:
o Please provide a clear named nomenclature for the numbered clusters (e.g. V1-[most_selective_gene]. Referring to cluster numbers in subsequent publications will be cumbersome and confusing. Also, whether cluster numbering is the same or different in figures 2 and 6 is not immediately obvious.
• The authors dedicate two figures to reconciling their newly discovered V1 transcriptomic cell classes to a previous “clade” based nomenclature of V1 neurons. While useful from the perspective of pre-single-cell transcriptomic literature, this will be confusing going forward. The previously suggested clades are made up of clearly disparate transcriptomic clusters that only partially reflect transcriptomic homology of constituent neurons and are a fundamentally arbitrary nomenclature of V1 cells. I would suggest dedicating more space to molecular characterization of V1 transcriptomic neuron classes in main figures and relegating most of the “clade” to transcriptomic cell-class comparison to extended data figures.
• Figure 4: Please provide a dot or a violin plot of most selective markers for each V1 transcriptomic cluster (table S4 is not sufficient as the presented metrics do not clearly reveal specificity of marker genes). Although this analysis has been provided for transcription factors and ion channels, these may not include the most selective markers. This analysis would be valuable for the broader community to differentiate these neural clusters from the rest of spinal cord neuron types and to perform cell-type targeted manipulations.
• In the discussion section the authors speculate that the flexion could be regulated by the V1-Foxp2 clade but no data is presented. Is that due to perinatal death of the En1-Flp/Foxp2-Cre DTR lesioned animals or is there a different reason why motor rhythmicity is provided and flexion data is not for this genotype?
• Extended data figure 6 – top is cut off
• Data integration. Authors refer to integrating data using a Seurat implemented “anchor based workflow”. Many integration methods implemented in Seurat use anchoring. Please specify the method (CCA?).
• “cluster #13 was located at the far end of the dendrogram” – in most hierarchical clustering dendrograms the vertical distance as opposed to the horizontal distance/location is relevant to characterizing differences between entities. Instead of commenting on lateral distance, you can make the argument that based on vertical distance in hierarchical clustering cluster

13 is distinct from all other V1 transcriptomic neuron classes.
* I could not locate the Seurat/r code used in this manuscript at the Github site. Please add.

All in all, I enjoyed the manuscript and recommend it for publication with minor revisions.
Allan-Hermann Pool

(Remarks on code availability)
I could not locate the Seurat/R code used for this publication. The python code was there. Seurat based analysis should also be included.

Reviewer #2

(Remarks to the Author)
I co-reviewed this manuscript with one of the reviewers who provided the listed reports. This is part of the Nature Communications initiative to facilitate training in peer review and to provide appropriate recognition for Early Career Researchers who co-review manuscripts.

(Remarks on code availability)

Reviewer #3

(Remarks to the Author)
This study provides a comprehensive transcriptional classification of V1 neurons, offering valuable insights into their potential functional diversity.

One of the key stated achievements of the work is the identification of a V1 neuron subtype (V1Pou6f2) that is proposed to play a role in setting the rhythm of locomotor activity. However, this conclusion appears to rely primarily on correlative evidence, as no direct demonstration of how perturbing V1Pou6f2 affects locomotor rhythm is provided.

The argument seems to be that since knocking out En1 leads to changes in locomotor speed, and the most significant transcriptional consequence of En1 knockout is the selective loss of the Pou6f2 clade, this particular subclass of V1 neurons is implicated in rhythm regulation. While this is an intriguing hypothesis, alternative explanations remain possible, and no direct functional evidence is presented to substantiate this specific interpretation.

Stepping back, the manuscript is built on the premise that V1 neuron activity is responsible for both setting the locomotor rhythm and determining the degree of flexion in the system. However, in this work, hyperflexion is only tested in the context of tail suspension, raising questions about its physiological relevance during natural locomotion.

The impact of En1 loss on locomotor rhythm has been well documented, and previous studies have already demonstrated that En1 influences the number of synapses to motor neurons, making its effect on rhythm unsurprising.

One of the most compelling pieces of evidence for functional specialization within the V1 population, in my view, comes from the FoxP2 experiments. Setting aside the fact that such manipulation is not limited to the spinal cord, the finding that deletion of the FoxP2 V1 subpopulation does not affect locomotor rhythm provides an indication of potential functional divergence. Given the significance of this result, it is somewhat surprising that these findings are relegated to the supplementary figures. Additionally, it remains unclear whether ablation of the FoxP2 subpopulation has any effect on hyperflexion.

Overall, while the study provides valuable transcriptional insights and raises interesting hypotheses, some of its functional claims remain difficult to fully support with the current evidence. The impact of En1 loss on locomotor rhythm is not particularly novel given prior findings, and the proposed dichotomy of V1 functon(rhythm vs. hyperflexion) is challenging to interpret, given that hyperflexion is only tested under the rather artificial condition of tail suspension. Finally, the claim that the Pou6f2 subset selectively affects locomotor rhythm lacks direct functional validation.

If the authors were able to demonstrate that the Pou6f2 subpopulation is indeed responsible for the observed changes in rhythm—particularly in contrast to the FoxP2 subpopulation—this would represent a novel and compelling finding. However, as it stands, the study does not yet provide the level of functional evidence needed to fully support the proposed division of labour within the V1 population.

Major Points:
1. Provide functional evidence demonstrating that the Pou6f2 V1 subpopulation is responsible for the observed changes in rhythm.
2. Conduct a systematic evaluation of V1 ablation and conditional En1 KO during natural locomotion using kinematic tracking, with a particular focus on the degree of hyperflexion.
3. Define the spinal and supraspinal neurons co-expressing En1 and FoxP2 (e.g., using En1-CRE; FoxP2-FLP; Ai65D).
4. The validation of the non-overlapping nature of the identified clades was performed using immunohistochemistry. However, in situ hybridization (e.g., RNAscope) would be a more appropriate method for this purpose.
5. Test the effect of the ablation of the FoxP2 V1 subpopulation on hyperflexion (see 2)

Minor Point
1. Information on the origin of the FoxP2-FLP line is missing.

(Remarks on code availability)

Reviewer #4

(Remarks to the Author)
In the present manuscript, Trevisan and colleagues investigate the molecular and functional heterogeneity of the V1 spinal inhibitory interneurons using a combination of behavioural, electrophysiological and transcriptomics techniques. V1 interneurons were previously defined as Engrailed 1 (En1) positive, and their silencing/ablation was shown to decrease the frequency of locomotion and lead to hindlimb hyperflexion. In this study, mouse genetics, ventral root recordings and assessment of early postnatal intralimb coordination was used to investigate motor functions after loss of En1 expression. Moreover, a snRNA analysis was performed to investigate the molecular heterogeneity of V1 interneurons overtime, and to validate the fate of V1 clades (previously identified by Bikoff et al) during adulthood.

Below are some major technical concerns:

1) upon careful investigation, the snRNA dataset seems to present some serious issues that the authors need to address before the manuscript can be evaluated in its entirety:

- it is unexpected that neither the En1 nor the GFP mRNA can be consistently detected in the snRNA dataset. While it is true that En1 transcript is down regulated postnatally, En1 mRNA could be detected in human post-mortem tissue using snRNA as reported by Yadav et al 2023. Down regulation does not mean absence.
- To define V1 interneurons, the authors used a previously published embryonic dataset that they further validated, however when looking at transcript expression and co-expression using the provided online atlas https://v1interneurons.stjude.org/shinyApp/ high levels of glutamatergic markers can be observed – for example Slc1a1 and Slc1a2, both glutamate transporters. 30484 nuclei are positive for Slc1a1, 9455 for Slc1a2 and 5950 for both. The V1 interneurons are inhibitory, but it looks like half of the nuclei in this dataset express glutamate transporters. It is unclear where this expression may come from (other neurons or glia), but the excitatory nuclei need to be excluded from the analysis.
- When checking for potential co-expression of inhibitory and excitatory markers (which should not be present), it looks like many of the nuclei included in the dataset (27760 which is more than 1/4) express both. This may suggest more serious technical issues, due to ambient RNA or similar. Another possibility is that the online platform is not working properly. However, the authors should provide the raw data and apply more stringent QCs to include only En1/GFP positive neurons to the analysis.

2) Another major concern is that the tail suspension assay used to assess hyperflexion of the hindlimbs is not a locomotor assay, hence it cannot be easily compared to previously published work (Britz et al 2015, Allodi et al 2021). The authors claim absence of hyperflexion after En1 ablation, however changes in swing-stance phase during locomotion are not analysed. The involvement of sensory feedback (e.g., Ia afferents) in the tail suspension assay may also differ from the feedback involved in locomotion. Furthermore, it is unclear why early postnatal time points were chosen for behavioural analysis rather than adult ones, especially for the conditional KO mice. Hyperflexion linked to En1 silencing is well described in literature, so the authors need to provide further behavioural evidence. Moreover, from the Material and Methods description, it seems that the video frames included in the behavioural analysis were manually picked. This may lead to the introduction of biases in the analysis. The videos should be analysed in their entirety.

3) It is unclear why the authors do not use multiplexing analysis to validate that the clade-specific markers are mutually exclusive (Fig 3). Multiplexing analysis (detecting either proteins or mRNAs) is routinely used to validate transcriptomics data. The authors state that only 39 nuclei were found to co-express Pou6f2 and Sp8, however, the gene co-expression atlas seems to show differently (with 8568 nuclei co-expressing Foxp2 and Pou6f2). Again, this might be an issue related to the online tool, but since the main markers are only 5 (Foxp2, Pou6f2, Sp8, MafA and Rnf220) a multiplexing proteomics would be a more appropriate technique to use here.

(Remarks on code availability)

Version 1:

Reviewer comments:

Reviewer #1

(Remarks to the Author)
My previous comments were thoroughly addressed and I have no further criticism. Looking forward to seeing the final version of the manuscript out.

A-H.P.

(Remarks on code availability)
The code base is just a simple reproduction of the classic Leiden clustering workflow that is packaged into new functions. That's fine as is.

Reviewer #2

(Remarks to the Author)
I co-reviewed this manuscript with one of the reviewers who provided the listed reports. This is part of the Nature Communications initiative to facilitate training in peer review and to provide appropriate recognition for Early Career Researchers who co-review manuscripts.

(Remarks on code availability)

Reviewer #3

(Remarks to the Author)
The authors have undertaken a thorough revision that addresses all of my main comments. The presentation of the results is careful, honest, and appropriately balanced.

Although the study does not, at this stage, fully support the hypothesised role of Pou6f2, it provides valuable clarity and identifies important directions for future work.

The revised manuscript is valuable, and the language of the main text faithfully reflects the results, clearly distinguishing between findings that have been definitively demonstrated and those that will require further work to support the hypothesis. In my view, the authors have done as much as could reasonably be expected, and I am very happy to fully support publication.

I would also like to thank the authors for taking the time and dedicating the resources needed to address my comments. I appreciate how much effort has gone into this revision, and I am sure the field will be grateful for it.

(Remarks on code availability)

Reviewer #4

(Remarks to the Author)
The revised manuscript is improved and all my concerns were addressed. The new experiments and the revised analysis have clarified the previous issues concerning both the transcriptomic dataset and the behavioural assessment.

(Remarks on code availability)
The code is now available, however the README files are missing - this should be added before publication.

Open Access This Peer Review File is licensed under a Creative Commons Attribution 4.0 International License, which permits use, sharing, adaptation, distribution and reproduction in any medium or format, as long as you give appropriate credit to the original author(s) and the source, provide a link to the Creative Commons license, and indicate if changes were made.
In cases where reviewers are anonymous, credit should be given to 'Anonymous Referee' and the source.
The images or other third party material in this Peer Review File are included in the article's Creative Commons license, unless indicated otherwise in a credit line to the material. If material is not included in the article's Creative Commons license and your intended use is not permitted by statutory regulation or exceeds the permitted use, you will need to obtain permission directly from the copyright holder.
To view a copy of this license, visit https://creativecommons.org/licenses/by/4.0/

Response to Reviewers:

We are grateful for the thoughtful and constructive feedback provided by all four of the reviewers of our manuscript entitled "*Transcriptomic analysis of spinal V1 interneurons informs their multifunctional role in motor output*". We have carried out a thorough revision in which we've added new experiments and analyses and made extensive textual changes to address each of the comments. We feel these revisions have strengthened the manuscript and improved its rigor, clarity, and overall impact. A few of these improvements are highlighted below:

- We have reevaluated the original single-nucleus RNA-seq data and provide a more thorough explanation for each step of our quality control metrics and analyses, specifically addressing the major concerns raised regarding excitatory neuron and non-V1 neuron contamination. Furthermore, we performed additional validation experiments of our transcriptomic data using RNAScope, confirming the accuracy of our initial conclusions and supporting the overall utility of this dataset.
- We have provided additional data examining limb flexion during adult locomotion in En1 cKO and V1 ablated mice, demonstrating that the hyperflexion phenotype is only present in V1 ablated animals and not in mice lacking the En1 transcription factor.
- We have extensively reworked the text to clarify several technical and conceptual issues raised by the reviewers and discuss the inherent limitations of our findings.

As this study is also intended to serve as a resource for the community, it is essential that anyone can access all of our original code used for analysis. Several reviewers remarked that they were either unable to access the Seurat/R code or did not find the raw data. We have confirmed that the raw data is present and publicly available in Gene Expression Omnibus (GEO) with accession number GSE275595 (https://www.ncbi.nlm.nih.gov/geo/query/acc.cgi?acc=gse275595). The Seurat/R and Python code is present and available in Github at https://github.com/Bikoff-Lab/. If reviewers continue to have trouble accessing either the raw data or code we will provide an alternative means of sharing this information.

Below we cite the reviewer's comments in *italic* typeface and then provide our response in blue typeface.

Reviewer 1:

Remarks to the Author:
In this manuscript, Trevisan and colleagues characterize the functional and cellular heterogeneity of the developmentally defined V1 population of spinal cord interneurons. The authors show that perturbing the En1 transcription factor which labels the V1 lineage in embryonic development alters spinal cord dependent rhythmic behaviors but not V1 dependent limb flexion phenotypes. The authors generate several high-quality single-cell transcriptomic datasets characterizing the neuron class diversity within V1 interneurons and show that En1 loss-of-function selectively reduces the abundance of one of identified V1 transcriptomic clusters. The latter suggests a potential cellular mechanism for how V1 population of spinal interneurons controls disparate motor functions. Although it remains unclear whether the loss of cluster 10 or molecular changes in other neuron types are responsible for the En1 loss-of-function derived selective motor deficit, the paper is a valuable advance for the field of spinal cord based motor control. Furthermore, this manuscript establishes clear avenues for future functional dissection of the role of V1 interneurons

in motor output control. The experiments are sound and I only have minor comments on the analysis of the data:

All in all, I enjoyed the manuscript and recommend it for publication with minor revisions.
Allan-Hermann Pool

We thank the reviewer for their many valuable suggestions and acknowledging the importance of our study.

1. *Figure 1: please increase the number of animals in En1 lesioned and En1 cKO groups (3-4 animals per experimental group is a significant under-sampling)*

   As requested by the reviewer, we have increased the sample sizes for the frequency analysis of V1-ablated animals in Figure 1c (from N = 3 to N = 6), the ankle and knee angle analysis of V1-ablated animals in Figure 1e (from N = 3 to N = 7), and the frequency analysis of En1 cKO animals in Figure 1j (from N = 4 to N = 5). We performed all biological replicates in parallel with the corresponding littermate controls, which also increased in sample size when compared to the original submission.

2. *Figure 2 - molecular diversity of V1 interneurons:*
   o Please provide a clear named nomenclature for the numbered clusters (e.g. V1-[most_selective_gene]. Referring to cluster numbers in subsequent publications will be cumbersome and confusing. Also, whether cluster numbering is the same or different in figures 2 and 6 is not immediately obvious.

   The issue raised by the reviewer of how to best assign a clear nomenclature for each cluster is an important one and is something that we struggled with. Ideally, each corresponding cluster would align with a clear cell-type description. In the case of whole spinal cord transcriptomic studies, this might correspond to cluster names like "ependymal", "OPC", "microglia", "neuron", and so on. In our case, where we dissect a single cardinal interneuron population, distinctions will inevitably be more subtle and difficult to resolve. This is made even more challenging because it is unclear how different V1 subsets correspond to functionally relevant interneuron types, with the one exception being Renshaw cells. Taking the reviewer's concerns into account, balanced against our previous cladal nomenclature (which we still view as useful…see point #3), we have renamed the clusters according to their transcriptomic signatures, as described in Table S5 (reproduced below) and highlighted in Figure 3i and the revised text. As Renshaw cells are clearly identifiable in our dataset, this cluster is named $V1^{Renshaw}$ rather than by its molecular signature.

   The reviewer also raises a second important point regarding the clustering in Figures 2 and 6, each of which is based on completely independent datasets, with Figure 2 containing ~89,000 nuclei, and Figure 6 containing ~7,500 nuclei. These datasets were analyzed independently and found to exhibit a highly similar cluster assignment in which the basic molecular logic of each clade is preserved. However, they are not identical. In particular, whereas we identified 14 clusters in Figure 2, we were able to identify 11 clusters in Figure 6, reflecting the following differences: the $V1^{Sp8}$ clade was reduced from 3 to 2 clusters; the $V1^{Pou6f2}$ clade was reduced from 3 to 2 clusters, and the $V1^{Rnf220}$ clade is reduced from 2 to 1 cluster. We attribute this mainly to the over 10-fold greater sampling of V1 nuclei in the first dataset, which likely facilitated our ability to resolve transcriptional differences. To clarify this,

we have updated the text and included this information as part of Table S5. We thank the reviewer for prompting us to clarify these issues.

Figure 2 Cluster ID#	Molecular Designation
0	$V1^{\text{Foxp2\_Nr4a2}}$
1	$V1^{\text{Rnf220\_Maf}}$
2	$V1^{\text{Foxp2\_Trpc4(high)}}$
3	$V1^{\text{Pou6f2\_Grm8(high)}}$
4	$V1^{\text{Foxp2\_Foxp4\_Hhip}}$
5	$V1^{\text{Sp8\_St18/Prox1(low)}}$
6	$V1^{\text{Rnf220\_Bcl11a}}$
7	$V1^{\text{Sp8\_St18}}$
8	$V1^{\text{Renshaw}}$
9	$V1^{\text{Nr5a2 (Pou6f2-negative)}}$
10	$V1^{\text{Sp8\_Oc2}}$
11	$V1^{\text{Foxp2\_Foxp4\_Ebf3}}$
12	$V1^{\text{Pou6f2\_Pebp4}}$
13	$V1^{\text{Pou6f2\_Nr5a2}}$

Figure 6 Cluster ID#	Molecular Designation
0	$V1^{\text{Sp8\_St18}}$
1	$V1^{\text{Pou6f2\_Grm8(high)}}$
2	$V1^{\text{Foxp2\_Foxp4\_Ebf3}}$
3	$V1^{\text{Foxp2\_Foxp4\_Hhip}}$
4	$V1^{\text{Nr5a2 (Pou6f2-negative)}}$
5	$V1^{\text{Foxp2\_Nr4a2}}$
6	$V1^{\text{Rnf220}}$
7	$V1^{\text{Sp8\_St18/Prox1(low)}}$
8	$V1^{\text{Foxp2\_Trpc4(high)}}$
9	$V1^{\text{Renshaw}}$
10	$V1^{\text{Pou6f2\_Nr5a2}}$

Table S5. Cluster nomenclature for Figure 2 (left) and Figure 6 (right).

3. *The authors dedicate two figures to reconciling their newly discovered V1 transcriptomic cell classes to a previous "clade" based nomenclature of V1 neurons. While useful from the perspective of pre-single-cell transcriptomic literature, this will be confusing going forward. The previously suggested clades are made up of clearly disparate transcriptomic clusters that only partially reflect transcriptomic homology of constituent neurons and are a fundamentally arbitrary nomenclature of V1 cells. I would suggest dedicating more space to molecular characterization of V1 transcriptomic neuron classes in main figures and relegating most of the "clade" to transcriptomic cell-class comparison to extended data figures.*

As the reviewer notes, in previous work we categorized V1 interneurons into distinct clades ($V1^{\text{Foxp2}}$, $V1^{\text{MafA}}$, $V1^{\text{Pou6f2}}$, and $V1^{\text{Sp8}}$), which was based on the idea that differential expression of key transcription factors might be a useful way to categorize V1 interneurons. We found that these transcription factors were expressed in a mutually exclusive manner, using immunohistochemical analysis with antibodies rigorously validated against knockout mice. Additionally, these clades exhibited characteristic spatial distributions and were associated with distinct physiological signatures. In combination with other work showing that V1 clades exhibit different birthdates and extent of motor neuron innervation,[1] these data collectively provided strong evidence that V1 clades represent distinct cell types.

Notably, our current unbiased transcriptomic analysis reinforces these findings, with the four transcription factors associated with distinct clusters. Importantly, although Sp8 and MafA transcripts were not well detected, all four clade transcription factors are differentially expressed, with Foxp2 and Pou6f2 both well-detected and amongst the most selectively expressed transcripts in their respective clusters. Accordingly, we view the cladal nomenclature as biologically relevant and consistent with our transcriptomic data.

Nevertheless, as the reviewer correctly points out, our previous classification of V1 interneurons was not based on an unbiased molecular analysis. While not completely arbitrary

(there is good evidence that transcription factor expression is a key determinant of cell identity), we agree that naming each cluster according to the most selectively-expressed genes will be helpful for the field. Thus, we have revised our cluster assignment to reflect a combination of relevant clade transcription factors (where appropriate) and specific molecular signatures, as noted in Point #2.

4. *Figure 4: Please provide a dot or a violin plot of most selective markers for each V1 transcriptomic cluster (table S4 is not sufficient as the presented metrics do not clearly reveal specificity of marker genes). Although this analysis has been provided for transcription factors and ion channels, these may not include the most selective markers. This analysis would be valuable for the broader community to differentiate these neural clusters from the rest of spinal cord neuron types and to perform cell-type targeted manipulations.*

   We have now included a dot plot that shows the most selective markers for each V1 transcriptomic cluster and also highlights the revised molecular nomenclature for these clusters (see Figure 3i). We have chosen to add this data to Figure 3 rather than Figure 4, as it naturally follows from our analysis of V1 clades and provides a more accurate summary of the state of V1 interneuron diversity. We thank the reviewer for this suggestion, which we agree will be helpful to the community.

5. *In the discussion section the authors speculate that the flexion could be regulated by the V1-Foxp2 clade but no data is presented. Is that due to perinatal death of the En1-Flp/Foxp2-Cre DTR lesioned animals or is there a different reason why motor rhythmicity is provided and flexion data is not for this genotype?*

   This is an important point that was also raised by Reviewer 3. Because at least some classical group Ia reciprocal inhibitory interneurons are contained within the $V1^{Foxp2}$ population,[1, 2] this suggests a potential role for these neurons in influencing flexion/extension movements. The genetic strategy we used to ablate $V1^{Foxp2}$ interneurons relies on *En1*^{Cre}*; Foxp2*^{Flpo} mice crossed to *Tau.ds.DTR* animals, with diphtheria toxin (DT) administered at birth (P0). This strategy does not restrict ablation to the spinal cord and unfortunately also kills $En1^{+}$ and $Foxp2^{+}$ neurons distributed throughout the brainstem (see also Reviewer 3, Point #3, which demonstrates the extensive overlap in the brain). Consequently, although we are able to perform ventral root recordings to assess fictive locomotion in isolated spinal cords from these animals 2 days post-DT administration (Supplementary Figure 1j), the animals at this age are near death, making it difficult to properly assess flexion/extension movements, just as the reviewer notes. We nevertheless attempted to assess flexion/extension movements in the tail suspension assay. This did not reveal any difference compared to control animals (see Reviewer Figure 3), but it is difficult to interpret whether this is truly due to $V1^{Foxp2}$ neurons having no role in flexion/extension, or because of confounding effects from either the health of the animals or perturbed activity of descending motor commands from the brainstem. We have now included a more detailed discussion of the potential role for $V1^{Foxp2}$ interneurons in controlling limb flexion that takes into account the limitations of our experiments.

6. *Extended data figure 6 – top is cut off*

   We apologize for this formatting error and have corrected it in the current version (Supplementary Fig. 6).

7. *Data integration. Authors refer to integrating data using a Seurat implemented "anchor based workflow". Many integration methods implemented in Seurat use anchoring. Please specify the method (CCA?).*

   Indeed, we used the Seurat-CCA (Canonical Correlation Analysis) method to integrate multiple datasets. This is now explicitly stated in the methods section.

8. *"cluster #13 was located at the far end of the dendrogram" – in most hierarchical clustering dendrograms the vertical distance as opposed to the horizontal distance/location is relevant to characterizing differences between entities. Instead of commenting on lateral distance, you can make the argument that based on vertical distance in hierarchical clustering cluster 13 is distinct from all other V1 transcriptomic neuron classes.*

   The reviewer is entirely correct with this point. We have corrected the text and thank the reviewer for catching this mistake.

9. *I could not locate the Seurat/r code used in this manuscript at the Github site. Please add.*

   We apologize that the reviewer could not locate our Seurat and R code at Github. We have confirmed the code is publicly available through the Bikoff-Lab Github website, available at https://github.com/Bikoff-Lab/. This includes:
   (1) Atlas analysis: https://github.com/Bikoff-Lab/V1_interneuron_snRNAseq_Age_Atlas
   (2) En1 KO analysis: https://github.com/Bikoff-Lab/V1_KO_analysis

   If the reviewer continues to have any difficulty finding or accessing the code, please let us know and we will provide an alternate method to ensure it is available to the community.

Reviewer #2:

I co-reviewed this manuscript with one of the reviewers who provided the listed reports. This is part of the Nature Communications initiative to facilitate training in peer review and to provide appropriate recognition for Early Career Researchers who co-review manuscripts.

We thank the reviewer for contributing to the review of our manuscript. Please see our detailed point-by-point responses to your and the other reviewers' comments.

Reviewer #3:

Remarks to the Author:
This study provides a comprehensive transcriptional classification of V1 neurons, offering valuable insights into their potential functional diversity.

One of the key stated achievements of the work is the identification of a V1 neuron subtype (V1Pou6f2) that is proposed to play a role in setting the rhythm of locomotor activity. However,

this conclusion appears to rely primarily on correlative evidence, as no direct demonstration of how perturbing V1Pou6f2 affects locomotor rhythm is provided.

The argument seems to be that since knocking out En1 leads to changes in locomotor speed, and the most significant transcriptional consequence of En1 knockout is the selective loss of the Pou6f2 clade, this particular subclass of V1 neurons is implicated in rhythm regulation. While this is an intriguing hypothesis, alternative explanations remain possible, and no direct functional evidence is presented to substantiate this specific interpretation.

Stepping back, the manuscript is built on the premise that V1 neuron activity is responsible for both setting the locomotor rhythm and determining the degree of flexion in the system. However, in this work, hyperflexion is only tested in the context of tail suspension, raising questions about its physiological relevance during natural locomotion.

The impact of En1 loss on locomotor rhythm has been well documented, and previous studies have already demonstrated that En1 influences the number of synapses to motor neurons, making its effect on rhythm unsurprising.

One of the most compelling pieces of evidence for functional specialization within the V1 population, in my view, comes from the FoxP2 experiments. Setting aside the fact that such manipulation is not limited to the spinal cord, the finding that deletion of the FoxP2 V1 subpopulation does not affect locomotor rhythm provides an indication of potential functional divergence. Given the significance of this result, it is somewhat surprising that these findings are relegated to the supplementary figures. Additionally, it remains unclear whether ablation of the FoxP2 subpopulation has any effect on hyperflexion.

Overall, while the study provides valuable transcriptional insights and raises interesting hypotheses, some of its functional claims remain difficult to fully support with the current evidence. The impact of En1 loss on locomotor rhythm is not particularly novel given prior findings, and the proposed dichotomy of V1 functon(rhythm vs. hyperflexion) is challenging to interpret, given that hyperflexion is only tested under the rather artificial condition of tail suspension. Finally, the claim that the Pou6f2 subset selectively affects locomotor rhythm lacks direct functional validation.

If the authors were able to demonstrate that the Pou6f2 subpopulation is indeed responsible for the observed changes in rhythm—particularly in contrast to the FoxP2 subpopulation—this would represent a novel and compelling finding. However, as it stands, the study does not yet provide the level of functional evidence needed to fully support the proposed division of labour within the V1 population.

We thank the reviewer for the many thoughtful comments on our manuscript. Below we address each of the central concerns raised by the reviewer. In most cases, each concern directly corresponds to one of the reviewer's major points, as noted.

1. *One of the key stated achievements of the work is the identification of a V1 neuron subtype (V1Pou6f2) that is proposed to play a role in setting the rhythm of locomotor activity. However, this conclusion appears to rely primarily on correlative evidence, as no direct demonstration of how perturbing V1Pou6f2 affects locomotor rhythm is provided. The argument seems to be that since knocking out En1 leads to changes in locomotor speed, and the most significant*

transcriptional consequence of En1 knockout is the selective loss of the Pou6f2 clade, this particular subclass of V1 neurons is implicated in rhythm regulation. While this is an intriguing hypothesis, alternative explanations remain possible, and no direct functional evidence is presented to substantiate this specific interpretation.

Major Point 1:
Provide functional evidence demonstrating that the Pou6f2 V1 subpopulation is responsible for the observed changes in rhythm.

The Reviewer appropriately highlights the question of whether V1^{Pou6f2} neurons are responsible for the slowed locomotor rhythm observed in *En1* mutant mice, which gets to the heart of a central challenge we have faced in this manuscript. Our manuscript has two main goals: first, to provide a detailed transcriptional classification of V1 interneurons, and second, to provide insight into how and why V1 interneurons play multifunctional roles in motor control. Beyond our broad transcriptomic analysis, we found that *En1* mutants exhibit a slowed locomotor rhythm but do not show limb hyperflexion. At a cellular level, loss of *En1* selectively reduces the number of V1^{Pou6f2_Nr5a2} interneurons, providing a plausible explanation for the observed changes in rhythm. Because this link is correlative, we were extremely interested in directly testing whether V1^{Pou6f2} neurons influence locomotor rhythm speed using a previously generated *Pou6f2^{Flpo}* mouse, which we have used in earlier anatomical studies.[3] In initial experiments where we attempted to ablate V1^{Pou6f2} neurons in *En1^{Cre}; Pou6f2^{Flpo}; Tau.ds.DTR* mice, we did not observe any effect. Upon further analysis of this line, it became clear that it targets only a small fraction (~25%) of all Pou6f2 neurons (Reviewer Figure 1), making it impossible to interpret the experiment – the absence of a phenotype may be due to V1^{Pou6f2} neurons not being involved, or could be because we are targeting such a small proportion of V1^{Pou6f2} interneurons. While we have made every effort to answer this question, we fully acknowledge that the lack of functional evidence directly linking the V1^{Pou6f2} subset to the observed changes in rhythm is an important limitation of our studies. The absence of a phenotype does not, however, negate any of the other important findings in the manuscript, and instead calls for the development of future genetic reagents to properly address this question. Taking the reviewer's comments to heart, we have revised the text to expand upon this limitation of our study.

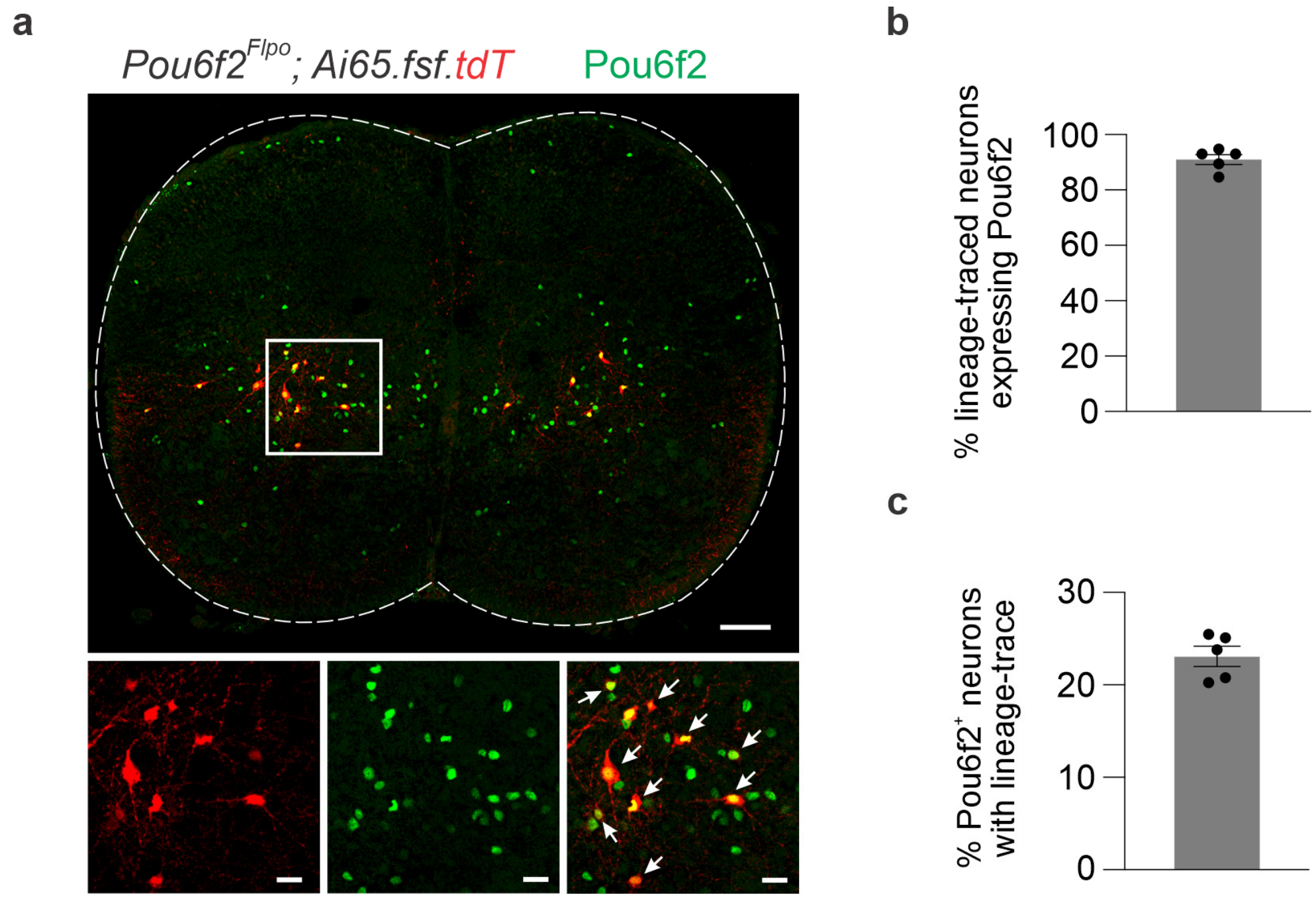

Figure 1. Specificity and efficiency of the *Pou6f2^{Flpo}* line.
(a) Immunohistochemical analysis of a P1 lumbar spinal cord from *Pou6f2^{Flpo}*; *Ai65.fsf.tdT* (Flp-dependent tdT) mice, showing lineage-traced Pou6f2 interneurons (red) and Pou6f2 expression (green). While almost all lineage-traced neurons still express Pou6f2 at this developmental timepoint (arrows), many Pou6f2-expressing neurons were not lineage-traced. Scale bars = 100 µm (top) or 20 µm (bottom). **(b)** Percentage of lineage-traced neurons that still express Pou6f2 (specificity of 91.1 ± 1.8%, mean ± SEM, n = 5 animals). **(c)** Percentage of Pou6f2-expressing neurons that were lineage-traced in *Pou6f2^{Flpo}; Ai65.fsf.tdT* mice (efficiency of 23.1 ± 1.1%, mean ± SEM, n = 5 animals).

2. *Stepping back, the manuscript is built on the premise that V1 neuron activity is responsible for both setting the locomotor rhythm and determining the degree of flexion in the system. However, in this work, hyperflexion is only tested in the context of tail suspension, raising questions about its physiological relevance during natural locomotion.*

   Major Point 2:
   Conduct a systematic evaluation of V1 ablation and conditional En1 KO during natural locomotion using kinematic tracking, with a particular focus on the degree of hyperflexion.

   As requested by the reviewer, we have performed additional experiments and now include detailed kinematic analyses of locomotor behavior in adult V1 ablated and En1 cKO mice. These experiments revealed that V1 ablation results in hyperflexion of the ankle during the swing phase of the step cycle, exemplified by the reduced angle of the ankle and the increased height of the hindpaw that is a consequence of the hyperflexed state (see Figure 1f-h and Supplementary Figure 1d). In contrast, kinematic analysis of En1 cKO animals showed that these animals do not exhibit hyperflexion, with limb position characterized instead by a slight hypoflexion (see Figure 1n-o and Supplementary Figure 1e). Taken together, these data reinforce the central observation that the hyperflexion phenotype resulting from loss of V1 interneurons is not present upon deletion of En1. We thank the reviewer for suggesting this experiment, which provides compelling evidence that our findings are physiologically relevant during natural locomotion.

3. *The impact of En1 loss on locomotor rhythm has been well documented, and previous studies have already demonstrated that En1 influences the number of synapses to motor neurons, making its effect on rhythm unsurprising.*

   This comment raises important considerations when thinking about potential mechanistic explanations for how loss of *En1* affects the speed of locomotor rhythm. While the slowed locomotor rhythm resulting from loss of *En1* is indeed well documented,[4] what accounts for this phenotype remains unclear. Loss of *En1* has previously been shown to lead to connectivity defects, including a decrease in Renshaw innervation of motor neurons as mentioned by the reviewer.[5, 6] While it is reasonable to think this might affect the speed of rhythmic locomotor output, multiple experiments argue that Renshaw cells are not responsible for this phenotype. For example, mecamylamine administration to block Renshaw activation by motor axon collaterals does not affect speed of locomotor rhythm.[7] Moreover, deletion of VIAAT (the transporter essential for loading GABA and glycine into synaptic vesicles) in Renshaw cells to eliminate their ability to inhibit motor neurons similarly found no effect on the speed of rhythmic locomotor output during fictive locomotion.[8] This suggests other V1 subsets are responsible for controlling locomotor rhythm, though it remains possible that a different connectivity issue unrelated to our finding on $V1^{Pou6f2}$ neurons may contribute. We have revised the discussion section to clarify this issue and thank the reviewer for prompting us to address this in more detail.

4. *Major Point 3:*
   Define the spinal and supraspinal neurons co-expressing En1 and FoxP2 (e.g., using En1-CRE; FoxP2-FLP; Ai65D).

   This is an important point that is part of the larger question of what role $V1^{Foxp2}$ interneurons play in motor output. The fundamental technical challenge for us in assessing the role of $V1^{Foxp2}$ interneurons in motor output stems from the issue recognized by the reviewer – namely, that *En1* and *Foxp2* are expressed in overlapping regions of the brain. Indeed, before initiating our experiments we were curious to see whether Foxp2 protein was expressed in *En1* lineage-traced neurons, which we assessed by crossing $En1^{Cre}$ with a Cre-dependent reporter expressing membrane-bound GFP and nuclear β-galactosidase (*Tau.lsl.mGFP-IRES-nLacZ*). Though slightly different than the $En1^{Cre}$*;* $Foxp2^{Flpo}$*; Ai65D* experiment suggested by the reviewer (which would presumably show even greater overlap, given that nature of the Foxp2 lineage-trace), our experiment demonstrated extensive overlap in numerous brain regions (Reviewer Figure 2). In a P0 mouse brain, we found overlap in developing diencephalic structures like the basal plate (Box 1), in midbrain regions like the inferior colliculus (Box 2), in the cerebellum (Box 3), and in medullary neurons in the caudal hindbrain (Box 4).

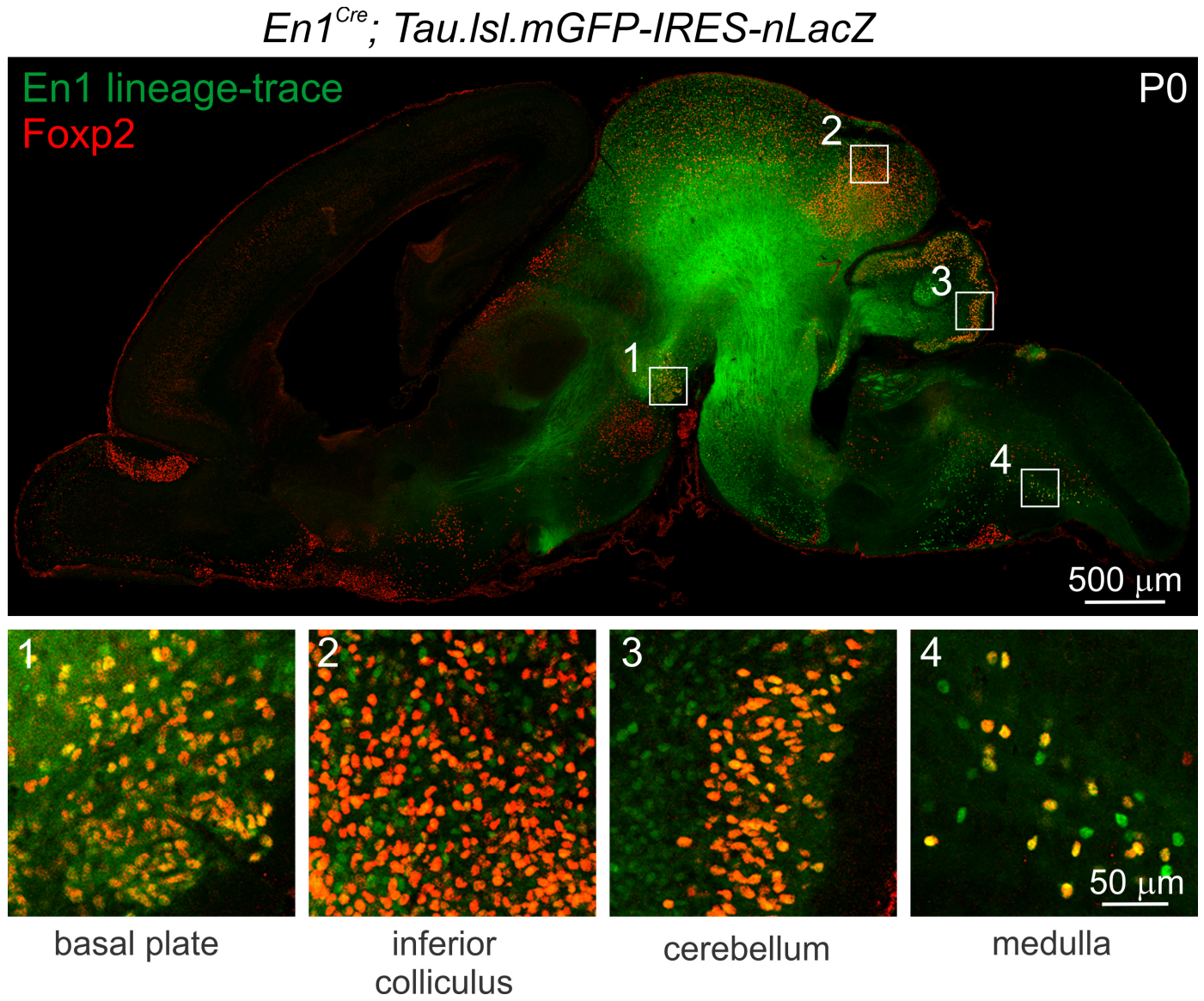

Figure 2. Overlapping expression of *En1* and *Foxp2* in the neonatal mouse brain. Immunohistochemical analysis of *En1* lineage-traced neurons (green, representing the combined signal from mGFP and IHC against nuclear β-gal) and Foxp2 protein (red) in a sagittal P0 brain from *En1^{Cre}; Tau.lsl.mGFP-IRES-nLacZ* mice. Boxed regions highlight brain regions with extensive overlap, including the basal plate (1), the inferior colliculus (2), the cerebellum (3), and scattered neurons in the medulla (4).

Thus, it is clear that intersectional genetic strategies relying on *En1^{Cre}, Foxp2^{Flpo},* and dual recombinase-dependent lines like *Tau.ds.DTR* are not restricted to the spinal cord and perturb a substantial proportion of neurons in the brain.

Regarding the nature of spinal neurons that coexpress *En1* and *Foxp2* (V1^{Foxp2} interneurons), these neurons have been described previously. To briefly summarize, when *Foxp2*^{Flpo} is combined with *En1^{Cre}* and the *Ai65D* intersectional reporter line, we found that it lineage-traces over half of all V1 interneurons.[9] This observation has also been replicated in more recent studies from the Alvarez lab.[1]

5. *Major Point 4:*
   The validation of the non-overlapping nature of the identified clades was performed using immunohistochemistry. However, in situ hybridization (e.g., RNAscope) would be a more appropriate method for this purpose.

   We agree that an “apples to apples” comparison, in this case validating our transcriptomic data with an independent mRNA detection method (e.g. RNAScope) is both entirely appropriate and would strengthen the manuscript. Accordingly, we performed multiplexed RNAScope fluorescence *in situ* hybridization to assess mRNA transcripts for all five clade markers (*Calb1, Foxp2, Pou6f2, Rnf220,* and *Sp8*) within lineage-traced V1 interneurons. For technical reasons, we analyzed four marker genes simultaneously and therefore performed three different rounds of RNAScope to cover all pairwise comparisons. This data, presented in Supplementary Figure 4, demonstrates that all five markers are expressed within V1 interneurons, and additionally shows they are expressed in a mutually exclusive manner, consistent with our single-nucleus transcriptomic dataset. We thank the reviewer for suggesting this experiment, as it provides additional confidence in the validity of our

transcriptomic data and analysis, which is further supported by the complementary immunohistochemical experiments.

6. *One of the most compelling pieces of evidence for functional specialization within the V1 population, in my view, comes from the FoxP2 experiments. Setting aside the fact that such manipulation is not limited to the spinal cord, the finding that deletion of the FoxP2 V1 subpopulation does not affect locomotor rhythm provides an indication of potential functional divergence. Given the significance of this result, it is somewhat surprising that these findings are relegated to the supplementary figures. Additionally, it remains unclear whether ablation of the FoxP2 subpopulation has any effect on hyperflexion."*

   Major Point 5:
   Test the effect of the ablation of the FoxP2 V1 subpopulation on hyperflexion (see 2)

   Despite overlapping expression in the brain, we attempted to perform the tail suspension assay in *En1^{Cre}; Foxp2^{Flpo}; Tau.ds.DTR* to determine if they might exhibit a hyperflexion phenotype. This was motivated in part by previous data showing that the V1^{Foxp2} population contains at least some classical group Ia reciprocal inhibitory interneurons thought to mediate flexion/extension movements.[1] Unfortunately, just two days after administering diphtheria toxin to *En1^{Cre}; Foxp2^{Flpo}; Tau.ds.DTR* mice, animals exhibit severely impaired health and invariably die either that day or within the following 24 hours. We nevertheless attempted to perform kinematic analysis in these animals but did not observe any hyperflexion phenotype during tail suspension (Reviewer Figure 3). However, it is difficult to interpret whether this is truly due to V1^{Foxp2} neurons having no role in controlling flexion movements, or because of confounding effects from either the health of the animals or perturbed activity of descending motor commands from the brainstem. We have now included a more detailed description of the various arguments for and against a direct role for V1^{Foxp2} interneurons influencing limb flexion that takes into account the limitations of our experiments.

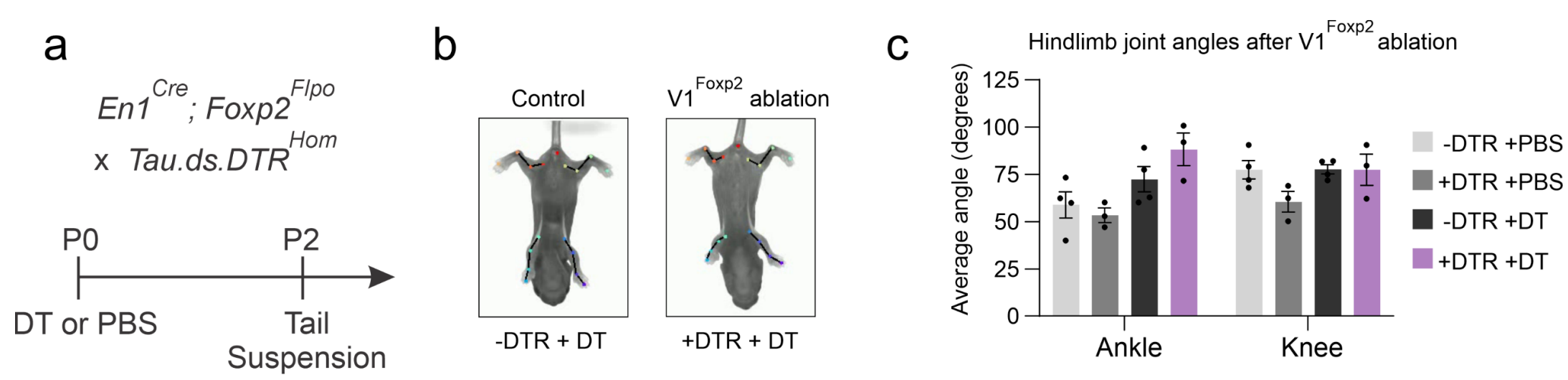

Figure 3. Absence of hyperflexion phenotype during tail suspension in V1^{Foxp2} ablated mice. (a) Schematic of the V1^{Foxp2} ablation paradigm, with tail suspension performed 2 days post-DT administration due to lethality. (b) Control (left) or V1^{Foxp2} ablated (right) mice during tail suspension, showing normal flexion of the hindlimbs. (c) Quantification of the average angle of the ankle and knee joints shows no hyperflexion under V1^{Foxp2} ablated conditions (purple).

   We also appreciate the reviewer's suggestion to move the V1^{Foxp2} fictive locomotor data to the main Figure 1. Given that the central point of Figure 1 is to show that loss of spinal *En1* results in only a subset of the V1 ablation motor phenotypes, and that the data on V1^{Foxp2} neurons is limited to fictive locomotion due to the confounding factors in the V1^{Foxp2} tail suspension experiment (described above), we have left this data in the Supplementary section. We are happy to add it to Figure 1 if the reviewer continues to feel strongly about this.

7. *Minor Point*
   Information on the origin of the FoxP2-FLP line is missing.

   We apologize for this omission and have added the reference for the *Foxp2^{Flpo}* line (described in Bikoff et al., 2016) in the Methods section.

Reviewer #4:

Remarks to the Author:

In the present manuscript, Trevisan and colleagues investigate the molecular and functional heterogeneity of the V1 spinal inhibitory interneurons using a combination of behavioural, electrophysiological and transcriptomics techniques. V1 interneurons were previously defined as Engrailed 1 (En1) positive, and their silencing/ablation was shown to decrease the frequency of locomotion and lead to hindlimb hyperflexion. In this study, mouse genetics, ventral root recordings and assessment of early postnatal intralimb coordination was used to investigate motor functions after loss of En1 expression. Moreover, a snRNA analysis was performed to investigate the molecular heterogeneity of V1 interneurons overtime, and to validate the fate of V1 clades (previously identified by Bikoff et al) during adulthood.

Below are some major technical concerns:

1. *upon careful investigation, the snRNA dataset seems to present some serious issues that the authors need to address before the manuscript can be evaluated in its entirety:*

   We apologize for the lack of clarity regarding aspects of our V1 transcriptomic dataset, which understandably led to several points of confusion. Below we address each of the specific concerns raised by the reviewer, followed by an additional discussion in section (c) describing the process through which we identify V1 nuclei with high confidence.

   (a) *it is unexpected that neither the En1 nor the GFP mRNA can be consistently detected in the snRNA dataset. While it is true that En1 transcript is down regulated postnatally, En1 mRNA could be detected in human post-mortem tissue using snRNA as reported by Yadav et al 2023. Down regulation does not mean absence*.

   The reviewer is entirely correct on the point that we should not have described the expression of *En1* and *GFP* in our dataset as being absent. We do, in fact, detect some transcript for both *En1* and *GFP*. However, the levels of detection for both genes were sufficiently low that they could not be used as a reliable indicator of V1 identity, prompting us to develop a more rigorous analysis pipeline, described in the Methods section as well as in part (c) below. For example, out of our total postnatal dataset of 89,494 V1-assigned nuclei, we detected En1 transcripts in only 4.8% of them (4,255 nuclei). Similarly, we detected GFP transcripts in only 1% of our V1 nuclei despite clear visual evidence that our flow cytometry effectively enriched for GFP^{+} nuclei (see Supplementary Figure 2a-c). This type of problem is often observed in single-cell and single-nucleus datasets, and while the precise reasons are unknown, it likely in part reflects the low capture rate of less

abundant transcripts using this technology – particularly in single-nucleus experiments lacking cytoplasmic transcripts.[10] We have edited the text to reflect these considerations and thank the reviewer for prompting us to clarify this.

(b) *To define V1 interneurons, the authors used a previously published embryonic dataset that they further validated, however when looking at transcript expression and co-expression using the provided online atlas https://v1interneurons.stjude.org/shinyApp/ high levels of glutamatergic markers can be observed – for example Slc1a1 and Slc1a2, both glutamate transporters. 30484 nuclei are positive for Slc1a1, 9455 for Slc1a2 and 5950 for both. The V1 interneurons are inhibitory, but it looks like half of the nuclei in this dataset express glutamate transporters. It is unclear where this expression may come from (other neurons or glia), but the excitatory nuclei need to be excluded from the analysis.*

This is an important point that requires clarification. V1 interneurons are indeed inhibitory.[6] As our single-nucleus transcriptomic data shows, nuclei assigned a V1 identity express the key inhibitory interneuron markers *Slc32a1*, which encodes the vesicular GABA transporter VGAT, and *Slc6a5*, which encodes the glycine transporter GlyT2 (Reviewer Figure 4a). The key marker used to identify excitatory interneurons in the spinal cord is *Slc17a6*, which encodes the vesicular glutamate transporter VGLUT2.[11] Importantly, this is almost entirely absent from our V1 nuclei (Reviewer Figure 4b). The few nuclei that do show *Slc17a6* transcript expression can be explained by our use of a negative selection pipeline in which excitatory neurons were identified and eliminated on a per cluster (not per nucleus) basis, thus retaining isolated cases of V1 nuclei with *Slc17a6*. Taken together, this data demonstrates the nuclei in our transcriptomic dataset are inhibitory.

Regarding the specific question of the glutamate transporters *Slc1a1* and *Slc1a2*, it is important to recognize that these are *not* specific markers for excitatory neurons. Instead, they are generally involved in uptake of glutamate, which serves a variety of functions. Indeed, they are expressed in many other types of cells, including inhibitory neurons.[12] For example, *Slc1a1* (called EEAT3) is thought to be broadly expressed in the brain, including in inhibitory interneurons where it facilitates uptake of glutamate, a precursor for GABA synthesis.[13] *Slc1a2* (called EEAT2) is predominately expressed in astrocytes, where it is responsible for the majority of glutamate uptake under physiological conditions (although it can also be expressed in neurons).[14] As the reviewer notes, these two genes are indeed expressed in our V1 nuclei (Reviewer Figure 4c), but this does not in any way indicate they are excitatory. Rather, an analysis of key excitatory and inhibitory markers clearly demonstrates the V1 nuclei in our dataset are inhibitory. We thank the reviewer for prompting us to clarify this issue.

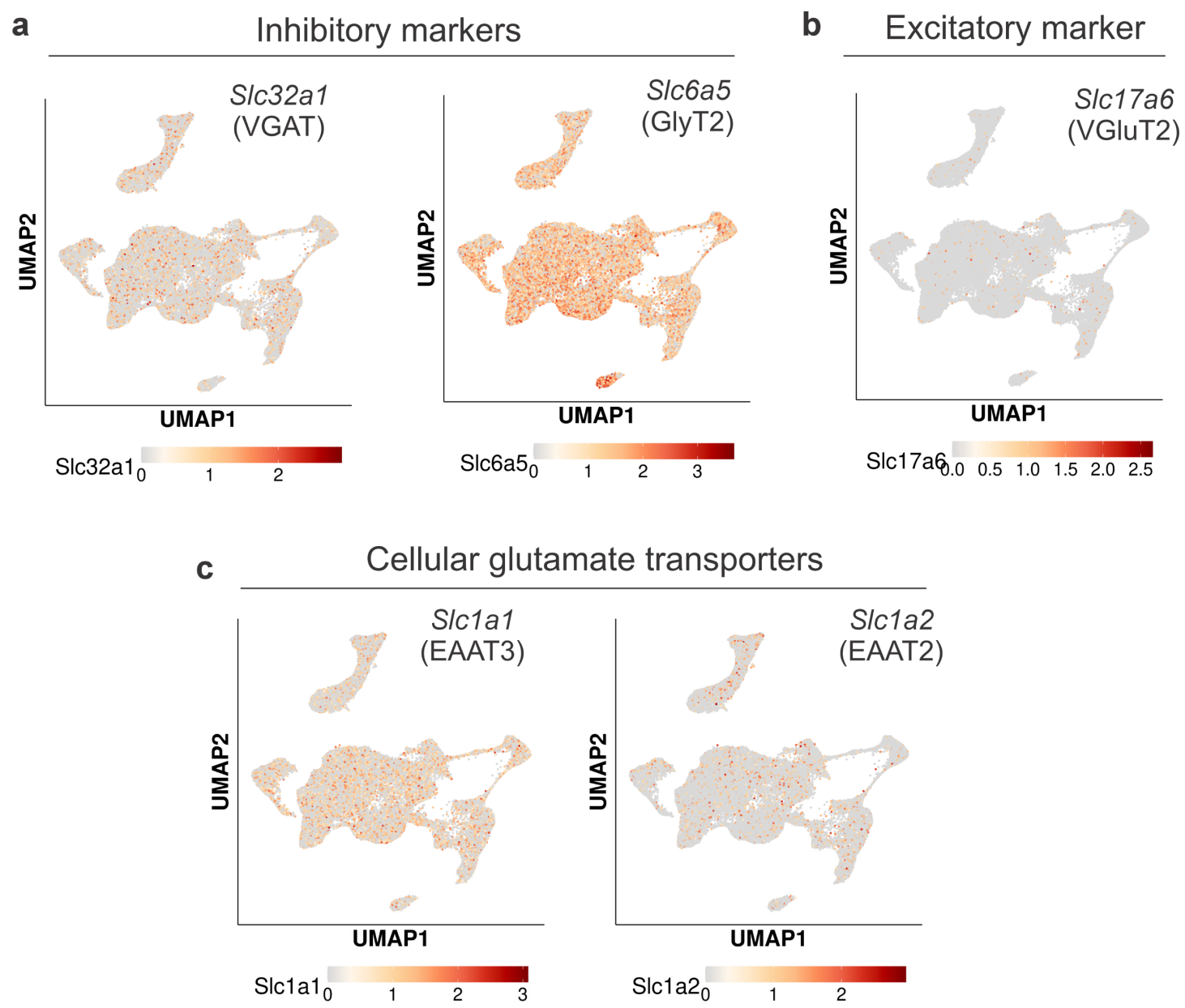

Figure 4. UMAP of key inhibitory and excitatory markers and cellular glutamate transporters in nuclei assigned a V1 interneuron identity.
(a) UMAPs showing expression of the key inhibitory markers *Slc32a1* (VGAT, left) and *Slc6a5* (GlyT2, right). (b) UMAP showing lack of expression of *Slc17a6* (VGluT2). (c) UMAP showing expression of glutamate transporters *Slc1a1* (EAAT3) and *Slc1a2* (EAAT2).

(c) *When checking for potential co-expression of inhibitory and excitatory markers (which should not be present), it looks like many of the nuclei included in the dataset (27760 which is more than 1/4) express both. This may suggest more serious technical issues, due to ambient RNA or similar. Another possibility is that the online platform is not working properly. However, the authors should provide the raw data and apply more stringent QCs to include only En1/GFP positive neurons to the analysis.*"

The reviewer raises an important question about the general issue of extent of co-expression of different genes. Regarding the specific issue of co-expression of inhibitory and excitatory markers, as described above we note that there is not substantial overlap of inhibitory and excitatory markers in our V1 nuclei. We also note we have provided raw data in its entirety, which is accessible at Gene Expression Omnibus (GEO) with accession number GSE275595. Given the general concerns of the reviewer that center on whether we are actually looking at V1 interneuron nuclei, it is important to highlight the many steps we took to rigorously ensure we only included V1 interneurons in our final annotated snRNA-seq dataset.

1. We confirmed that our flow cytometry experiments successfully enriched GFP⁺ nuclei. We used a combination of fluorescence activated nuclei sorting and visual confirmation by microscopy (both pre- and post- sorting) to demonstrate enrichment of GFP⁺ nuclei, as shown in Supplementary Figure 2a-c.
2. We performed extensive quality control to ensure robustness of the dataset. As ambient RNA contamination was detected, we compared two widely used correction approaches, SoupX and CellBender. Observing only nominal differences in

downstream results, we proceeded with SoupX-corrected counts for all subsequent analyses. To remove low-quality droplets and potential multiplets, we applied standard filtering criteria, including minimum thresholds for unique molecular identifiers (≥1,000 UMIs) and gene detection (≥10 cells per gene), exclusion of nuclei with elevated mitochondrial content (>2.5%), and removal of outlier cells based on total UMI counts using ±3 median absolute deviations (MADs).

3. We performed multiple rounds of negative selection to remove non-V1 nuclei that remained after flow cytometry enrichment. Following quality control, we used module scores to eliminate non-V1 nuclei that remained in our dataset. Supplemental Table 2 lists the marker genes used to identify cell types. Importantly, we did not use the presence of a single read of a given gene to eliminate nuclei. Rather, a conglomeration of genes was used to eliminate contaminating nuclei on a per-cluster basis, whereby the module score was calculated for each nucleus individually, and the median of the module score for the entire cluster of nuclei was used as the criteria for negative selection. Critically, this method bolsters against high rates of dropout, noise, and ambient RNA. Excitatory neurons and other non-V1 cell types including different glia and endothelial cells were identified with this method as shown in Supplementary Figure 2e-h and removed from our dataset.
4. We performed positive selection aided by previously published single-cell reference data from the literature. To further validate that the nuclei remaining after quality control and negative selection were indeed V1 interneurons, we used the whole spinal cord, embryonic single cell RNA-seq dataset from Delile et al.[15] as our reference because of its robust detection of traditional cardinal class markers of spinal interneurons. We isolated En1 lineage-traced GFP$^+$ nuclei from embryonic spinal cords and used Seurat's built-in Label Transfer method to compare our V1-enriched snRNA-seq dataset with the Delile dataset. This analysis is shown in Supplementary Figure 2i-n. We then used this embryonic snRNA-deq dataset as a bridge to our postnatal V1 nuclei and found that nearly all of the nuclei remaining after negative selection received a V1 prediction score above 90%.
5. Our curated V1 snRNA-seq dataset is consistent with a V1 identity. Beyond their inhibitory identity (addressed above), in a prior study we identified 19 transcription factors that were expressed in V1 interneurons on the basis of immunohistochemical analysis.[9] Because of this earlier characterization, we had knowledge of the approximate proportions and overlap of these genes. We found that our snRNA-seq results aligned with expectations of expression of these transcription factors (Figure 2g, Supplementary Figure 5), consistent with our independent immunohistochemical ground-truth data.
6. Finally, we provide multiple lines of evidence in which we validated key aspects of our snRNA-seq results using immunohistochemistry, genetic lineage tracing, and fluorescence *in situ* hybridization. At the protein level, we used immunohistochemistry (Figure 3) to confirm expression of numerous transcription factors expressed by the V1 interneurons. We also validated the presence of *Piezo2* expression in V1 interneurons using a *Piezo2^{Cre}*, *En1^{Flpo}*, and *Ai65D* lineage trace, confirming our single-nucleus transcriptomic data and demonstrating Piezo2 is indeed present in some V1^{Pou6f2} and V1^{Calb1} interneurons (Figure 4e). Finally, at the RNA level we used multiplexed RNAScope to validate several transcription factors and ion channels expressed in V1 interneurons (Figure 4b,d and the new Supplementary Figure 4). Additional evidence of the quality of our transcriptomic data can be seen in our snRNA-

seq experiments in En1 KO mice, which led to our identification of the V1^{Pou6f2_Nr5a2} subset affected by loss of *En1*, and independently confirmed by immunohistochemical analysis.

Taken together, our analysis and experimental validation collectively provide strong evidence for the validity of our transcriptomic dataset.

2. *Another major concern is that the tail suspension assay used to assess hyperflexion of the hindlimbs is not a locomotor assay, hence it cannot be easily compared to previously published work (Britz et al 2015, Allodi et al 2021). The authors claim absence of hyperflexion after En1 ablation, however changes in swing-stance phase during locomotion are not analysed. The involvement of sensory feedback (e.g., Ia afferents) in the tail suspension assay may also differ from the feedback involved in locomotion. Furthermore, it is unclear why early postnatal time points were chosen for behavioural analysis rather than adult ones, especially for the conditional KO mice. Hyperflexion linked to En1 silencing is well described in literature, so the authors need to provide further behavioural evidence. Moreover, from the Material and Methods description, it seems that the video frames included in the behavioural analysis were manually picked. This may lead to the introduction of biases in the analysis. The videos should be analysed in their entirety."*

This is a valid concern, also raised by another reviewer. The neonatal tail suspension assay was chosen because it is a simple, well-validated method previously used to assess limb movements in mice.[16] However, as the reviewer notes, it differs from normal locomotion in important ways, including that it is performed at a developmental time at which motor circuits are still maturing, and likely involves many differences in sensory input (both proprioceptive and cutaneous) compared to that received during locomotion. To address this, we performed kinematic analyses of locomotor behavior in adult V1 ablated and En1 cKO mice. V1 ablation results in hyperflexion of the ankle during the swing phase of the step cycle, exemplified by the reduced angle of the ankle and the increased height of the hindpaw that is a consequence of the hyperflexed state (see Figure 1f-h and Supplementary Figure 1d). In contrast, kinematic analysis of En1 cKO animals showed they do not exhibit hyperflexion, with limb position characterized instead by a slight hypoflexion (see Figure 1n-o and Supplementary Figure 1e). These data reinforce the central observation that the hyperflexion phenotype resulting from loss of V1 interneurons is not present upon deletion of *En1*, including under more normal physiological behaviors.

Regarding the selection of video frames for analysis in the tail suspension assay, it is important to emphasize that apart from clamping their tail, animals are unrestrained and free to move in all dimensions. From a practical perspective, this means that the animals occasionally twist their bodies and limbs away from the camera, making measurements of joint position in these frames highly inaccurate or impossible to measure. Accordingly, animals across conditions were only analyzed during periods in which they were facing forward towards the camera. All frames meeting these conditions (minimum of 2000 frames per animal per video) were included in our analysis.

3. *It is unclear why the authors do not use multiplexing analysis to validate that the clade-specific markers are mutually exclusive (Fig 3). Multiplexing analysis (detecting either proteins or mRNAs) is routinely used to validate transcriptomics data. The authors state that only 39 nuclei were found to co-express Pou6f2 and Sp8, however, the gene co-expression atlas*

seems to show differently (with 8568 nuclei co-expressing Foxp2 and Pou6f2). Again, this might be an issue related to the online tool, but since the main markers are only 5 (Foxp2, Pou6f2, Sp8, MafA and Rnf220) a multiplexing proteomics would be a more appropriate technique to use here.

This point is well-taken and was also raised by Reviewer 3. In previous work we examined the expression of four clade markers (Foxp2, MafA, Sp8, and Pou6f2) at the protein level using immunohistochemistry, revealing each was expressed in V1 interneurons in a mutually exclusive manner.[9] However, protein and transcript levels do not always correlate well,[17] raising the question of whether these markers at the transcript level really delineate different subsets of V1 interneurons.

Regarding the discrepancy between our statement that only 39 nuclei were found to co-express Pou6f2 and Sp8 versus the 8,568 nuclei co-expressing these in the online tool, we thank the reviewer for asking about this and apologize for this confusion. The discrepancy arose because the previous online tool referenced by the reviewer displayed raw read counts rather than log-normalized values. We did not consider the presence of a single read to be sufficient for assigning nuclei to a specific clade identity, given the concern that low levels of ambient RNA could cause misassignment. Rather, we assigned nuclei identity on a per-cluster level based on average z-scores of log-normalized expression of the clade markers in the clusters and then assigning each cluster to the clade with a positive z-score. This method has the additional advantage of accounting for high dropout rates characteristic of single-nucleus transcriptomic data. Given this confusion, we have removed the statements in the text reporting co-expression of the clade markers on an individual cell basis and removed the co-expression panel from the online tool.

Given these concerns, we have performed additional independent validation our transcriptomic data and prior immunohistochemical studies. As suggested by the reviewer, we have now included multiplexed RNAScope *in situ* hybridization data for detection of *Foxp2*, *Calb1* (a proxy for the MafA subset), *Sp8*, *Pou6f2*, and the newly reported marker *Rnf220*, gated to lineage-traced V1 interneurons (Supplementary Figure 4). Expression was assessed for four markers simultaneously (plus a fifth channel to detect tdTomato expression in the V1 lineage-traced neurons) in three iterations to cover all possible combinations. This was performed using the Multiplex kit, as we found this provided substantially better sensitivity than the HiPlex assay. Importantly, we found that all five markers are expressed in V1 interneurons in a mutually exclusive fashion. These experiments therefore confirm these five genes represent markers that indeed delineate different V1 subsets, in agreement with our analysis of protein expression, and also provide an additional measure of confidence in the conclusions from our transcriptomic dataset. We thank the reviewer for prompting us to clarify these issues.

References

1. Worthy AE*, et al.* Spinal V1 inhibitory interneuron clades differ in birthdate, projections to motoneurons, and heterogeneity. *Elife* **13**, (2024).

2. Benito-Gonzalez A, Alvarez FJ. Renshaw cells and Ia inhibitory interneurons are generated at different times from p1 progenitors and differentiate shortly after exiting the cell cycle. *J Neurosci* **32**, 1156-1170 (2012).

3. Chapman PD*, et al.* A brain-wide map of descending inputs onto spinal V1 interneurons. *Neuron*, (2025).

4. Gosgnach S*, et al.* V1 spinal neurons regulate the speed of vertebrate locomotor outputs. *Nature* **440**, 215-219 (2006).

5. Saueressig H, Burrill J, Goulding M. Engrailed-1 and netrin-1 regulate axon pathfinding by association interneurons that project to motor neurons. *Development* **126**, 4201-4212 (1999).

6. Sapir T*, et al.* Pax6 and engrailed 1 regulate two distinct aspects of renshaw cell development. *J Neurosci* **24**, 1255-1264 (2004).

7. Noga BR, Shefchyk SJ, Jamal J, Jordan LM. The role of Renshaw cells in locomotion: antagonism of their excitation from motor axon collaterals with intravenous mecamylamine. *Exp Brain Res* **66**, 99-105 (1987).

8. Enjin A*, et al.* Developmental Disruption of Recurrent Inhibitory Feedback Results in Compensatory Adaptation in the Renshaw Cell-Motor Neuron Circuit. *J Neurosci* **37**, 5634-5647 (2017).

9. Bikoff JB*, et al.* Spinal Inhibitory Interneuron Diversity Delineates Variant Motor Microcircuits. *Cell* **165**, 207-219 (2016).

10. Bakken TE*, et al.* Single-nucleus and single-cell transcriptomes compared in matched cortical cell types. *PLoS One* **13**, e0209648 (2018).

11. Russ DE*, et al.* A harmonized atlas of mouse spinal cord cell types and their spatial organization. *Nat Commun* **12**, 5722 (2021).

12. Grewer C, Gameiro A, Rauen T. SLC1 glutamate transporters. *Pflugers Arch* **466**, 3-24 (2014).

13. Bjorn-Yoshimoto WE, Underhill SM. The importance of the excitatory amino acid transporter 3 (EAAT3). *Neurochem Int* **98**, 4-18 (2016).

14. Su ZZ*, et al.* Insights into glutamate transport regulation in human astrocytes: cloning of the promoter for excitatory amino acid transporter 2 (EAAT2). *Proc Natl Acad Sci U S A* **100**, 1955-1960 (2003).

15. Delile J, Rayon T, Melchionda M, Edwards A, Briscoe J, Sagner A. Single cell transcriptomics reveals spatial and temporal dynamics of gene expression in the developing mouse spinal cord. *Development* **146**, (2019).

16. Britz O*, et al.* A genetically defined asymmetry underlies the inhibitory control of flexor-extensor locomotor movements. *Elife* **4**, (2015).

17. Mair F*, et al.* A Targeted Multi-omic Analysis Approach Measures Protein Expression and Low-Abundance Transcripts on the Single-Cell Level. *Cell Rep* **31**, 107499 (2020).

Response to remaining Reviewer comments

Reviewer #4 (Remarks on code availability):

The code is now available, however the README files are missing - this should be added before publication.

Our response: README files for all code have been added to Github.

Gene Expression

Gene 1:

Slc1a1

Gene 2:

Slc6a5

Toggle plot controls

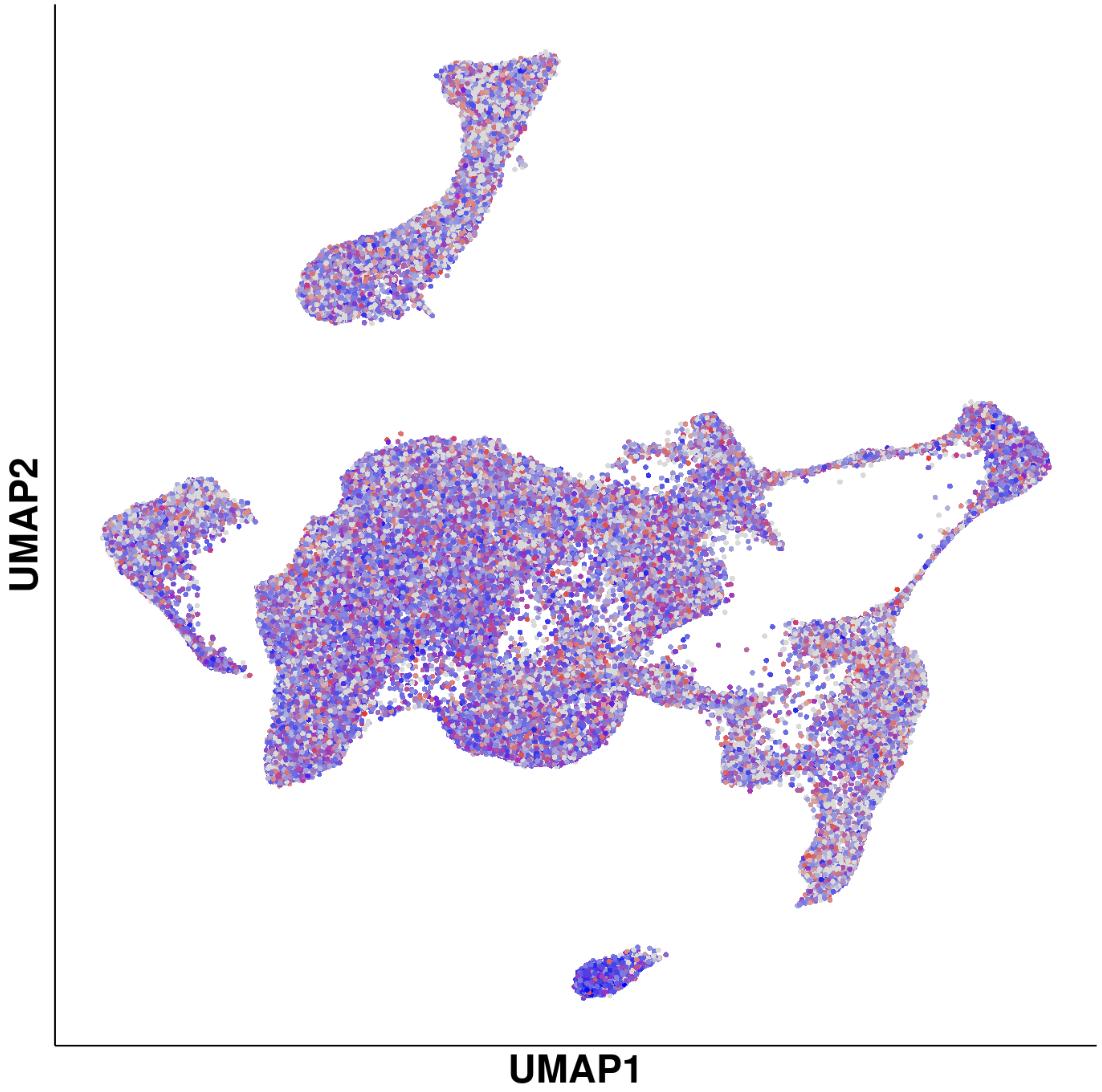

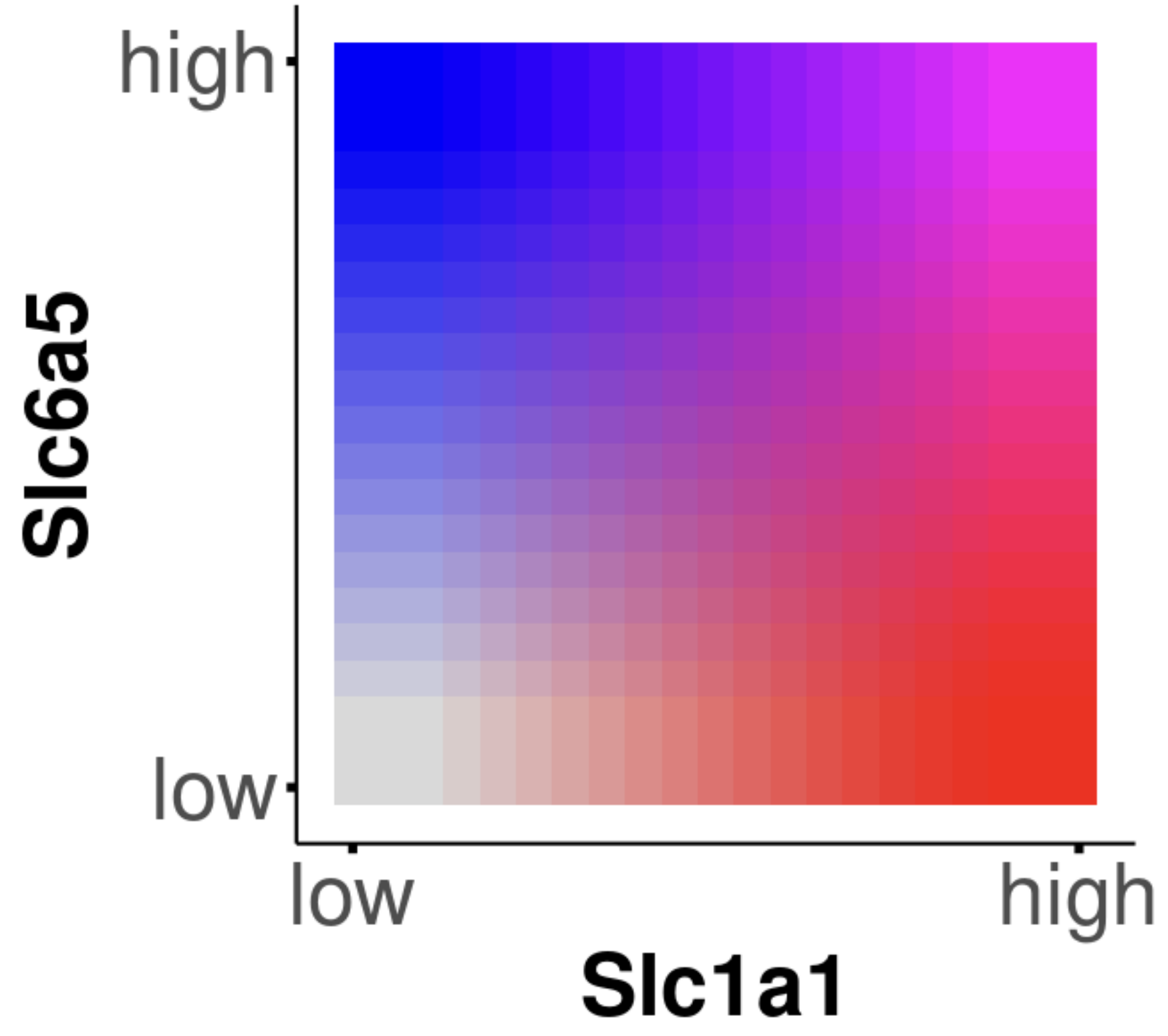

Download PDF Download PNG

Cell numbers

expression > 0	nCells	percent
both	27760	31.02
Slc1a1	8674	9.69
Slc6a5	36019	40.25
none	17041	19.04

Copy CSV Excel

Gene Expression

Gene 1:

Slc1a1

Gene 2:

Slc1a2

Toggle plot controls

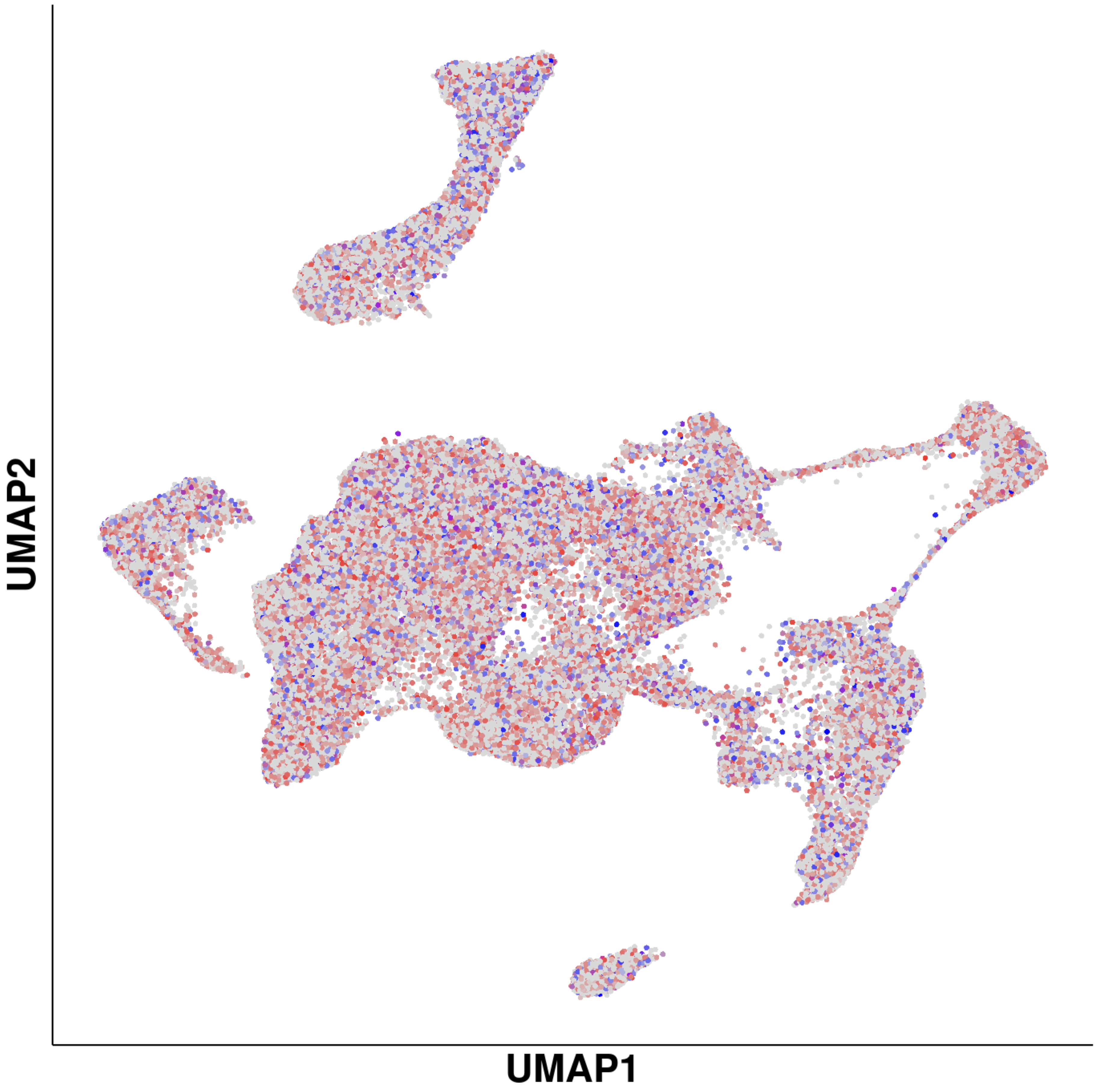

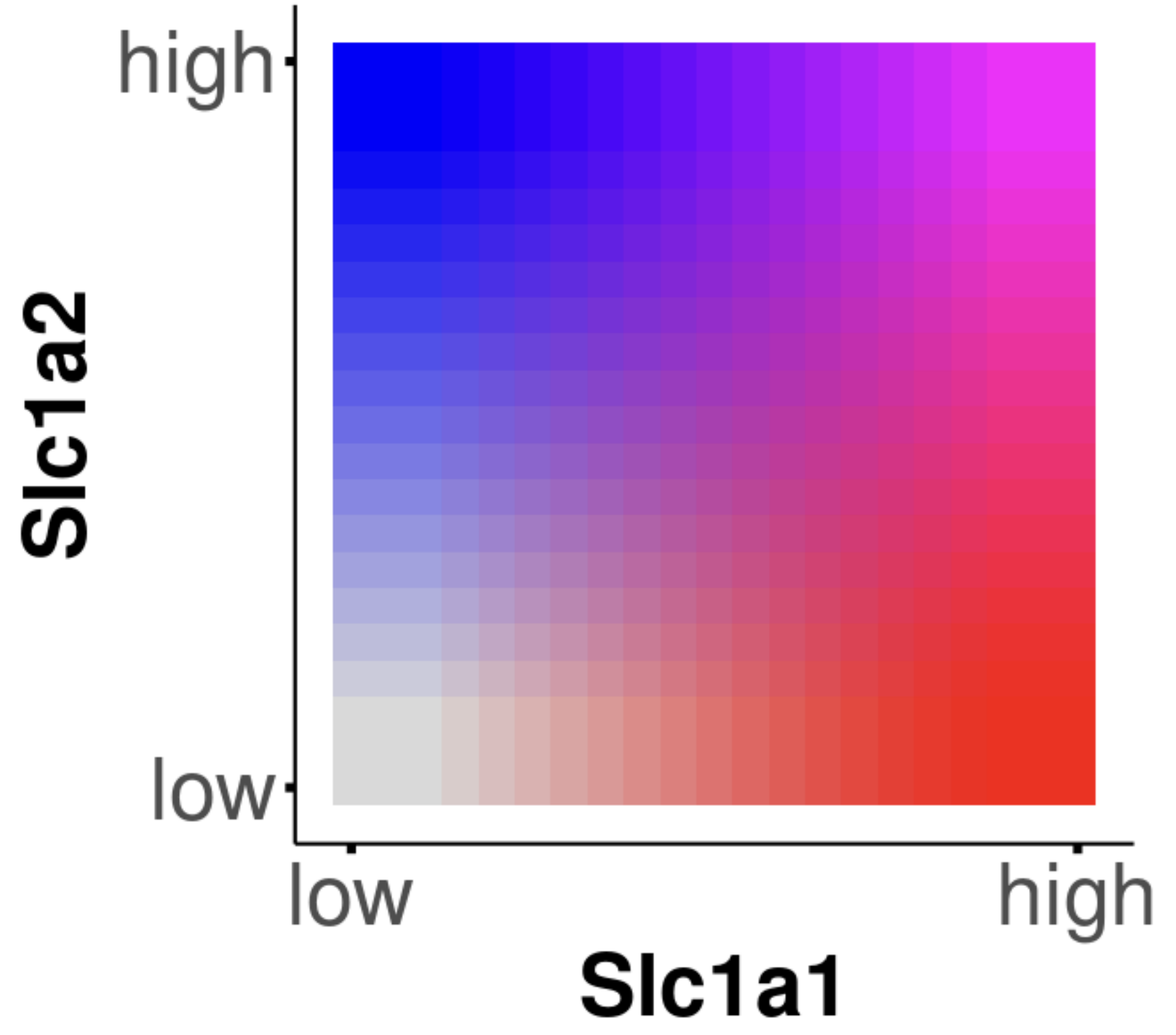

Download PDF

Download PNG

Cell numbers

expression > 0	nCells	percent
both	5950	6.65
Slc1a1	30484	34.06
Slc1a2	9455	10.56
none	43605	48.72

Copy CSV Excel
