# Supplementary material for: Transcriptomic analysis of spinal V1 interneurons informs their multifunctional role in motor output
Source: Nat Commun. 2026 Aug 10;17:9614. doi: 10.1038/s41467-026-76522-3 (PMC13554063; doi:10.1038/s41467-026-76522-3)
Supplement: Supplementary file 3 [file 41467_2026_76522_MOESM1_ESM.pdf]

Supplementary Information

Transcriptomic analysis of spinal V1 interneurons informs their multifunctional role in motor output

Alexandra J. Trevisan[1], Katie Han[1], Phillip D. Chapman[1], Anand S. Kulkarni[1], Jennifer M. Hinton[1], Ines Klein[2], Cody Ramirez[1], Alfonso Lavado[3], Graziana Gatto[2], Mariano I. Gabitto[4,5], Vilas Menon[6], and Jay B. Bikoff[1,*]

[1]Department of Developmental Neurobiology, St. Jude Children's Research Hospital, Memphis, TN, 38105, USA
[2]Department of Neurology, University Hospital of Cologne, Cologne, 50937, Germany
[3]Center for Pediatric Neurological Disease Research, St. Jude Children's Research Hospital, Memphis, TN 38105, USA
[4]Allen Institute for Brain Science, Seattle, WA, 98109, USA
[5]Department of Statistics, University of Washington, Seattle, WA, 98109, USA
[6]Department of Neurology, Center for Translational and Computational Neuroimmunology, Columbia University, New York, NY, 10033, USA
*Correspondence: jay.bikoff@stjude.org

This PDF file includes:

Supplementary Figures 1-7
Supplementary Tables 1-2

Supplementary Fig. 1

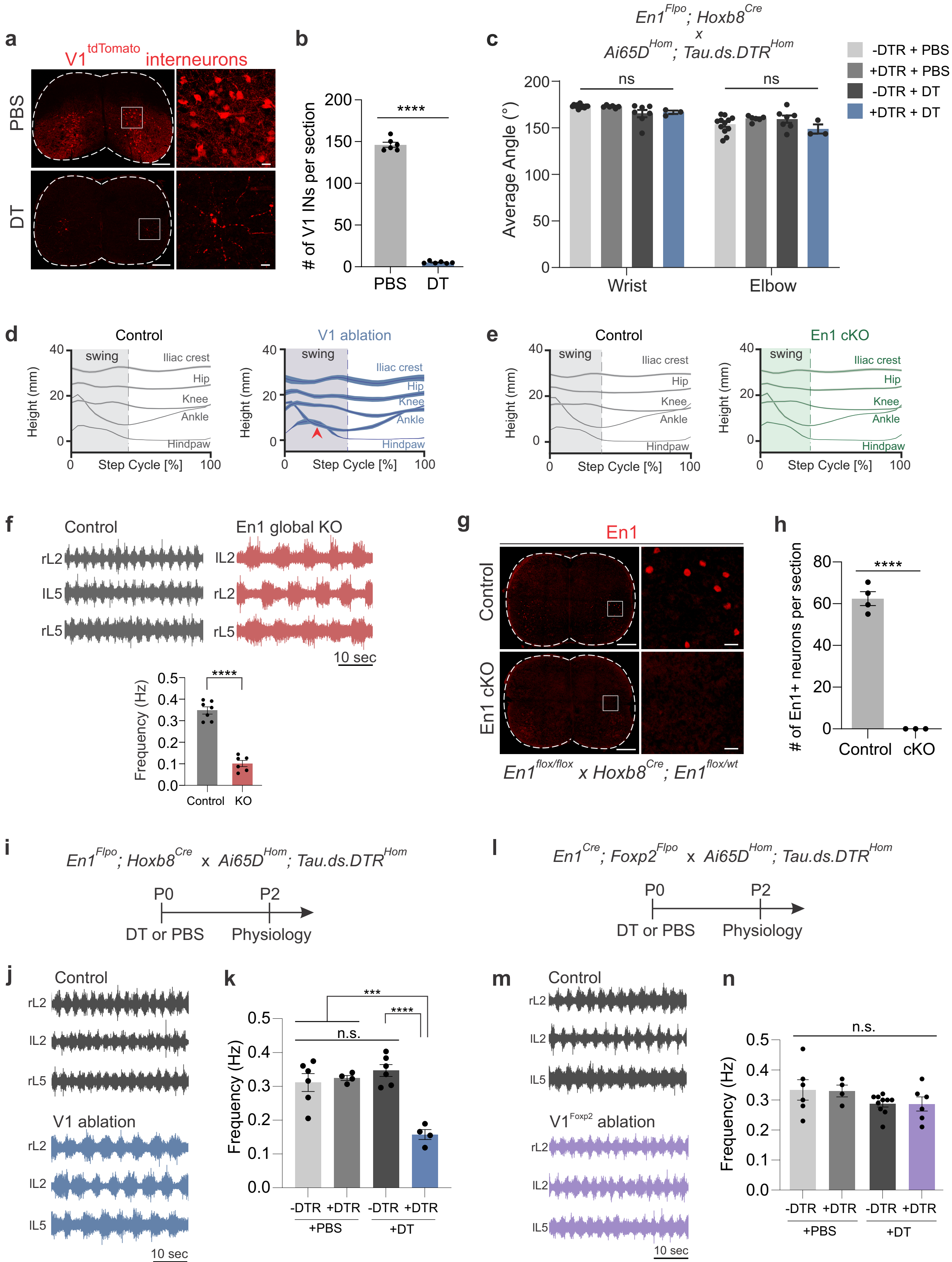

Supplementary Fig 1. Validation of V1 ablation and *En1* conditional knockout experiments, and analysis of rhythmic locomotor activity in V1^{Foxp2} ablated mice.

(a,b) Immunohistochemical analysis (a) and quantification (b) of V1 interneurons in P7 lumbar spinal cords of control (+PBS) or V1 ablated (+DT) quadruple-heterozygous (*En1^{Flpo}; Hoxb8^{Cre}; Ai65D; Tau.ds.DTR*) mice demonstrates loss of V1 interneurons 7 days post-DT administration (n = 6 mice, mean ± SEM, ****p < 0.0001, unpaired two-tailed t-test). Scale bars = 200 μm or 20 μm (inset). **(c)** The average angles of the wrist and elbow joints during tail suspension were not significantly changed upon ablation of V1 interneurons (n = 12, 6, 7, and 3 mice, respectively; mean ± SEM, two-way ANOVA followed by Tukey HSD test, ns = not significant). **(d)** Line graphs showing changes in height of hindlimb landmarks during a normalized step cycle in control (gray, n = 21) and V1 ablated (blue, n = 4) mice. The swing phase of the step cycle is shaded in gray and the stances phase in white. Red arrowhead indicates the peak of hyperflexion mid-swing. SEMs are represented as shaded areas. **(e)** Line graphs showing changes in height of hindlimb landmarks during a normalized step cycle in control (gray, n = 22) and *En1 cKO* (green, n = 13) mice. SEMs are represented as shaded areas. **(f)** Top, ventral root recordings during fictive locomotion in control (*En1^{Cre}* heterozygous) or global *En1* KO (*En1^{Cre}* homozygous) P0 spinal cords. Bottom, reduced frequency of fictive locomotor activity in global *En1* KO mice (n = 6 to 7 mice, mean ± SEM, ****p < 0.001, unpaired two-tailed t-test). **(g,h)** Immunohistochemical analysis (g) and quantification (h) showing complete loss of En1 expression in *En1 cKO* (*Hoxb8^{Cre}; En1$^{flox/flox}$*) mice in P0 lumbar spinal cord (n = 3 to 4 mice, ****p < 0.0001, unpaired two-tailed t-test). Scale bars = 200 μm or 20 μm (inset). **(i)** Schematic of the V1 ablation paradigm, with physiology performed 2 days post-DT administration rather than 4 days post-DT to match the time course of the V1^{Foxp2} ablation experiments. **(j)** Ventral root recordings from control (-DTR + DT, gray) or V1 ablated (+DTR +DT, blue) mice demonstrating slowed locomotor-like rhythm only 2 days after DT administration. **(k)** Reduced frequency of fictive locomotor activity upon DT-mediated ablation of V1 interneurons (n = 6, 4, 6, and 4 mice, respectively; mean ± SEM, ***p < 0.001, ****p < 0.0001, two-way ANOVA followed by Holm-Sidak test for multiple comparisons). **(l)** Schematic of the V1^{Foxp2} ablation paradigm, with physiology performed 2 days post-DT administration due to lethality at older ages. **(m)** Ventral root recordings during fictive locomotion in control (-DTR + DT, gray) or V1^{Foxp2}-ablated (+DTR + DT, purple) mice. **(n)** Normal rhythmic locomotor-like activity is observed in V1^{Foxp2}-ablated mice (n = 6, 4, 10, and 6 mice, respectively; mean ± SEM, n.s. = not significant by two-way ANOVA). See Supplementary Table 2 for statistics of all comparisons. Source data are provided as a Source Data file.

Supplementary Fig. 2

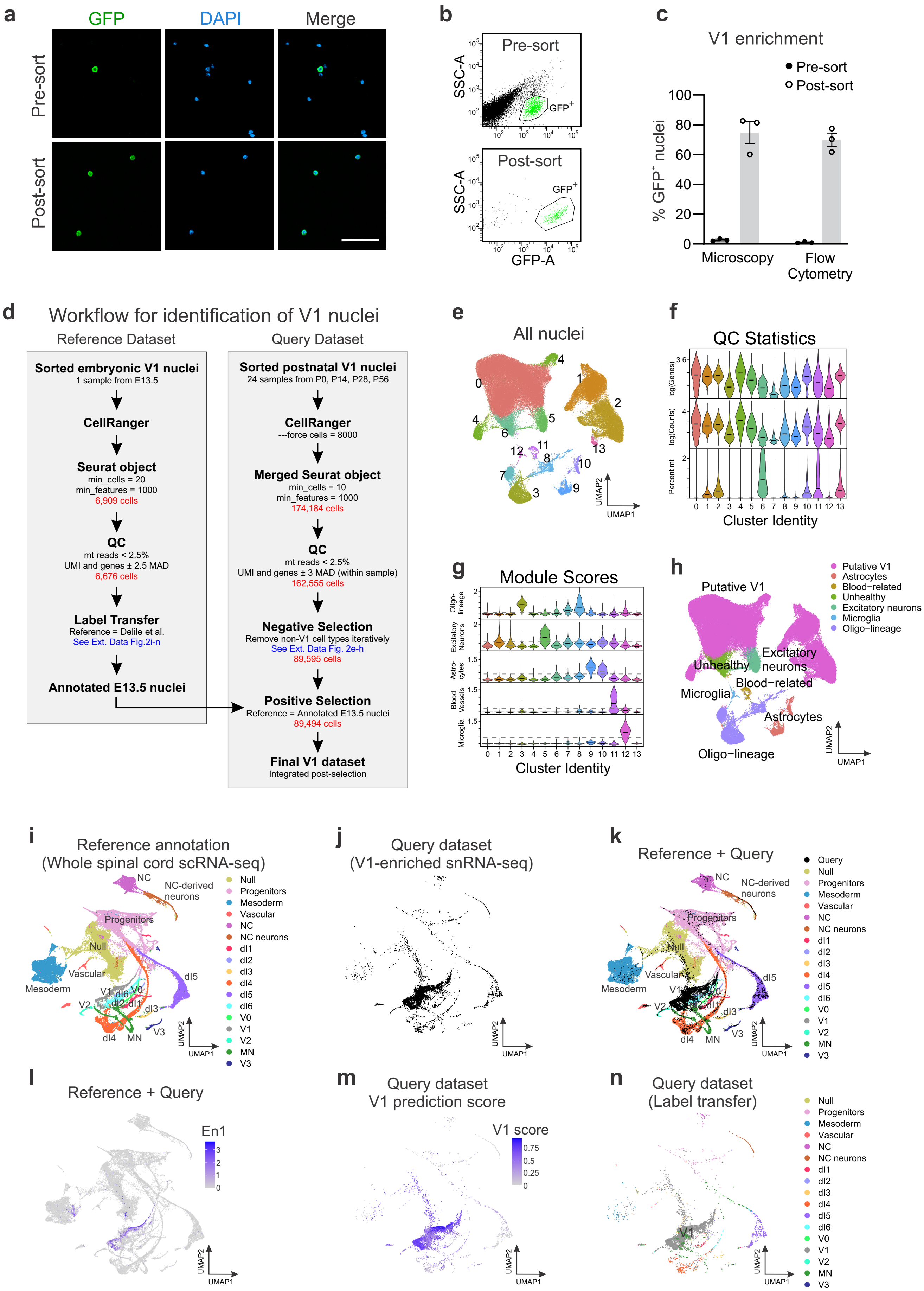

Supplementary Fig. 2. Enrichment of V1 nuclei via fluorescence activated nuclei sorting, and subsequent identification of V1 nuclei.

(a-c) Enrichment of V1 nuclei by fluorescence activated nuclei sorting (FANS). **(a)** Example images of nuclei isolated from P0 *En1^{Cre}; RC::lsl.Sun1-sfGFP* (*En1^{INTACT}*) spinal cords before (top) and after (bottom) FANS. Scale bar = 50 μm. **(b)** Representative FANS analysis of spinal cord nuclei before sorting (top) and then re-analyzed after sorting (bottom). **(c)** Quantification of V1 nuclei enrichment assessed via microscopy or flow cytometry before sorting (2.7% ± 0.5% and 1.1% ± 0.3%, respectively) and after sorting (74.7% ± 7.3% and 69.9% ± 4.6%, respectively) (n = 3 independent trials, mean ± SEM).

(d) Workflow outlining the identification of V1 nuclei within the postnatal nuclei dataset (Query Dataset) by using both negative and positive selection criteria.

(e-h) Negative selection to remove contaminating (non-V1 interneuron) nuclei. **(e)** UMAP showing clustering of all nuclei meeting quality control (QC) cutoffs across all ages, replicates, and anatomical regions combined. **(f)** The number of genes detected per nucleus, total reads per nucleus, and percent of mitochondrial reads are shown per cluster. Cluster 6 was identified as unhealthy due to the low number of genes and counts, and high number of mitochondrial reads. **(g)** Gene module scores per cluster for non-V1 cell types. Clusters above the threshold (dotted line) were identified as the corresponding cell type. See Supplementary Data 2 for genes used to create the module score. **(h)** The UMAP shown in (e) with clusters assigned an identity per the selection measures described in (f) and (g). Clusters 0, 1, 2, 4, and 13 were used for subsequent rounds of negative selection followed by positive selection of V1 interneurons.

(i-n) Positive identification of V1 interneurons using label transfer. **(i)** E9.5-E13.5 whole spinal cord scRNA-seq data[29] was used as a reference data set. **(j)** E13.5 *En1::Cre^{INTACT}* snRNA-seq query data (black) enriched for V1 interneurons by flow cytometry. **(k)** Data set from (j) projected onto reference data (i). **(l)** Expression of the transcription factor *En1*. **(m)** V1 label transfer prediction scores. **(n)** Final assignments for the E13.5 snRNA-seq data set, used for subsequent positive selection of our postnatal data. Source data are provided as a Source Data file.

Supplementary Fig. 3

a

Region / Age	P0	P14	P28	P56	Total
Cervical	*9800*	*9098*	*6122*	*8745*	*33765*
Thoracic	*9030*	*5672*	*5867*	*9004*	*29573*
Lumbar	*10071*	*5620*	*5653*	*4812*	*26156*
Total	*28901*	*20390*	*17642*	*22561*	*89494*

b

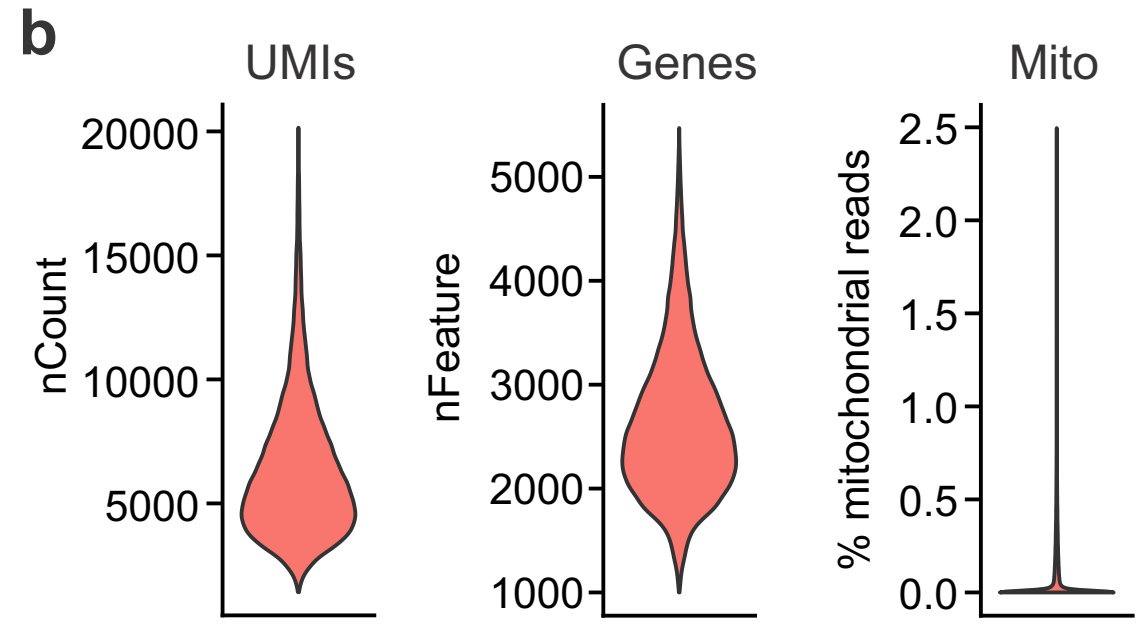

c **d** **e**

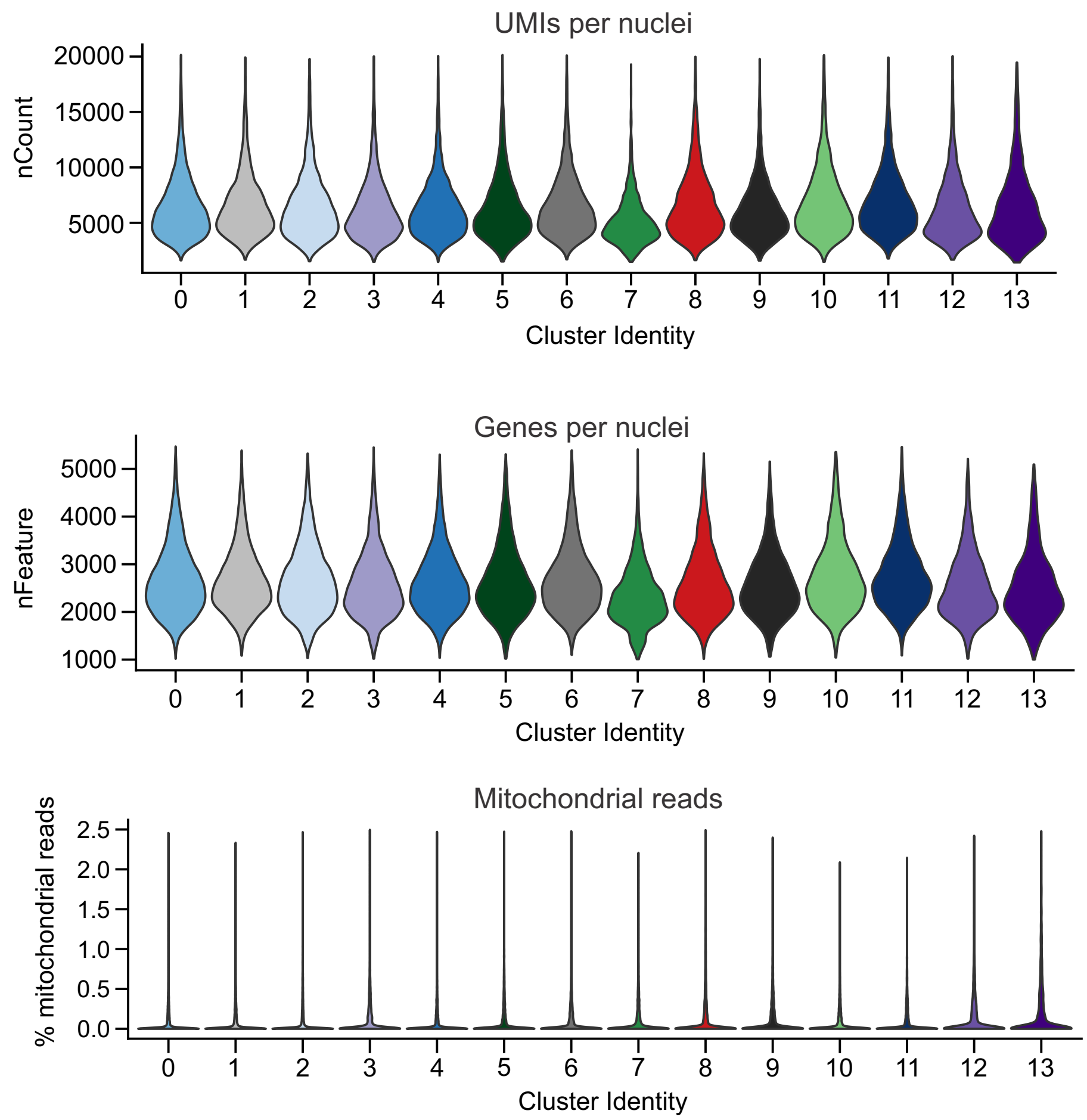

f

Cluster	Cells	%
0	*20425*	*22.9*
1	*7404*	*8.3*
2	*6894*	*7.7*
3	*6832*	*7.6*
4	*6670*	*7.5*
5	*6604*	*7.4*
6	*6359*	*7.1*
7	*6224*	*6.9*
8	*5143*	*5.7*
9	*4411*	*4.9*
10	*4271*	*4.8*
11	*3897*	*4.4*
12	*2400*	*2.7*
13	*1960*	*2.2*

Supplementary Fig. 3. Quality Control (QC) statistics on V1 interneuron nuclei.
(a) Table showing the total number of nuclei identified as V1 interneurons grouped by age and anatomical location. Colors represent low (blue) to high (red) proportions across all categories. **(b)** Overall QC statistics on V1 interneurons showing total number of unique molecular identifier (UMI)-corrected counts per nucleus, number of genes detected per nucleus, and the percentage of mitochondrial reads per nucleus. **(c-e)** Similar to (b) except the same statistics were parsed by the cluster identification shown in Fig. 2c. **(f)** Table displaying the number and percentage of nuclei in each cluster.

Supplementary Fig. 4

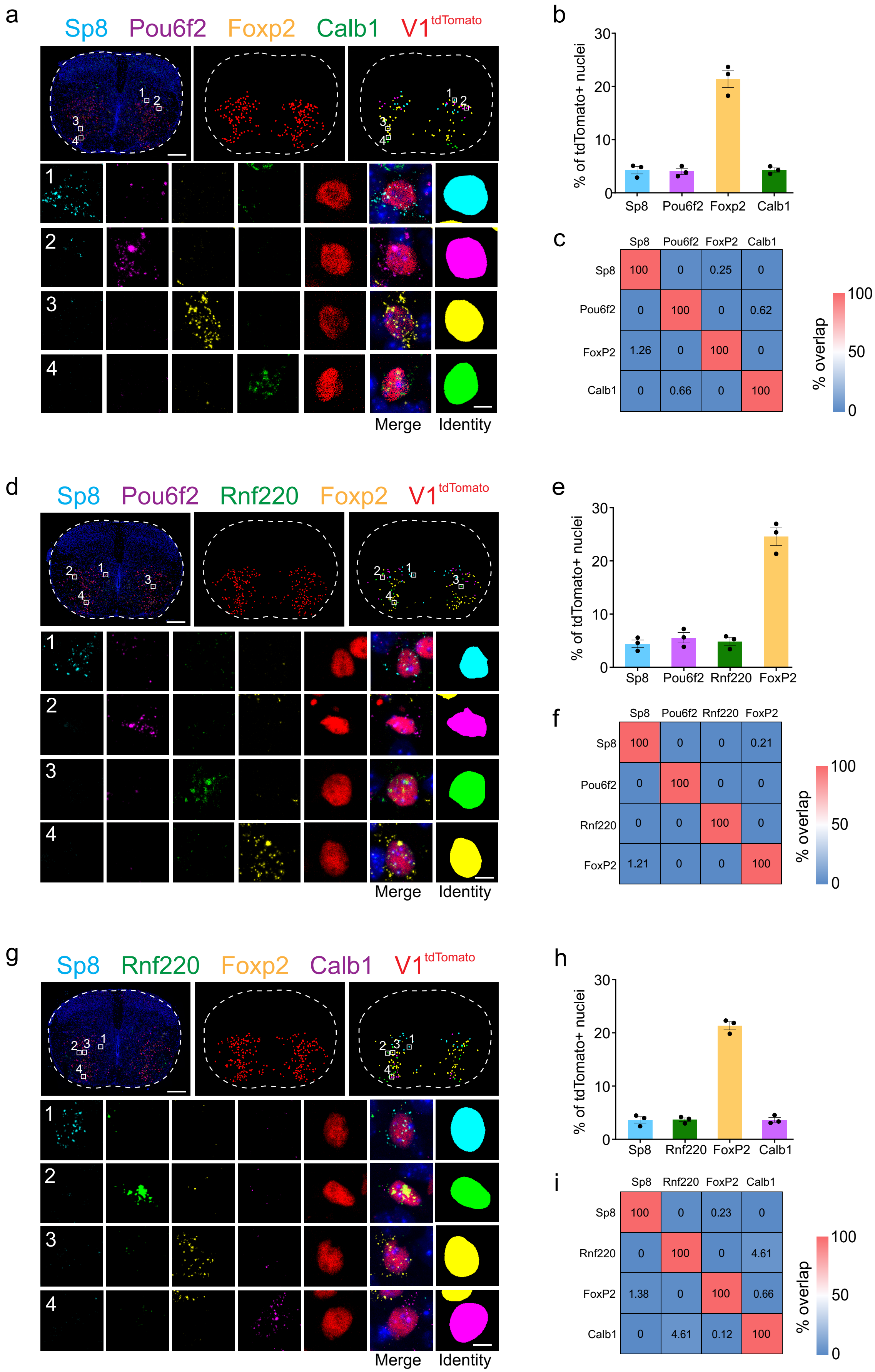

Supplementary Fig. 4. Validation of key marker gene expression in V1 interneurons via multiplexed RNAScope.

(a-c) Multiplexed RNAScope fluorescent *in situ* hybridization for *Sp8* (blue), *Pou6f2* (purple), *Foxp2* (yellow), and *Calb1* (green) transcripts in P0 lumbar spinal cord of *En1^{Cre}; Ai75* mice. V1 interneurons were identified via immunohistochemical detection of nuclear tdTomato (red). **(a)** Representative examples of RNAScope fluorescent mRNA detection showing expression of each marker within V1 interneurons. Identity assigned to V1 nuclei is shown in the bottom rightmost column. Scale bars = 200 μm (top) or 10 μm (inset). **(b)** Percentage of tdTomato^{+} V1 nuclei positive for each marker (mean ± SEM; n = 3 mice). **(c)** Matrix of pairwise overlap for each marker within V1 interneurons, demonstrating they are expressed in a mutually exclusive manner.

(d-f) Multiplexed RNAScope fluorescent *in situ* hybridization and quantification for *Sp8* (blue), *Pou6f2* (purple), *Rnf220* (green), and *Foxp2* (yellow) transcripts in P0 lumbar spinal cord of *En1^{Cre}; Ai75* mice, with immunohistochemical detection of nuclear tdTomato to identify V1 interneurons, as in (a-c).

(g-i) Multiplexed RNAScope fluorescent *in situ* hybridization and quantification for *Sp8* (blue), *Rnf220* (green), and *Foxp2* (yellow), and *Calb1* (purple) transcripts in P0 lumbar spinal cord of *En1^{Cre}; Ai75* mice, with immunohistochemical detection of nuclear tdTomato to identify V1 interneurons, as in (a-c). Source data are provided as a Source Data file.

Supplementary Fig. 5

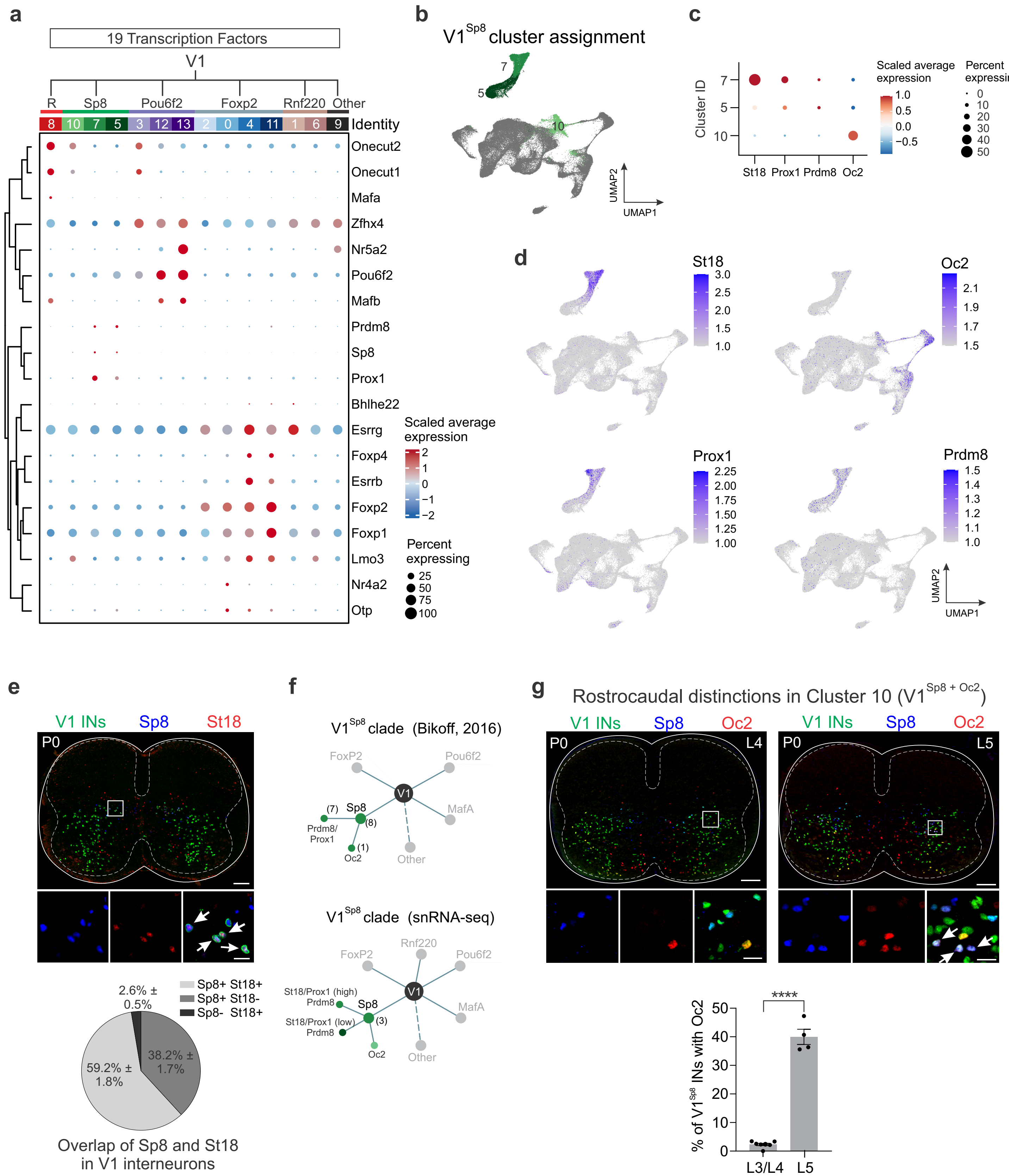

Supplementary Fig. 5. Analysis of transcription factor expression and V1^{Sp8} clade structure. **(a)** Dot plot showing scaled average expression of the 19 transcription factors (TFs) described previously in Bikoff et al.,[18] all of which were validated via immunohistochemistry (IHC). Note that several genes were detected at low levels but still showed biased expression. **(b)** V1^{Sp8} cluster assignment, as described in Fig. 2f. **(c)** Corresponding dot plot highlighting St18, Prox1, Prdm8, and Oc2 expression within the V1^{Sp8} clade. **(d)** UMAP plots of St18, Prox1, Prdm8, and Oc2 showing expression in V1 interneurons. **(e)** Top, IHC validation of St18 expression in V1 interneurons (arrows) in P0 lumbar spinal cord of *En1^{INTACT}* mice. Scale bars = 100 μm (top) or 20 μm (inset). Bottom, the proportion of Sp8 and St18 co-expression in V1 interneurons as assessed by IHC. **(f)** Top, simplified V1^{Sp8} clade diagram adapted from Bikoff, et al.[18] showing delineation of V1^{Sp8} neurons into seven Prdm8/Prox1 subsets and a single Oc2-expressing subset. Bottom, revised clade diagram based on snRNA-seq data, showing general alignment with previous analyses. **(g)** Top, IHC analysis of cluster #10 in P0 lumbar spinal cord of *En1^{Cre}; Tau.lsl.nLacZ* mice. Scale bars = 100 μm (top) or 20 μm (inset). Bottom, V1$^{Sp8+Oc2}$ interneurons (arrows) were significantly enriched in caudal (L5) spinal segments compared to more rostral (L3/L4) segments, thus highlighting segmental differences in V1 cell types. ****p < 0.0001, unpaired *t*-test. See Supplementary Table 2 for statistics of all comparisons. Source data are provided as a Source Data file.

Supplementary Fig. 6

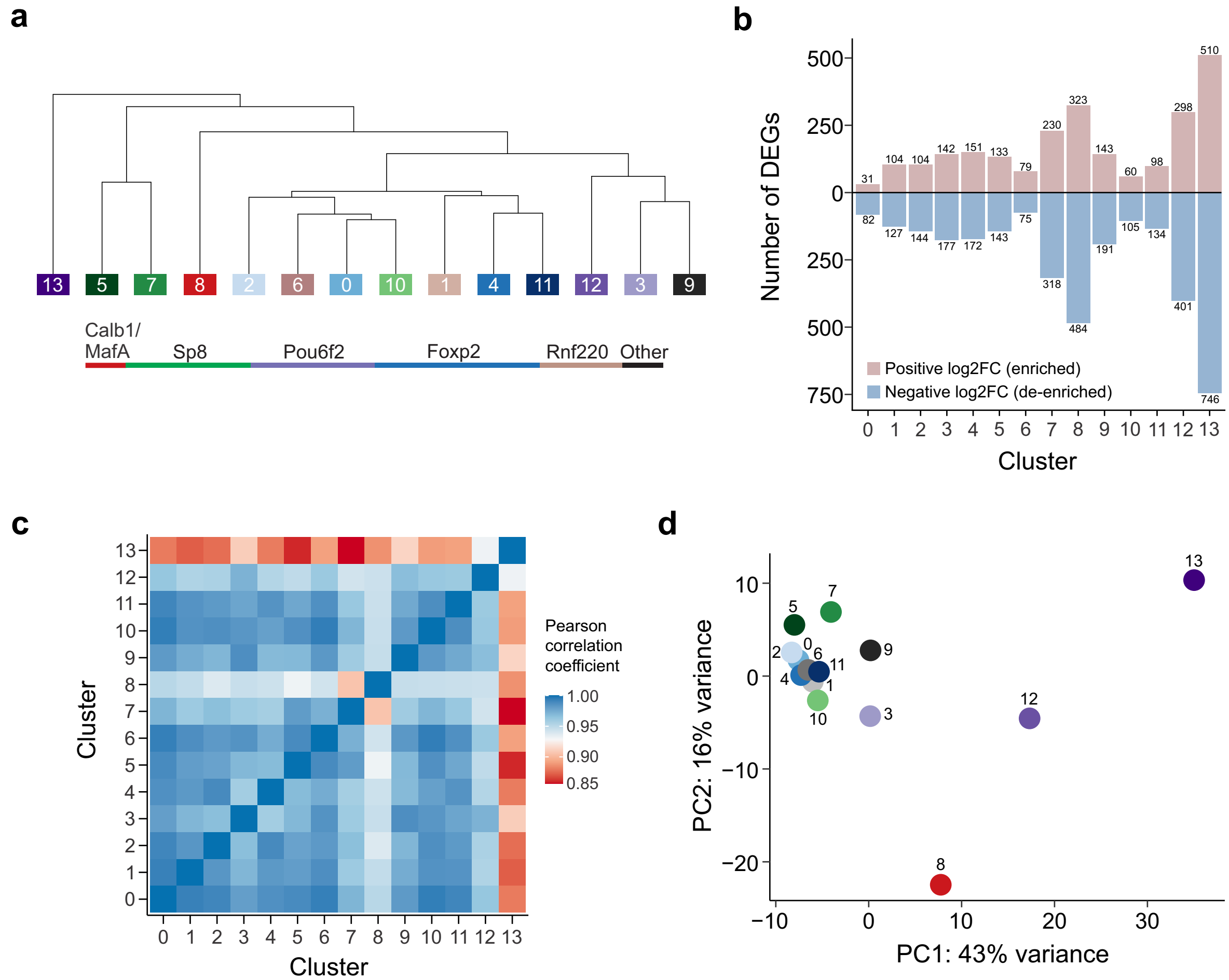

Supplementary Fig. 6. Taxonomic relationship and similarity of V1 clusters.
(a) Dendrogram showing phylogenetic analysis among the 14 clusters described in Fig. 2c, based on the top 3000 most variable genes. (**b)** Number of differentially expressed genes (DEGs) per cluster based on non-parametric Wilcoxon rank sum test for differential gene expression. **(c)** Heatmap of Pearson correlation coefficients based on the log-normalized average expression of all genes per cluster identified cluster #13 as unique. **(d)** Principal component analysis (PCA) plot of aggregated counts per cluster showing that cluster #13 drives most of the variance along PC1.

Supplementary Fig. 7

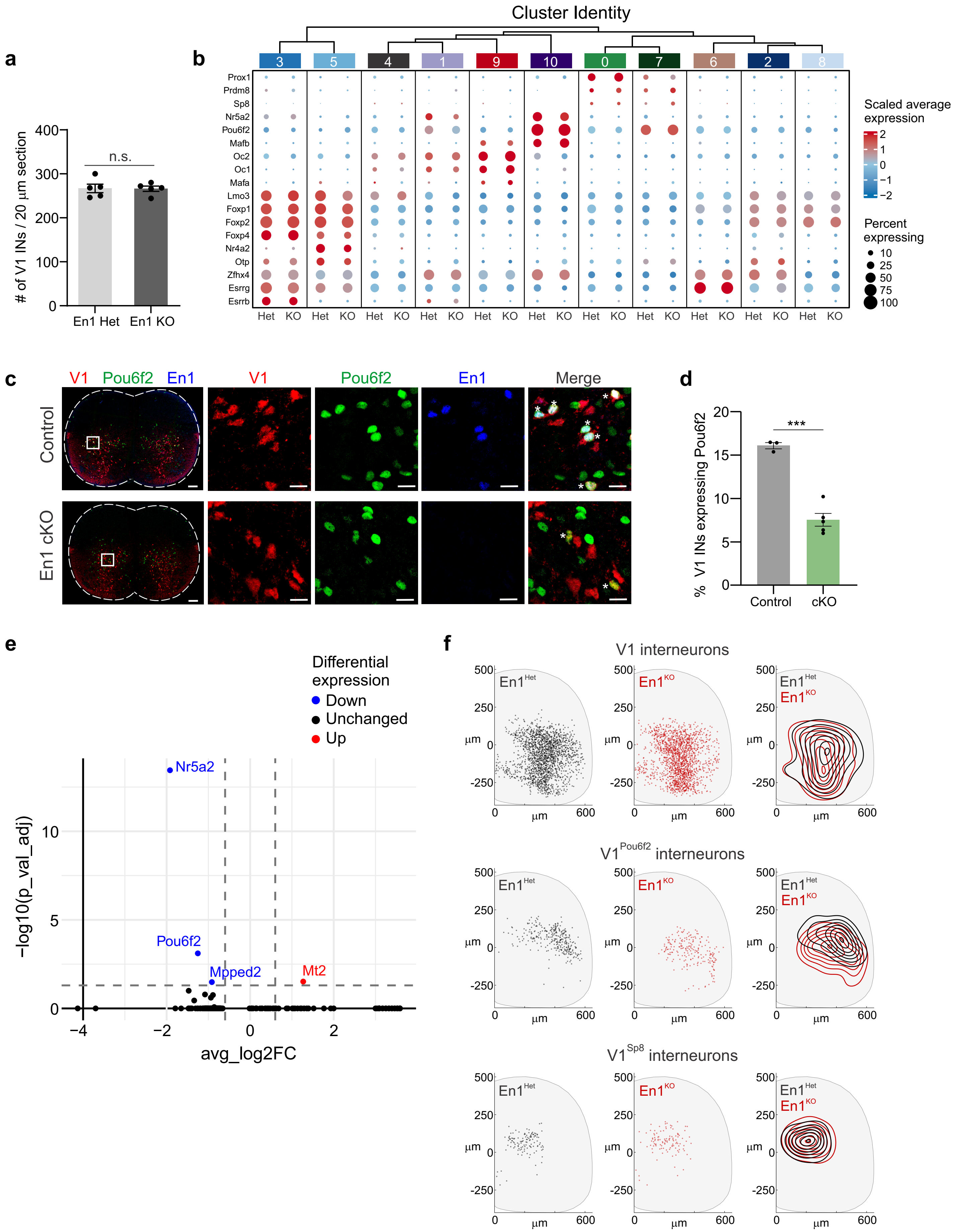

Supplementary Fig 7. Additional characterization of V1 interneuron number, gene expression, and spatial distribution in *En1^{Het}* and *En1^{KO}* mice.

(a) The total number of V1 interneurons in P0 lumbar spinal cords is similar in *En1^{Het}* and *En1^{KO}* mice (n.s. = not significant, p = 0.8154, unpaired two-tailed t-test). **(b)** Dot plot showing similar gene expression of 19 transcription factors in V1 nuclei from *En1^{Het}* and *En1^{KO}* mice. Note that while greatly reduced in number, the remaining *En1^{KO}* V1 nuclei in cluster #10 retained a normal *Pou6f2*$^{+}$ and *Nr5a2*$^{+}$ identity. **(c-d)** Reduction in the number of V1^{Pou6f2} interneurons in *En1 cKO* mice. **(c)** Top, representative image of P0 lumbar spinal cord from control (*En1$^{Flpo/flox}$; Ai65F*) mice, with V1 lineage-traced neurons (red) immunostained for Pou6f2 (green) and En1 (blue). Bottom, representative image of P0 lumbar spinal cord from *En1 cKO* (*Hoxb8^{Cre}*; *En1$^{Flpo/flox}$; Ai65F*) mice lacking En1 expression. V1^{Pou6f2} interneurons are indicated by asterisks. Scale bars = 100 μm or 20 μm (inset). **(d)** Quantification of V1^{Pou6f2} interneurons showing a reduction in *En1 cKO* mice (***p = 0.0002; unpaired two-tailed t-test). **(e)** Pseudo-bulk analysis of differentially expressed genes revealed that *Nr5a2* and *Pou6f2* are significantly downregulated in *En1^{KO}* V1 nuclei, compared with that in *En1^{Het}* nuclei. **(f)** Spatial distributions of the overall V1 interneuron population (top), V1^{Pou6f2} interneurons (middle), and V1^{Sp8} interneurons (bottom) in P0 lumbar spinal segments of *En1^{Het}* (black) and *En1^{KO}* (red) mice. The position of V1^{Pou6f2} neurons was altered in *En1^{KO}* mice, whereas the position of V1^{Sp8} interneurons was unaffected (2-dimensional Kolmogorov-Smirnov test: $p = 6.5 \times 10^{-13}$ for V1^{Pou6f2} neurons; p = 0.095 for V1^{Sp8} neurons). Distributions were based on the following sample size: *En1^{Het}* (V1): n = 4 animals, 13 hemisections, 1776 neurons from L3-L5 segments; *En1^{KO}* (V1): n = 5 animals, 13 hemisections, 1702 neurons from L3-L5 segments; *En1^{Het}* (V1^{Pou6f2}): n = 8 animals, 21 hemisections, 309 neurons from L3 segments; *En1^{KO}* (V1^{Pou6f2}): n = 5 animals, 21 hemisections, 213 neurons from L3 segments; *En1^{Het}* (V1^{Sp8}): n = 3 animals, 8 hemisections, 122 neurons from L3-L5 segments; and *En1^{KO}* (V1^{Sp8}): n = 3 animals, 6 hemisections, 113 neurons from L3-L5 segments. See Supplementary Table 2 for statistics of all comparisons. Source data are provided as a Source Data file.

Supplementary Table 1

Figures 2-4 Cluster ID#	Molecular Designation
0	$V1^{\text{Foxp2\_Nr4a2}}$
1	$V1^{\text{Rnf220\_Maf}}$
2	$V1^{\text{Foxp2\_Trpc4(high)}}$
3	$V1^{\text{Pou6f2\_Grm8(high)}}$
4	$V1^{\text{Foxp2\_Foxp4\_Hhip}}$
5	$V1^{\text{Sp8\_St18/Prox1(low)}}$
6	$V1^{\text{Rnf220\_Bcl11a}}$
7	$V1^{\text{Sp8\_St18}}$
8	$V1^{\text{Renshaw}}$
9	$V1^{\text{Nr5a2 (Pou6f2-negative)}}$
10	$V1^{\text{Sp8\_Oc2}}$
11	$V1^{\text{Foxp2\_Foxp4\_Ebf3}}$
12	$V1^{\text{Pou6f2\_Pebp4}}$
13	$V1^{\text{Pou6f2\_Nr5a2}}$

Figure 6 Cluster ID#	Molecular Designation
0	$V1^{\text{Sp8\_St18}}$
1	$V1^{\text{Pou6f2\_Grm8(high)}}$
2	$V1^{\text{Foxp2\_Foxp4\_Ebf3}}$
3	$V1^{\text{Foxp2\_Foxp4\_Hhip}}$
4	$V1^{\text{Nr5a2 (Pou6f2-negative)}}$
5	$V1^{\text{Foxp2\_Nr4a2}}$
6	$V1^{\text{Rnf220}}$
7	$V1^{\text{Sp8\_St18/Prox1(low)}}$
8	$V1^{\text{Foxp2\_Trpc4(high)}}$
9	$V1^{\text{Renshaw}}$
10	$V1^{\text{Pou6f2\_Nr5a2}}$

Supplementary Table 1. Cluster ID# and corresponding molecular nomenclature for clusters in Figures 2-4 (left) and Figure 6 (right).

Supplementary Table 2. Statistics summary

Summary of all statistics by figure panel.

Figure	Sample Size	Statistical test	Results
Figure 1C	Control group 1 in lightest gray bar (-DTR +PBS) n = 12<br>Control group 2 in medium gray bar (+DTR +PBS) n = 8<br>Control group 3 in darkest gray bar (-DTR + DT) n = 14<br>Experimental group 4 in blue (+DTR +DT) n = 6<br>Each n is an individual mouse (biological replicates)	2 way ANOVA followed by Holm Sidak multiple comparisons	**ANOVA**<br>Treatment (PBS/DT): F = 31.14. DFn = 1, DFd = 36, P < 0.0001<br>Genotype (-DTR/+DTR): F = 31.35. DFn = 1, DFd = 36, P < 0.0001<br>Interaction: F = 42.40. DFn = 1, DFd = 36, P < 0.0001<br>**Multiple comparisons**<br>**2 vs 1:** Mean 1 = 0.4139, Mean 2 = 0.4368, Mean diff = -0.02287, SE = 0.03355, t = 0.6817, DF = 36.00, Adj P value = 0.8722<br>**3 vs 1:** Mean 1 = 0.4363, Mean 2 = 0.4368, Mean diff = -0.0004762, SE = 0.02891, t = 0.01647 DF = 36.00, Adj P value = 0.9870<br>**4 vs 1:** Mean 1 = 0.1394, Mean 2 = 0.4368, Mean diff = -0.2974, SE = 0.03675, t = 8.092, DF = 36.00, Adj P value < 0.0001<br>**3 vs 2:** Mean 1 = 0.4363, Mean 2 = 0.4139, Mean diff = 0.02239, SE = 0.03257, t = 0.6874, DF = 36.00, Adj P value = 0.8722<br>**4 vs 2:** Mean 1 = 0.1394, Mean 2 = 0.4139, Mean diff = -0.2745, SE = 0.03969, t = 6.916, DF = 36.00, Adj P value < 0.0001<br>**4 vs 3:** Mean 1 = 0.1394, Mean 2 = 0.4363, Mean diff = -0.2969, SE = 0.03586, t = 8.279, DF = 36.00, Adj P value < 0.0001
Figure 1E, Ankle	Control group 1 in lightest gray bar (-DTR +PBS) n = 12<br>Control group 2 in medium gray bar (+DTR +PBS) n = 6<br>Control group 3 in darkest gray bar (-DTR + DT) n = 9<br>Experimental group 4 in blue (+DTR +DT) n = 7<br>Each n is an individual mouse (biological replicates)	2 way ANOVA followed by Holm Sidak multiple comparisons	**ANOVA**<br>Treatment (PBS/DT): F = 4.20. DFn = 1, DFd = 30, P = 0.0493<br>Genotype (-DTR/+DTR): F = 11.60. DFn = 1, DFd = 30, P = 0.0019<br>Interaction: F = 23.57. DFn = 1, DFd = 30, P < 0.0001<br>**Multiple comparisons**<br>**2 vs 1:** Mean 1 = 74.33, Mean 2 = 68.46, Mean diff = 5.872, SE = 5.711, t = 1.028, DF = 30.00, Adj P value = 0.5267<br>**3 vs 1:** Mean 1 = 79.84, Mean 2 = 68.46, Mean diff = 11.38, SE = 5.037, t = 2.259, DF = 30.00, Adj P value = 0.0911<br>**4 vs 1:** Mean 1 = 46.35, Mean 2 = 68.46, Mean diff = -22.12, SE = 5.432, t = 4.071, DF = 30.00, Adj P value = 0.0013<br>**3 vs 2:** Mean 1 = 79.84, Mean 2 = 74.33, Mean diff = 5.504, SE = 6.020, t = 0.9143, DF = 30.00, Adj P value = 0.5267<br>**4 vs 2:** Mean 1 = 46.35, Mean 2 = 74.33, Mean diff = -27.99, SE = 6.355, t = 4.404, DF = 30.00, Adj P value = 0.0006<br>**4 vs 3:** Mean 1 = 46.35, Mean 2 = 79.84, Mean diff = -33.49, SE = 5.756, t = 5.818, DF = 30.00, Adj P value < 0.0001
Figure 1E, Knee	Control group 1 in lightest gray bar (-DTR +PBS) n = 12<br>Control group 2 in medium gray bar (+DTR +PBS) n = 6<br>Control group 3 in darkest gray bar (-DTR + DT) n = 9<br>Experimental group 4 in blue (+DTR +DT) n = 7<br>Each n is an individual mouse (biological replicates)	2 way ANOVA followed by Holm Sidak multiple comparisons	**ANOVA**<br>Treatment (PBS/DT): F = 26.79. DFn = 1, DFd = 30, P < 0.0001<br>Genotype (-DTR/+DTR): F = 26.96. DFn = 1, DFd = 30, P < 0.0001<br>Interaction: F = 43.47. DFn = 1, DFd = 30, P < 0.0001<br>**Multiple comparisons**<br>**2 vs 1:** Mean 1 = 88.47, Mean 2 = 82.84, Mean diff = 5.632, SE = 5.664, t = 0.9943, DF = 30.00, Adj P value = 0.5996<br>**3 vs 1:** Mean 1 = 88.54, Mean 2 = 82.84, Mean diff = 5.699, SE = 4.996, t = 1.141, DF = 30.00, Adj P value = 0.5996<br>**4 vs 1:** Mean 1 = 41.15, Mean 2 = 82.84, Mean diff = -41.69, SE = 5.388, t = 7.738, DF = 30.00, Adj P value < 0.0001<br>**3 vs 2:** Mean 1 = 88.54, Mean 2 = 88.47, Mean diff = 0.06727, SE = 5.971, t = 0.01127, DF = 30.00, Adj P value = 0.9911<br>**4 vs 2:** Mean 1 = 41.15, Mean 2= 88.47, Mean diff = -47.32, SE = 6.303, t = 7.509, DF = 30.00, Adj P value < 0.0001<br>**4 vs 3:** Mean 1 = 41.15, Mean 2 = 88.54, Mean diff = -47.39, SE = 5.709, t = 8.301, DF = 30.00, Adj P value < 0.0001
Figure 1H, Ankle Angle	Control group 1 in gray line n = 21<br>Experimental group 2 in blue line n = 4<br>Each n is an individual mouse (biological replicates)	2 way ANOVA followed by Holm Sidak multiple comparisons	**ANOVA**<br>Step cycle (%): F = 113.56. DFn = 24, DFd = 552, P value is < 0.0001<br>Genotype: F = 24.38. DFn = 1, DFd = 23, P value is < 0.0001<br>Interaction: F = 5.61. DFn = 24, DFd = 552, P value = 0.0006<br>**Multiple comparisons (only significant ones listed)**<br>At 12%: Mean 1 = 44.6, Mean 2 = 61.7, Mean diff = -17.1, SE = 2.93, t = 5.84, DF = 5.83, Adj P value = 0.0305
Figure 1K	Control group 1 in gray bar (littermate) n = 17<br>Experimental group 2 in green bar (cKO) n = 5<br>Each n is an individual mouse (biological replicates)	Unpaired two tailed t test	Mean 1 = 0.3632, Mean 2 = 0.1633, Mean diff = -0.1999, SEM = 0.02720, t = 7.350, df = 20, P value < 0.0001
Figure 1M, Ankle	Control group 1 in gray bar (littermate) n = 11<br>Experimental group in green bar (cKO) n = 7<br>Each n is an individual mouse (biological replicates)	Unpaired two tailed t test	Mean 1 = 61.72, Mean 2 = 54.49, Mean diff = -7.223, SEM = 4.599, t = 1.570, df = 16, P value = 0.1359

Figure 1M, Knee	Control group 1 in gray bar (littermate) n = 11<br>Experimental group in green bar (cKO) n = 7<br>Each n is an individual mouse (biological replicates)	Unpaired two tailed t test	Mean 1 = 85.04, Mean 2 = 72.88, Mean diff = -12.16, SEM = 5.742, t = 2.118, df = 16, P value = 0.0502
Figure 1O, Ankle Angle	Control group 1 in gray line n = 22<br>Experimental group 2 in green line n = 13<br>Each n is an individual mouse (biological replicates)	2 way ANOVA followed by Holm Sidak multiple comparisons	**ANOVA**<br>Step cycle (%): F = 425.05. DFn = 24, DFd = 792, P value is < 0.0001<br>Genotype: F = 4.93. DFn = 1, DFd = 33, P value = 0.0334<br>Interaction: F = 13.23. DFn = 24, DFd = 792, P value is < 0.0001<br>**Multiple comparisons (only significant ones listed)**<br>At 4%: Mean 1 = 103, Mean 2 = 89.3, Mean diff = 14.2, SE = 2.64, t = 5.36, DF = 27.7, Adj P value = 0.0002<br>At 84%: Mean 1 = 95.2, Mean 2 = 85.6, Mean diff = 9.62, SE = 2.67, t = 3.60, DF = 24.1, Adj P value = 0.0281<br>At 88%: Mean 1 = 102, Mean 2 = 90.4, Mean diff = 12.0, SE = 2.63, t = 4.57, DF = 23.9, Adj P value = 0.0026<br>At 92%: Mean 1 = 109, Mean 2 = 95.4, Mean diff = 14.0, SE = 2.58, t = 5.40, DF = 25.1, Adj P value = 0.0003<br>At 96%: Mean 1 = 115, Mean 2 = 100, Mean diff = 14.5, SE = 2.48, t = 5.86, DF = 26.7, Adj P value < 0.0001<br>At 100%: Mean 1 = 115, Mean 2 = 101, Mean diff = 14.1, SE = 2.16, t = 6.51, DF = 31.3, Adj P value < 0.0001
Figure 6D	n = 2 biological replicates of snRNA-sequencing data<br>Each n is pooled from 4 animals of each genotype (8 total animals)	scCODA (see Methods)	Used Cluster 1 as a reference and a FDR of 0.05. Only cluster 10 had a credible effect on cell type composition with a log2FC of -1.61 and an inclusion probability parameter of 0.99
Figure 6F, Pou6f2	Control group 1 in blue bar (En1 Het littermate) n = 7<br>Experimental group 2 in orange bar (En1 KO) n = 7<br>Each n is an individual mouse (biological replicates)	Unpaired two tailed t test	Mean 1 = 13.89, Mean 2 = 8.360, Mean diff = -5.530, SEM = 0.6384, t = 8.662, df = 12, P value < 0.0001
Figure 6F, Nr5a2	Control group 1 in blue bar (En1 Het littermate) n = 5<br>Experimental group 2 in orange bar (En1 KO) n = 5<br>Each n is an individual mouse (biological replicates)	Unpaired two tailed t test	Mean 1 = 6.344, Mean 2 = 0.1440, Mean diff = -6.200, SEM = 0.2982, t = 20.79, df = 8, P value < 0.0001
Figure 6G	Control group 1 in blue bar (En1 Het littermate) n = 3-5<br>Experimental group 2 in orange bar (En1 KO) n = 3-4<br>Each n is an individual mouse (biological replicates)<br>See "Results" for specific n size for each gene	Two tailed unpaired t test with Holm-Sidak correction for multiple comparisons	**Bhlhb5:** Mean 1 = 23.23, Mean 2 = 26.26, Mean diff = -3.033, SE = 2.992, t = 1.014, df = 4, P value = 0.3680, n 1 = 3, n 2 = 3<br>**Foxp1:** Mean 1 = 26.63, Mean 2 = 23.31, Mean diff = 3.323, SE = 3.581, t = 0.9280, df = 4.000, P value = 0.4059, n 1 = 3, n 2 = 3<br>**Foxp2:** Mean 1 = 35.29, Mean 2 = 40.61, Mean diff = -5.315, SE = 2.267, t = 2.345, df = 7.000, P value = 0.0515, n 1 = 5, n 2 = 4<br>**Foxp4:** Mean 1 = 13.56, Mean 2 = 12.86, Mean diff = 0.7025, SE = 1.846, t = 0.3806, df = 6.000, P value = 0.7166, n 1 = 4, n 2 = 4<br>**MafB:** Mean 1 = 20.38, Mean 2 = 17.44, Mean diff = 2.937, SE = 1.838, t = 1.598, df = 4.000, P value = 0.1853, n 1 = 3, n 2 = 3<br>**Nr3b3:** Mean 1 = 24.11, Mean 2 = 24.03, Mean diff = 0.08500, SE = 3.062, t = 0.02776, df = 6.000, P value = 0.9788, n 1 = 4, n 2 = 4<br>**OC2:** Mean 1 = 14.42, Mean 2 = 12.77, Mean diff = 1.647, SE = 1.128, t = 1.460, df = 4.000, P value = 0.2181, n 1 = 3, n 2 = 3<br>**Otp:** Mean 1 = 30.24, Mean 2 = 32.32, Mean diff = -2.080, SE = 1.369, t = 1.520, df = 4.000, P value = 0.2033, n 1 = 3, n 2 = 3<br>**Prdm8:** Mean 1 = 19.73, Mean 2 = 21.94, Mean diff = -2.207, SE = 1.496, t = 1.475, df = 4.000, P value = 0.2141, n 1 = 3, n 2 = 3<br>**Sp8:** Mean 1 = 13.23, Mean 2 = 13.76, Mean diff = -0.5300, SE = 1.270, t = 0.4173, df = 6.000, P value = 0.6910, n 1 = 4, n 2 = 4
Supplemental Figure 1B	Control group 1 in gray bar (PBS) n = 6<br>Experimental group 2 in blue (DT) n = 6<br>Each n is an individual mouse (biological replicates)	Unpaired two tailed t test	Mean 1 = 146.3, Mean 2 = 5.203, Mean diff = -141.1, SEM = 3.075, t=45.89, df=10, P value < 0.0001
Supplemental Figure 1C, Wrist	Control group 1 in lightest gray bar (-DTR +PBS) n = 12<br>Control group 2 in medium gray bar (+DTR +PBS) n = 6<br>Control group 3 in darkest gray bar (-DTR + DT) n = 7<br>Experimental group 4 in blue (+DTR +DT) n = 3<br>Each n is an individual mouse (biological replicates)	2 way ANOVA followed by Holm Sidak multiple comparisons	**ANOVA**<br>Treatment (PBS/DT): F = 9.93. DFn = 1, DFd = 24, P value = 0.0043<br>Genotype (-DTR/+DTR): F = 0.04. DFn = 1, DFd = 24, P value = 0.8509<br>Interaction: F = 0.16. DFn = 1, DFd = 24, P value = 0.6962<br>**Multiple comparisons**<br>**2 vs 1:** Mean 1 = 173.1, Mean 2 = 173.6, Mean diff = -0.4760, SE = 2.723, t = 0.1748, DF = 24.00, Adj P value = 0.9221<br>**3 vs 1:** Mean 1 = 165.4, Mean 2 = 173.6, Mean diff = -8.227, SE = 2.590, t = 3.177, DF = 24.00, Adj P value = 0.0241<br>**4 vs 1:** Mean 1 = 166.7, Mean 2 = 173.6, Mean diff = -6.869, SE = 3.515, t = 1.954, DF = 24.00, Adj P value = 0.2272<br>**3 vs 2:** Mean 1 = 165.4, Mean 2 = 173.1, Mean diff = -7.751, SE = 3.029, t = 2.559, DF = 24.00, Adj P value = 0.0833<br>**4 vs 2:** Mean 1 = 166.7, Mean 2 = 173.1, Mean diff = -6.393, SE = 3.850, t = 1.660, DF = 24.00, Adj P value = 0.2946<br>**4 vs 3:** Mean 1 = 166.7, Mean 2 = 165.4, Mean diff = 1.358, SE = 3.758, t = 0.3614, DF = 24.00, Adj P value = 0.9221

Supplemental Figure 1C, Elbow	Control group 1 in lightest gray bar (-DTR +PBS) n = 12<br>Control group 2 in medium gray bar (+DTR +PBS) n = 6<br>Control group 3 in darkest gray bar (-DTR + DT) n = 7<br>Experimental group 4 in blue (+DTR +DT) n = 3<br>Each n is an individual mouse (biological replicates)	2 way ANOVA followed by Holm Sidak multiple comparisons	**ANOVA**<br>Treatment (PBS/DT): F = 0.60. DFn = 1, DFd = 24, P value = 0.4465<br>Genotype (-DTR/+DTR): F = 0.33. DFn = 1, DFd = 24, P value = 0.5721<br>Interaction: F = 5.46. DFn = 1, DFd = 24, P value = 0.0281<br>**Multiple comparisons**<br>**2 vs 1:** Mean 1 = 160.1, Mean 2 = 153.7, Mean diff = 6.420, SE = 4.271, t = 1.503, DF = 24.00, Adj P value = 0.4676<br>**3 vs 1:** Mean 1 = 159.4, Mean 2 = 153.7, Mean diff = 5.688, SE = 4.063, t = 1.400, DF = 24.00, Adj P value = 0.4676<br>**4 vs 1:** Mean 1 = 148.8, Mean 2 = 153.7, Mean diff = -4.902, SE = 5.514, t = 0.8890, DF = 24.00, Adj P value = 0.6191<br>**3 vs 2:** Mean 1 = 159.4, Mean 2 = 160.1, Mean diff = -0.7320, SE = 4.752, t = 0.1540, DF = 24.00, Adj P value = 0.8789<br>**4 vs 2:** Mean 1 = 148.8, Mean 2 = 160.1, Mean diff = -11.32, SE = 6.040, t = 1.875, DF = 24.00, Adj P value = 0.3658<br>**4 vs 3:** Mean 1 = 148.8, Mean 2 = 159.4, Mean 2 = -10.59, SE = 5.895, t = 1.797, DF = 24.00, Adj P value = 0.3658
Supplemental Figure 1F	Control group 1 in gray bar (littermate) n = 7<br>Experimental group 2 in red bar (En1 KO) n = 6<br>Each n is an individual mouse (biological replicates)	Unpaired two tailed t test	Mean 1 = 0.3483, Mean 2 = 0.1014, Mean diff = -0.2469, SEM = 0.02238, t=11.04, df=11, P value < 0.0001
Supplemental Figure 1H	Control group 1 in gray bar (littermate) n = 4<br>Experimental group 2 (En1 cKO) n = 3<br>Each n is an individual mouse (biological replicates)	Unpaired two tailed t test	Mean 1 = 62.45, Mean 2 = 0.000, Mean diff = -62.45, SEM = 3.929, t=15.89, df=5, P value < 0.0001
Supplemental Figure 1K	Control group 1 in lightest gray bar (-DTR +PBS) n = 6<br>Control group 2 in medium gray bar (+DTR +PBS) n = 4<br>Control group 3 in darkest gray bar (-DTR + DT) n = 6<br>Experimental group 4 in blue (+DTR +DT) n = 4<br>Each n is an individual mouse (biological replicates)	2 way ANOVA followed by Holm Sidak multiple comparisons	**ANOVA**<br>Treatment (PBS/DT): F = 10.02. DFn = 1, DFd = 16, P value = 0.0060<br>Genotype (-DTR/+DTR): F = 17.88. DFn = 1, DFd = 16, P value = 0.0006<br>Interaction: F = 23.72. DFn = 1, DFd = 16, P value = 0.0002<br>**Multiple comparisons**<br>**2 vs 1:** Mean 1 = 0.3247, Mean 2 = 0.3113, Mean diff = 0.01339, SE = 0.02948, t = 0.4542, DF = 16.00, Adj P value = 0.7119<br>**3 vs 1:** Mean 1 = 0.3469, Mean 2 = 0.3113, Mean diff = 0.03555, SE = 0.02637, t = 1.348, DF = 16.00, Adj P value = 0.4810<br>**4 vs 1:** Mean 1 = 0.1572, Mean 2 = 0.3113, Mean diff = -0.1541, SE = 0.02948, t = 5.228, DF = 16.00, Adj P value = 0.0004<br>**3 vs 2:** Mean 1 = 0.3469, Mean 2 = 0.3247, Mean diff = 0.02216, SE = 0.02948, t = 0.7516, DF = 16.00, Adj P value = 0.7119<br>**4 vs 2:** Mean 1 = 0.1572, Mean 2 = 0.3247, Mean diff = -0.1675, SE = 0.03229, t = 5.187, DF = 16.00, Adj P value = 0.0004<br>**4 vs 3:** Mean 1 = 0.1572, Mean 2 = 0.3469, Mean diff = -0.1897, SE = 0.02948, t = 6.434, DF = 16.00, Adj P value < 0.0001
Supplemental Figure 1N	Control group 1 in lightest gray bar (-DTR +PBS) n = 6<br>Control group 2 in medium gray bar (+DTR +PBS) n = 4<br>Control group 3 in darkest gray bar (-DTR + DT) n = 10<br>Experimental group 4 in purple (+DTR +DT) n = 6<br>Each n is an individual mouse (biological replicates)	2 way ANOVA followed by Holm Sidak multiple comparisons	**ANOVA**<br>Treatment (PBS/DT): F = 3.92. DFn = 1, DFd = 22, P value = 0.0604<br>Genotype (-DTR/+DTR): F = 0.01. DFn = 1, DFd = 22, P value = 0.9362<br>Interaction: F = 4.39e-003. DFn = 1, DFd = 22, P value = 0.9478<br>**Multiple comparisons**<br>**2 vs 1:** Mean 1 = 0.3300, Mean 2 = 0.3333, Mean diff = -0.003333, SE = 0.03538, t = 0.09423, DF = 22.00, Adj P value = 0.9945<br>**3 vs 1:** Mean 1 = 0.2870, Mean 2 = 0.3333, Mean diff = -0.04633, SE = 0.02830, t = 1.637, DF = 22.00, Adj P value = 0.5222<br>**4 vs 1:** Mean 1 = 0.2867, Mean 2 = 0.3333, Mean diff = -0.04667, SE = 0.03164, t = 1.475, DF = 22.00, Adj P value = 0.5677<br>**3 vs 2:** Mean 1 = 0.2870, Mean 2 = 0.3300, Mean diff = -0.04300, SE = 0.03242, t = 1.326, DF = 22.00, Adj P value = 0.5870<br>**4 vs 2:** Mean 1 = 0.2867, Mean 2 = 0.3300, Mean diff = -0.04333, SE = 0.03538, t = 1.225, DF = 22.00, Adj P value = 0.5870<br>**4 vs 3:** Mean 1 = 0.2867, Mean 2 = 0.2870, Mean diff = -0.0003333, SE = 0.02830, t = 0.01178, DF = 22.00, Adj P value = 0.9945
Supplemental Figure 5G	Group 1 in gray bar (L3/L4) n = 7<br>Group 2 in gray bar (L5) n = 4<br>Each n is an individual mouse (biological replicates)	Unpaired two tailed t test	Mean 1 = 2.317, Mean 2 = 39.98, Mean diff = 37.66, SEM = 2.024, t = 18.60, df=9, P value < 0.0001
Supplemental Figure 7A	Control group 1 in light gray bar (En1 Het) n = 5<br>Experimental group 2 in dark gray bar (En1 KO) n = 5<br>Each n is an individual mouse (biological replicates)	Unpaired two tailed t test	Mean 1 = 267.6, Mean 2 = 265.0, Mean diff = -2.600, SEM = 10.77, t = 0.2413, df = 8, P value = 0.8154
Supplemental Figure 7D	Control group 1 in gray bar (Littermate) n = 3<br>Experimental group 2 in green bar (En1 cKO) n = 5<br>Each n is an individual mouse (biological replicates)	Unpaired two tailed t test	Mean 1 = 16.09, Mean 2 = 7.541, Mean diff = -8.553, SEM = 1.025, t = 8.345, df = 6, P value = 0.0002

Supplemental Figure 7F, Total V1 population	Control group 1 in black (En1 Het) V1 n = 1775 cells from 4 animals<br>Experimental group 2 in red (En1 KO) V1 n = 1702 cells from 5 animals	2-dimensional Kolmogorov-Smirnov test	D statistic = 0.146, P value = 3.23e-11
Supplemental Figure 7F, Pou6f2 V1	Control group 1 in black (En1 Het) Pou6f2 n = 309 neurons from 8 animals<br>Experimental group 2 in red (En1 KO) Pou6f2 n = 213 neurons from 5 animals	2-dimensional Kolmogorov-Smirnov test	D statistic = 0.394, P value = 6.53e-13
Supplemental Figure 7F, Sp8 V1	Control group 1 in black (En1 Het) Sp8 n = 122 neurons from 3 animals<br>Experimental group 2 in red (En1 KO) Sp8 n = 113 neurons from 3 animals	2-dimensional Kolmogorov-Smirnov test	D statistic = 0.185, P value = 0.0949
