# Supplementary material for: Transcriptomic analysis of spinal V1 interneurons informs their multifunctional role in motor output
Source: Nat Commun. 2026 Aug 10;17:9614. doi: 10.1038/s41467-026-76522-3 (PMC13554063; doi:10.1038/s41467-026-76522-3)
Supplement: Supplementary file 4 [file 41467_2026_76522_MOESM2_ESM.pdf]

Description of Additional Supplementary Files

Supplementary Video 1. Examples of tail suspension assay in control, V1 ablated, and En1 cKO mice.

Supplementary Video 2. Examples of adult locomotion in control, V1 ablated, and En1 cKO mice.

Supplementary Data 1. Quality control metrics for CellRanger.

Supplementary Data 2. Cell-type classification markers.

Supplementary Data 3. Cell barcodes of analyzed V1 nuclei.

Supplementary Data 4. Differentially expressed genes in V1 clusters.

Supplementary Data 5. Differentially expressed genes by developmental age.

Supplementary Data 6. Differentially expressed genes in V1 clusters in the En1Het versus En1KO experiment.
